# Sodium formononetin-3'-sulphonate alleviates cerebral ischemia–reperfusion injury in rats via suppressing endoplasmic reticulum stress-mediated apoptosis

**DOI:** 10.1186/s12868-022-00762-4

**Published:** 2022-12-09

**Authors:** Yue Bai, Zhiwei He, Weisong Duan, He Gu, Kefeng Wu, Wei Yuan, Wenkang Liu, Huaipeng Huang, Yanan Li

**Affiliations:** 1 Department of Internal Medicine, Shijiazhuang Pingan Hospital, Hebei Medical University, Shijiazhuang, 050000 Hebei China; 2 Neurological Laboratory of Hebei Province, The Second Hospital of Hebei Medical University, Shijiazhuang, 050000 Hebei China; 3 Department of Clinical Laboratory Diagnosis, Shijiazhuang Pingan Hospital, Hebei Medical University, Shijiazhuang, 050000 Hebei China

**Keywords:** Formononetin, Cerebral ischemia-reperfusion injury, Endoplasmic reticulum stress, Cell apoptosis, Ischemic penumbra, Stroke

## Abstract

**Background:**

Sodium formononetin-3ʹ-sulphonate (Sul-F) may alleviate I/R injury in vivo with uncertain mechanism. Endoplasmic reticulum (ER) stress-mediated apoptosis participates in the process of cerebral ischemia‐reperfusion (I/R) injury. Our aim is to figure out the effect of Sul-F on cerebral I/R injury and to verify whether it works through suppressing ER stress-mediated apoptosis.

**Results:**

The cerebral lesions of middle cerebral artery occlusion (MCAO) model in SD rats were aggravated after 24 h of reperfusion, including impaired neurological function, increased infarct volume, intensified inflammatory response and poor cell morphology. After intervention, the edaravone (EDA, 3 mg/kg) group and Sul-F high-dose (Sul-F-H, 80 mg/kg) group significantly alleviated I/R injury via decreasing neurological score, infarct volume and the serum levels of inflammatory factors (TNF-α, IL-1β and IL-6), as well as alleviating pathological injury. Furthermore, the ER stress level and apoptosis rate were elevated in the ischemic penumbra of MCAO group, and were significantly blocked by EDA and Sul-F-H. In addition, EDA and Sul-F-H significantly down-regulated the ER stress related PERK/eIF2α/ATF4 and IRE1 signal pathways, which led to reduced cell apoptosis rate compared with the MCAO group. Furthermore, there was no difference between the EDA and Sul-F-H group in terms of therapeutic effect on cerebral I/R injury, indicating a therapeutic potential of Sul-F for ischemic stroke.

**Conclusions:**

Sul-F-H can significantly protects against cerebral I/R injury through inhibiting ER stress-mediated apoptosis in the ischemic penumbra, which might be a novel therapeutic target for ischemic stroke.

**Supplementary Information:**

The online version contains supplementary material available at 10.1186/s12868-022-00762-4.

## Introduction

Ischemic stroke is a common central nervous system disease and a common cause of death and disability around the world [1]. Ischemic stroke accounts for about 70% of all strokes, which is caused by insufficient blood supply and results in the immediate depletion of oxygen and glucose in brain tissue [2]. Timely restoration of blood flow and reoxygenation remain the widely accepted methods, although considerable progress has been made in the treatment of cerebral ischemia [3].

In terms of treatment, tissue plasminogen activator (t-PA) is the only thrombolytic drug approved by the Food and Drug Administration for the therapy of ischemic stroke, which dissolves thrombus by activating a proteolytic enzyme [4]. Early use of thrombolytic agents is beneficial for the recovery, rehabilitation and prognosis of acute ischemic stroke patients. However, there are still many obstacles that blocked the application of the thrombolytic drugs, such as narrow therapeutic window and high risk of hemorrhagic transformation [5, 6]. In addition, the cerebral I/R injury may occur after the restoration of blood circulation in the ischemic brain, seriously affecting neurons and ultimately leading to neuronal apoptosis [7]. Therefore, it is still necessary to explore drugs with wide clinical adaptability and high safety.

In China, traditional Chinese medicine (TCM) is widely used in the management of ischemic stroke, based on the advantage of promoting blood circulation to dissipate blood stasis. Buyang Huanwu Decoction (BHD), a classic prescription for stroke treatment, plays a protective role in cerebral I/R injury in vivo by promoting neurogenesis [8, 9], inhibiting neural apoptosis and alleviating inflammation [10], stimulating angiogenesis and improving cerebral circulation [11]. Accumulating evidences have revealed that multiple components of BHD could ameliorate the negative effect of stroke [12–16]. Formononetin (C_16_H_12_O_4_), an isoflavone compound separated from BHD, has demonstrated diverse pharmacological capabilities, including neuroprotection [17], anti-inflammation [18], anti-oxidative stress [19], anti-apoptotic [20], and anti-tumor [21, 22]. However, poor water solubility limited the bioavailability of formononetin in central nervous system.

Subsequently, Sul-F (C_16_H_11_O_7_SNa, Chinese patent: ZL200710017326.5), a sulfonated derivative of formononetin, has been synthesized and overcomes the above disadvantage. Recent researches revealed that Sul-F exerted beneficial effects in multiple cardiovascular and cerebrovascular diseases, including acute myocardial infarction [23]and stroke [24] in animal models. Moreover, Sul-F can reduce permeability of blood brain barrier (BBB) after cerebral ischemic injury as well as possess the effect of anti-apoptosis and anti-thrombosis [25]. These studies suggested that Sul-F maybe not only a potentially effective drug for the treatment of ischemic stroke, but also a gospel of patients with ischemic stroke. Thus, it is of high importance to evaluate the benefits of Sul-F administration and the underlying mechanisms.

The mechanisms related to cerebral I/R injury are still far from clear and an effective prevention for cerebral I/R injury has not been established yet. Recent findings have shown that endoplasmic reticulum (ER) stress is an important signal pathway of neuronal injury caused by cerebral I/R injury [26–29]. ER plays vital roles in protein translocation, modification and folding, which is an essential organelle in eukaryotic cells [30]. When subjected to various strong stimulating factors, including nutrient deficiencies, Ca^2+^ metabolic imbalance, toxin stimulation and sustained oxidative stress stimulation, the cell homeostasis will be broken, which further leads to the massively accumulation of the misfolded and unfolded proteins in ER. Thus, the ER stress and unfolded protein response (UPR) will be initiated to help the misfolded and unfolded proteins restore to its normal structure through the activation of PERK, IRE1, and ATF4. By these processes, ER stress rebalances intercellular homeostasis and protects cells from various stimulus. However, overly-activated ER stress and UPR can cause damages [31]. Increased ER stress is observed in the ischemic penumbra of cerebral I/R injury rat model [32, 33]. Besides, inhibiting ER stress with compounds can significantly protect neurons against ischemic injury [26, 29, 34]. Even though evidences indicate that Sul-F is involved in the neuron protection against focal cerebral I/R injury [1], whether it can maintain ER homeostasis and reduce ER stress mediated neuronal apoptosis in cerebral I/R injury rat is still unknown.

In this study, we investigated whether Sul-F treatment could protect neuron against cerebral I/R injury. Further, we explored the protective effects of Sul-F through inhibiting apoptosis in penumbra. Finally, the underlying mechanism of its anti-apoptosis ability was further revealed.

## Materials and methods

### Chemicals

Edaravone injection was purchased from Sinopharm Group Guorui Pharmaceutical Co. LTD (China). Sul-F (> 95% pure) was bought from Shijiazhuang Hairui Pharmaceutical Technology Co. LTD (China). 2,3,5-triphenylte-trazolium chloride (TTC) was acquired from Sigma (USA). Hematoxylin-eosin staining (HE) kit and terminal deoxynucleotidyl transferase mediated dUTP-biotin nick end labeling (TUNEL) kit were purchased from Biyuntian Biotechnology Co. LTD (China). Bcl-2, Caspase3, Bax, CHOP, p-PERK, p-eIF2α, p-IRE1, Caspase12 and ATF4 primary antibodies were purchased from Affinity (China).

### Animals

All male Sprague-Dawley (SD) rats (grade SPF) were purchased from the Sibford Co. LTD (Beijing, China). The rats of 290-310 g (8-10 weeks old) were supplied with freely accessible food and water, and were housed in an environment with standard lighting conditions (12 h light/dark cycle), controlled temperature (20-25 °C) and humidity (40-60%). Before building MCAO model, all rats were fasted 12 h with freely accessible water.

### Middle cerebral artery occlusion (MCAO)

MCAO rat model was established with an intraluminal filament method as previously described [35]. After the rat was anesthetized with 2% sodium pentobarbital (0.3 mL/100 g) intraperitoneally (i.p), the anterior cervical region was exposed and opened along the midline of the neck to isolate the left common carotid artery (CCA), external carotid artery (ECA) and internal carotid artery (ICA). The ECA was ligated and CCA was clipped with an arterial clamp. In order to block the blood supply of the left middle cerebral artery, the monofilament nylon suture with a round tip was inserted into ICA via CCA with a depth of 18 ~ 20 mm. After 2 h of ischemia, the suture plug was removed about 0.5 cm and the blood perfusion was restored. The sham operation group was objected to the same operation without inserting a monofilament. The presence of neurological deficit was measured by Zea-Longa method and score point of 1-3 indicated successful modeling and inclusion in the experiment. The specific scoring criteria was as follows: 0, no neurological deficit; 1, failed to fully extend their left forepaw; 2, circling to the left when walking; 3, falling to the left when walking; 4, unable to walk spontaneously or has stroke-related death.

### Animal grouping and drug administration

Based on experimental target and the principle of randomization, all the rats were divided into 5 groups including sham group (Sham group), ischemia-reperfusion group (MCAO group), edaravone group (EDA group, 3 mg/kg), Sul-F high dose group (Sul-F-H group, 80 mg/kg) and Sul-F low dose group (Sul-F-L group, 40 mg/kg). All rats were given the drug for the first time at 0 h of reperfusion by tail vein injection with a volume of 4 mL/kg. The concentration of EDA injection was 30 mg/40 mL, and the dose was 3 mg/kg. The concentration of Sul-F low dose group was 200 mg/20 mL and the dose was 40 mg/kg. Sul-F high-dose group was 400 mg/20 mL and 80 mg/kg. Rats in MCAO group and Sham group were injected with equal volume of normal saline via tail vein. Rats in each group were given the second dose at 12 h reperfusion.

### Neurological impairment score

According to the blind principle, neurological deficiency was evaluated by the trained investigators after 24 h of reperfusion. Neurological behaviors of all rats were evaluated by a 5-point scale, as referred previously [36]. The higher the score, the more serious nerve function injury is.

### Histopathological examination

At 24 h post reperfusion, the rats were anesthetized with 2% sodium pentobarbital (0.3 mL/100 g) i.p and then underwent quick decapitation. A portion of rats’ brain tissue was dissected out and fixed in 4% paraformaldehyde for 48 h. At the end of fixation, tissue processing was done to dehydrate in ascending grades of alcohol, clearing in xylene and embedded in paraffin wax. Paraffin wax embedded tissue blocks were sectioned at 4 µm thickness with the Rotary Microtome (Leica, Germany). All the slides of brains were stained with HE. Then all the pathological changes were observed under optical microscope (Leica, Germany).

### TTC staining

**Fig. 1:**
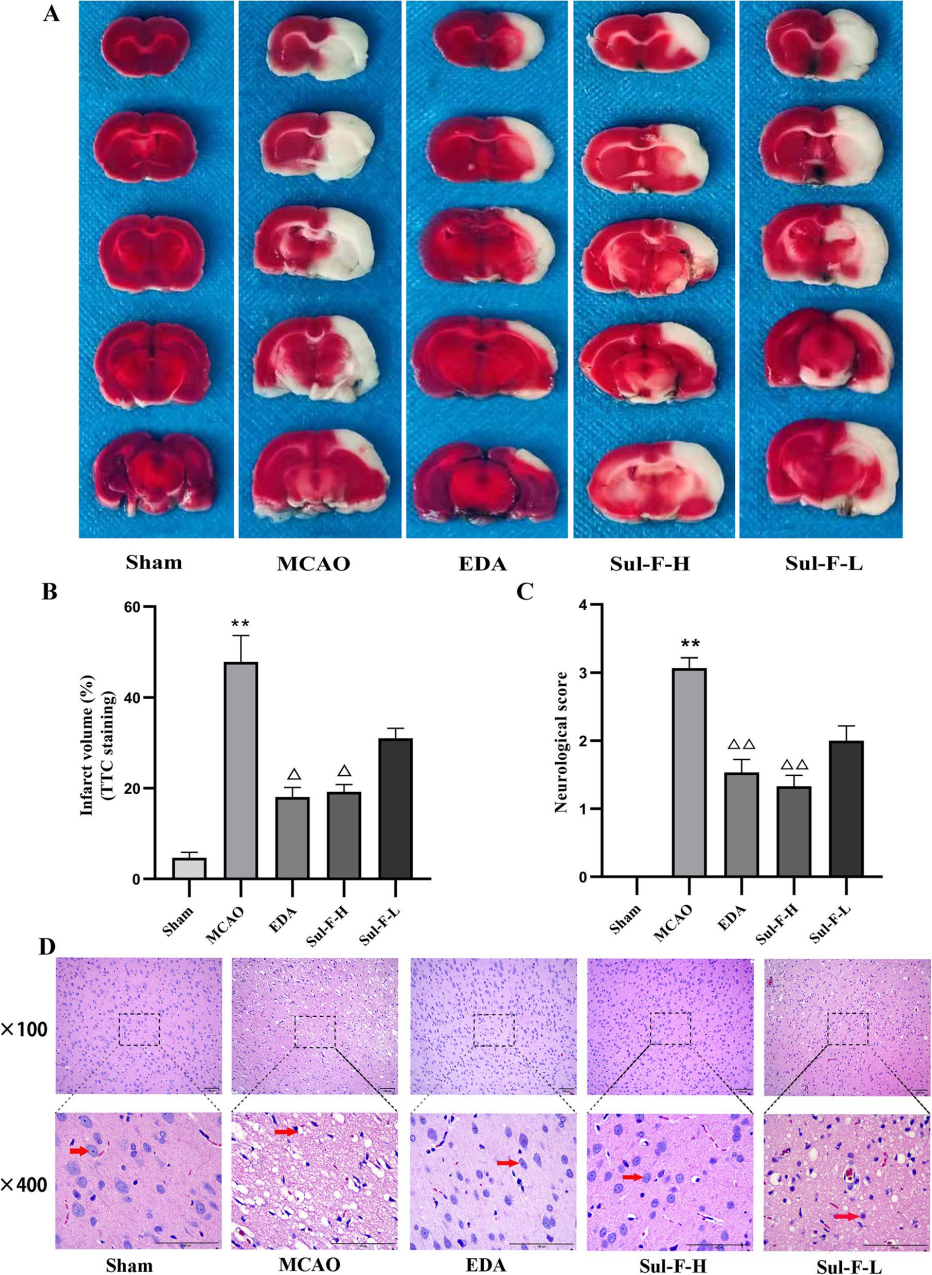
The effects of Sul-F treatment on infarct volume, neurological score and pathological changes of I/R injured brains. **A** Infarct volume was determined with TTC staining. The white area defined the infarct area (n = 6). **B** The infarct volume was expressed as the ratio of (infarct volume / the whole brain volume) × 100% (n = 6). **C** Neurological score (n = 15). The neurological function of the rats after 24 h of reperfusion was evaluated according to the Zea-Longa score standard. The higher the score, the more severe the neurological impairment is. Neurological score data was presented as *M* (*P*_25_ ~ *P*_75_). ^**^P < 0.01 compared with the Sham group, ^△△^*P* < 0.01 compared with the MCAO group. Data were presented as mean ± SEM from at least three independent experiments. ^**^P < 0.01 compared with Sham group, ^△^*P* < 0.05 and ^△△^*P* < 0.01 compared with the MCAO group. **D** Histopathological characteristics (n = 6). After 24 h reperfusion, the ischemic penumbra area of brain tissue was stained with HE, which was observed at 100 × and 400 × , respectively. Scale bar = 100 μm

The experiment was conducted according to previous study [1]. When neurological deficit examination was completed, the rats were deeply anesthetized with 2% sodium pentobarbital, then brains were taken out and sectioned coronally with a thickness of 2 mm after freezing in -20 °C refrigerator for 20 min. Before fixed with 4% paraformaldehyde overnight at room temperature, the brain tissue slices were stained with 2% 2, 3, 5-triphenyl tetrazolium chloride (TTC) for 0.5 h at room temperature in the dark. The results showed that the surviving part of the brain section was red, while the dead part was pale. Image J (Version 1.49) was used to measure the infarct area and the whole area of each brain slice (Fig. 1A). The infarct volume ratio was calculated as follows: infarct volume ratio % = (infarct volume / whole brain volume) × 100%.

### ELISA analysis

The blood collected from the aortaventralis was clotted in centrifugal tube (Henan Wenmei Experiment Co. Ltd, Henan, China) in room temperature for 20 min and centrifuged at 12,000 rpm at 4 ℃ for 15 min to obtain the serum. The levels of TNF-α, Interleukin-6 (IL-6), and IL-1β in the serum were measured using ELISA kits (Beijing Bioske Biomedical Technology Co., Ltd, Beijing, China) according to the manufacturer’s instructions.

### TUNEL staining

TUNEL staining was used to detect neuronal apoptosis. Briefly, the brain slices were dewaxed and rehydrated. Then, in order to block endogenous peroxidase activity, the sections were incubated with a methanol solution containing 3% H_2_O_2_ for 10 min at room temperature. Afterward, treated with TUNEL reaction mixture, the brain sections were maintained in a 37 °C incubator for 1 h. The fluorescence was captured with a laser confocal microscopy (Leica, Germany). The results were presented as apoptosis ratio = (TUNEL positive cells)/(DAPI cells) × 100%.

### Western blot analysis

Ischemic penumbra samples were obtained from ischemic hemisphere and preserved at -80 ℃. The BCA protein assay kit (MDL, Beijing, China) was used to measure the protein concentration of brain samples after the tissue was homogenized and lysed. Equal amounts of proteins were separated by sodium dodecyl sulfate polyacrylamide gel and transferred to polyvinylidene fluoride (PVDF) membrane. After blocking with 5% non-fat milk, PVDF membrane was incubated with primary antibody in 4 ℃ overnight. Then, the membrane was washed three times with Tris-buffered saline with Tween 20 (TBST). Thereafter, membranes were incubated with horseradish peroxidase-labeled secondary antibody for 2 h at room temperature. After incubation, the membranes were washed three times again with TBST. Afterwards, chemiluminescence imaging system (Clinx, Shanghai, China) was used to image and detect blots. All protein bands were quantitated by Image-Pro Plus.

### Quantitative real‑time polymerase chain reaction (qRT-PCR) analysis

Trizol (Invitrogen, USA) was used to extract total RNA from ischemic penumbra tissue and SuperScript III Reverse Transcription Kit (ABI-invitrogen, USA) was used to prepare cDNA. All qRT-PCR reactions were conducted by ABI PRISM 7500 Sequence Detector System (Applied Biosystems, CA, USA). Relative gene expression was quantified via the 2^−ΔΔCt^ approach. The primer sequences used in the qRT-PCR were as Additional file 1: Table S1.

### Statistical analysis

All data was displayed in mean ± standard error of the mean (SEM) and analyzed by SPSS (version 20.0, Chicago, USA). The normality of the data was checked with Shapiro-Wilk normality test, while P > 0.05 was considered to fit a normal distribution. Student’s *t*-test was used to analyze the statistical significance between two groups, one-way analysis of variance (ANOVA) was used to analyze the statistical significance among three or more groups. P value < 0.05 was considered statistically significant (Additional files 2, 3).

## Results

### Effect of Sul-F on cerebral I/R injury

TTC assay was used to detect the brain infarct volume and Zea-Longa score was applied to evaluate the neurological deficiency. HE staining was used to investigate the histopathological changes in the brain. Evidently, the infarct volume and neurological score significantly increased in the MCAO group (47.88 ± 14.07% vs. 4.67 ± 2.94% of Sham group, *P* < 0.01; 3(3 ~ 3) vs. 0(0 ~ 0) of Sham group, *P* < 0.01). Compared with the MCAO group, the administration of EDA and Sul-F significantly decreased the infarct volume and neurological score (18.10 ± 5.08 and 21.26 ± 5.06% vs. 47.88 ± 14.07%, *P* < 0.05; 2(1 ~ 2) and 1(1 ~ 2) vs. 3(3 ~ 3), *P* < 0.01) (Fig. 1B, C). There was no significant difference between the EDA group and the Sul-F-H group (18.10 ± 5.08 vs. 21.26 ± 5.06, *P* > 0.05; 2(1 ~ 2) vs. 2(1 ~ 2), *P* > 0.05).

As shown in Fig. 1D (red narrow), swelling and pyknosis in cytoplasm and morphologic changes of apoptosis with karyopyknosis, karyorrhexis and apoptotic body were found in the MCAO group. The intervention of EDA and Sul-F retained the basic structure of neurons and significantly reduced the morphologic changes of nerve cells. Moreover, high dose Sul-F exhibited a better effect than that of low dose.

### Effects of Sul-F on inflammation

**Fig. 2:**
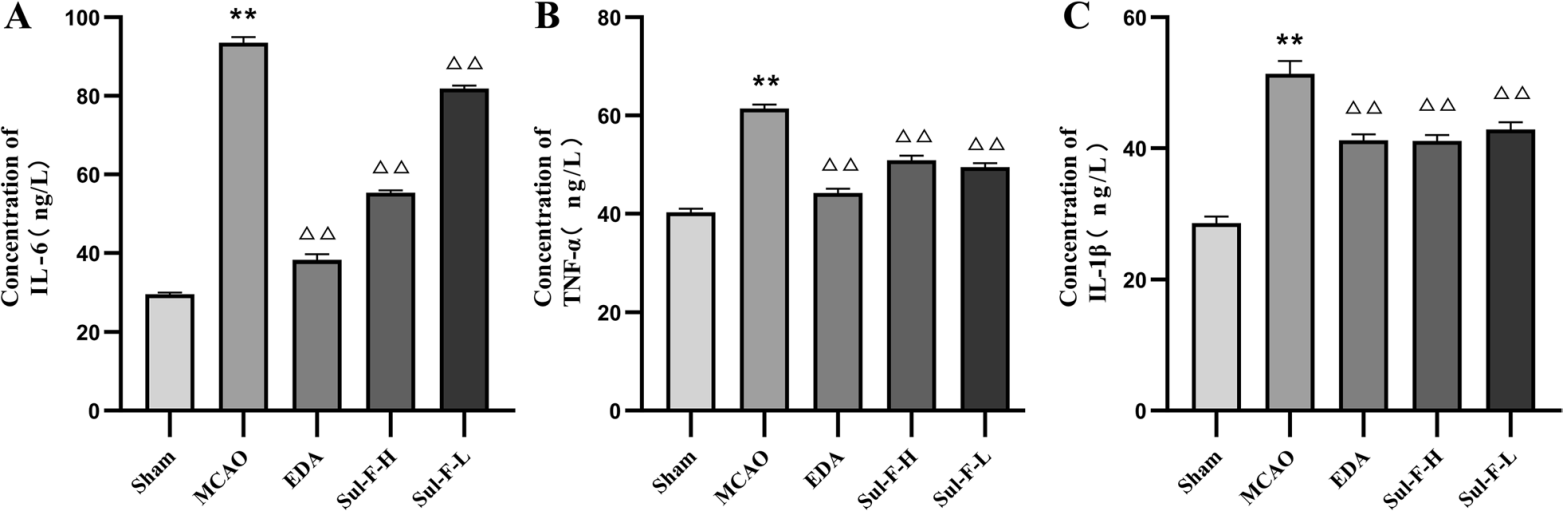
Sul-F treatment significantly alleviated the I/R-induced serum levels of inflammatory factors including TNF-α, IL-6 and IL-1β, detected by ELISA (n = 13). **A** The concentration of IL-6 in serum of rats. **B** The concentration of TNF-α in serum of rats. **C** The concentration of IL-1β in serum of rats. Data were presented as mean ± SEM from at least three independent experiments. **P < 0.01 compared with the Sham group, ^△△^*P* < 0.01 compared with the MCAO group

The levels of TNF-α, IL-1β and IL-6 were increased when cerebral I/R injury occurred (TNF-α: 61.45 ± 2.76 vs. 39.82 ± 3.13 of Sham group, *P* < 0.01; IL-1β: 51.38 ± 7.03 vs. 27.58 ± 3.82 of Sham group, *P* < 0.01; IL-6: 93.53 ± 5.03 vs. 29.43 ± 1.54 of Sham group, *P* < 0.01). Further, the levels of TNF-α, IL-1β and IL-6 were decreased in the EDA, Sul-F-H (80 mg/kg) and Sul-F-L (40 mg/kg) groups, compared to the MCAO group (Fig. 2, *P* < 0.01).

### Sul-F attenuated cell apoptosis via inhibiting ERS induced by MCAO

**Fig. 3:**
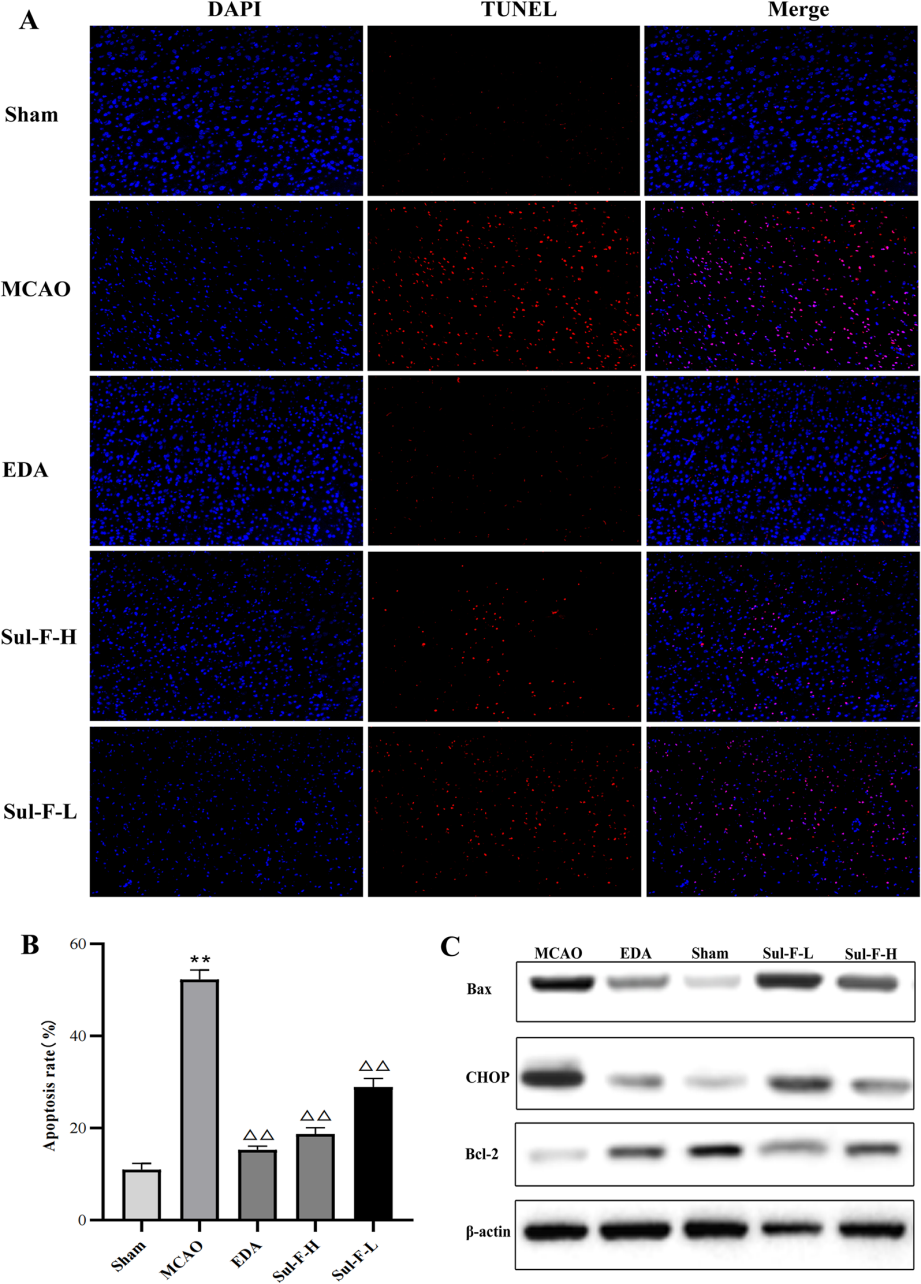

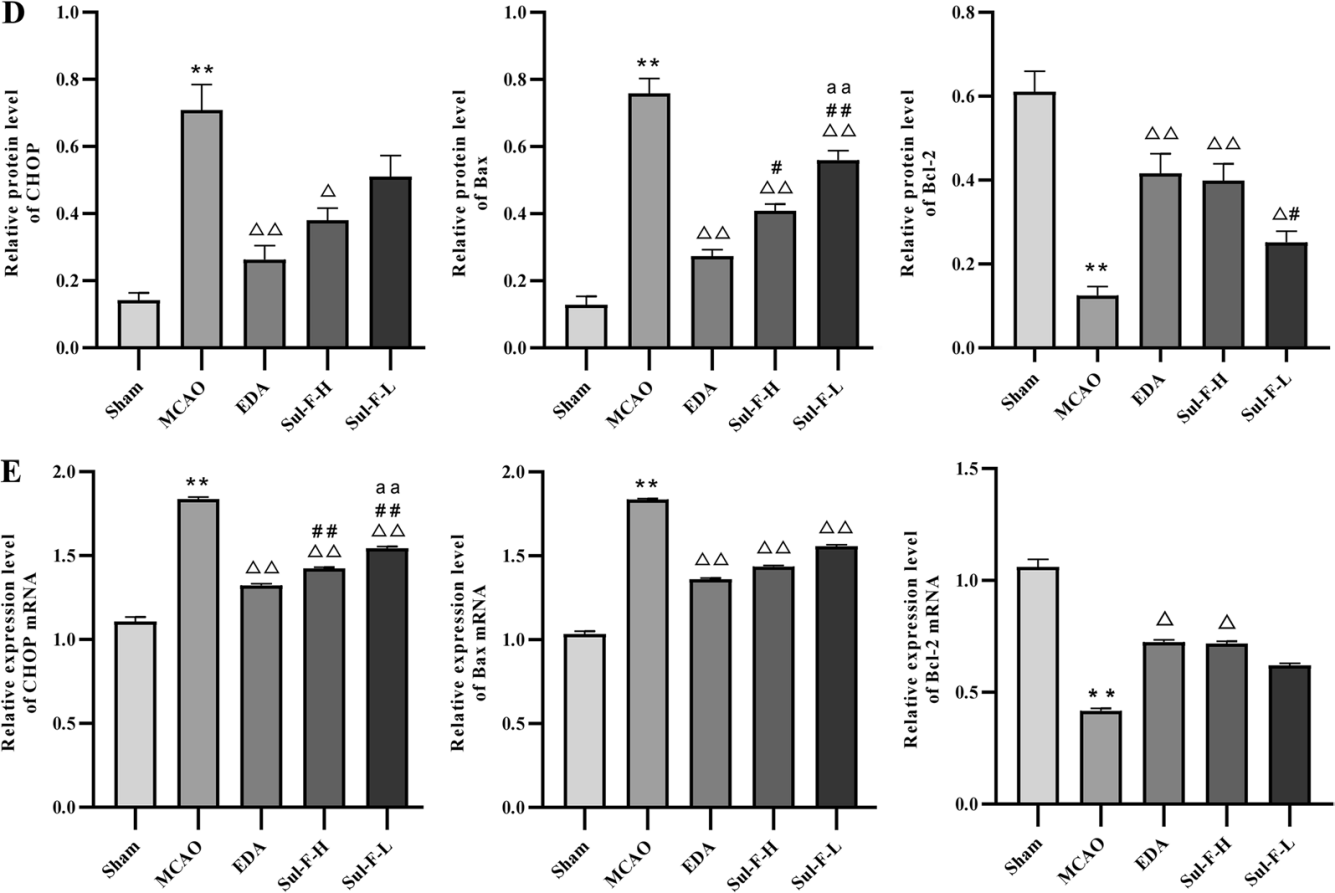
Sul-F treatment significantly alleviated the I/R-induced apoptosis in the penumbra of brain after 24 h reperfusion. **A** TdT-mediated dUTP Nick-End Labeling (TUNEL) staining of I/R-induced apoptosis in penumbra, which was imaged at 100 × . TUNEL cells (red) and the nuclei (DAPI, blue). **B** The I/R-induced apoptosis presented as ratio = (TUNEL positive cells) / (DAPI cells) × 100% (n = 5). **C** Representative western blot analysis of CHOP, Bax (pro-apoptotic protein) and Bcl-2 (anti-apoptotic protein) in the penumbra of brain tissue (n = 6). **D** Histogram showing quantification of images in **C** (n = 6). The results were normalized to β-actin expression. **E** Histogram showing quantification of qRT-PCR (n = 6). Data were expressed as the mean ± SEM from different assays. ^**^P < 0.01 compared with the Sham group, ^△^*P* < 0.05 and ^△△^*P* < 0.01 compared with the MCAO group, ^#^*P* < 0.05 and ^##^*P* < 0.01 compared with the EDA group, ^aa^*P* < 0.01 compared with the Sul-F-H group

Damage of nerve cells and impairment of neurological function were observed in MCAO rats and Sul-F alleviated the injury of nerve cells and improved the neurological function (Fig. 1). Furthermore, we examined cell apoptosis in the ischemic penumbra by TUNEL staining and Western blot. As shown in Fig. 3A, B, compared with the Sham group, abundant apoptotic cells were found in the penumbra of rats in the MCAO group (52.26 ± 2.06 vs. 10.99 ± 1.36, *P* < 0.01). However, the apoptotic rate was significantly reduced after the administration of Sul-F and EDA.

To further detect the molecular mechanism of Sul-F on apoptosis, we measured the expression of proteins associated with apoptosis (pro-apoptotic protein: Bax; anti-apoptotic protein: Bcl-2; the marker of apoptosis induced by ER stress: CHOP) by Western blot. Compared with the Sham group, the protein expression levels of Bax, and CHOP significantly increased (Bax: 0.76 ± 0.04 vs. 0.13 ± 0.03 of Sham group, *P* < 0.01; CHOP: 0.71 ± 0.08 vs. 0.14 ± 0.02 of Sham group, *P* < 0.01), and those of Bcl-2 decreased in the MCAO group (0.13 ± 0.02 vs. 0.61 ± 0.05 of Sham group, *P* < 0.01). Sul-F-H treatment resulted in the reduction of Bax (0.41 ± 0.02 vs. 0.76 ± 0.04 of MCAO group, *P* < 0.01), and CHOP (0.38 ± 0.04 vs. 0.71 ± 0.08 of MCAO group, *P* < 0.05), as well as the increase of Bcl-2 (0.40 ± 0.04 vs. 0.13 ± 0.02 of MCAO group, *P* < 0.01) level compared with the MCAO group (Fig. 3C, D). In addition, the mRNA expression levels of CHOP, Bax and Bcl-2 were detected by qRT-PCR in all specimens (n = 5). Increasing levels of CHOP mRNA (1.84 ± 0.01 vs. 1.11 ± 0.03 of Sham group, *P* < 0.01) and Bax mRNA (1.84 ± 0.00 vs. 1.03 ± 0.02 of Sham group, *P* < 0.01) expression were found in the ischemic penumbra regions while the mRNA relative quantity of Bcl-2 (0.42 ± 0.01 vs. 1.06 ± 0.03 of Sham group, *P* < 0.01) was decreased significantly in MCAO group. Sul-F-H treatment could significantly suppress CHOP mRNA (1.42 ± 0.01 vs. 1.84 ± 0.01 of MCAO group, *P* < 0.01) and Bax mRNA (1.44 ± 0.01 vs 1.84 ± 0.00 of MCAO group, *P* < 0.01) expression and enhance the mRNA relative quantity of Bcl-2 (0.72 ± 0.01 vs. 0.42 ± 0.01 of MCAO group, *P* < 0.05) (Fig. 3E). These data suggested that Sul-F protected against MCAO-induced neuron injury via inhibition of cell apoptosis; moreover, inhibiting neuronal apoptosis induced by ER stress might be involved in the mechanism of the Sul-F protective effect on cerebral I/R injury.

### Sul-F protected against I/R injury induced by MCAO through suppressing apoptosis via the PERK and IRE1 signaling pathways

**Fig. 4:**
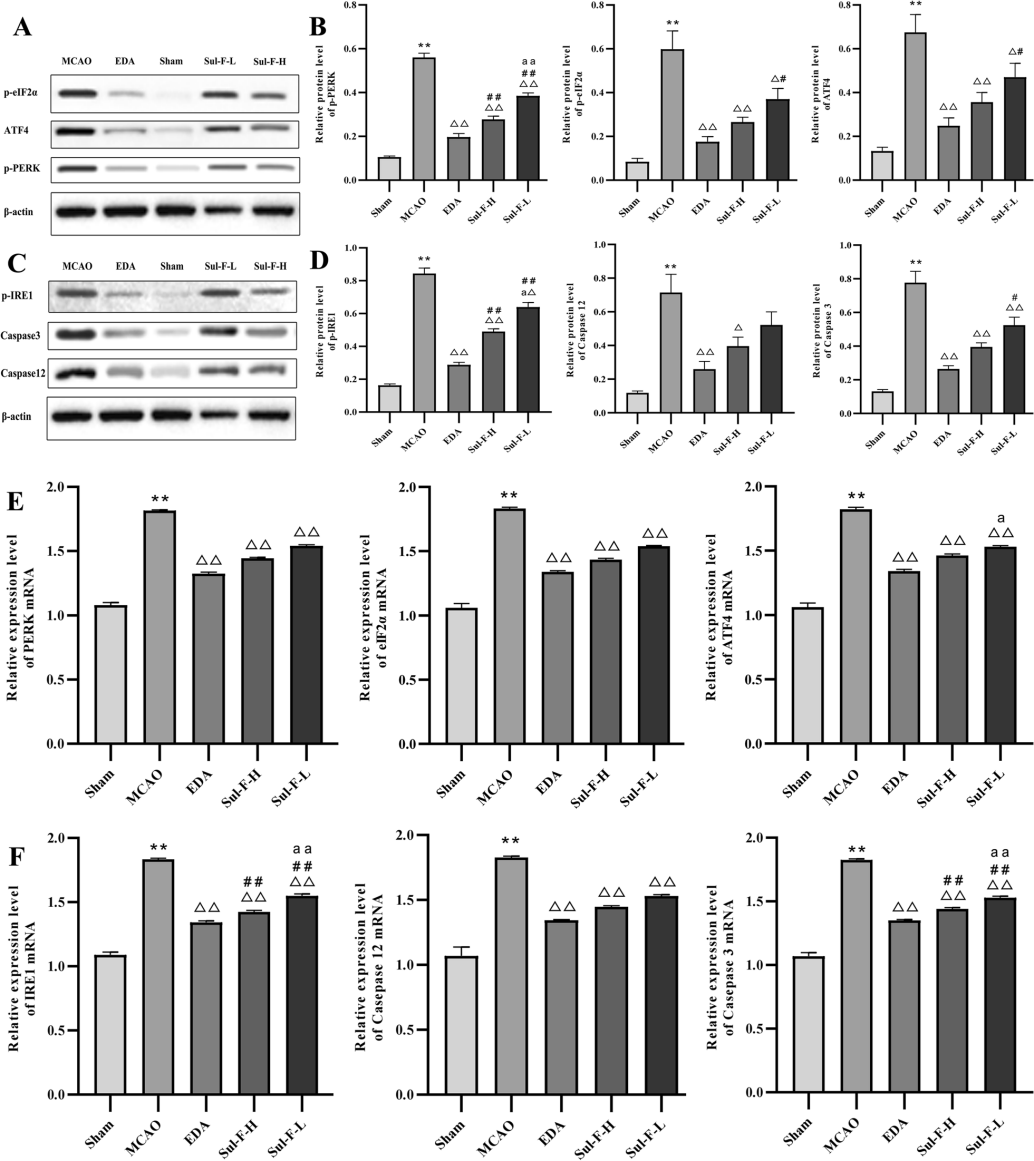
Sul-F treatment significantly attenuated the I/R-induced ER stress (n = 6). **A** At 24 h post-reperfusion, levels of p-PERK, p-eIF2α, ATF4 were evaluated with western blot. **B** The histogram showed quantification of images in bar diagram. The relative value of band gray was measured with Image J (1.49 V) and normalized to that of β-actin. **C** The mRNA levels of PERK, eIF2α and ATF4 were evaluated with qRT-PCR. **D** Representative western blot results of p-IRE1, Caspase12 and Caspase3 were shown. **E** The histogram showed quantification of images in bar diagram. The relative value of band gray was measured with Image J (1.49 V) and normalized to that of β-actin. **F** The mRNA levels of IRE1, Caspase12 and Caspase3 were evaluated with qRT-PCR. Data were expressed as the mean ± SEM from different assays. ^**^P < 0.01 compared with Sham group, ^△^*P* < 0.05 and ^△△^*P* < 0.01 compared with the MCAO group, ^#^*P* < 0.05 and ^##^*P* < 0.01 compared with the EDA group, ^a^*P* < 0.05 and ^aa^*P* < 0.01 compared with the Sul-F-H group

To further investigate whether ERS was involved in the effect of Sul-F on apoptosis in MCAO rats, the expression levels of ERS marker proteins including phosphorated-PERK (p-PERK), phosphorated-eIF2α (p-eIF2α), ATF4, phosphorated-IRE1 (p-IRE1), Caspase12 and Caspase3 were detected by Western blot. As shown in Fig. 4, compared with the Sham group, the relative protein expression level of p-PERK (0.56 ± 0.02 vs. 0.11 ± 0.00 of Sham group, *P* < 0.01), p-eIF2α (0.60 ± 0.08 vs. 0.08 ± 0.02 of Sham group, *P* < 0.01), ATF4 (0.68 ± 0.08 vs. 0.13 ± 0.02 of Sham group, *P* < 0.01), p-IRE1 (0.84 ± 0.03 vs. 0.16 ± 0.01 of Sham group, *P* < 0.01), Caspase12 (0.71 ± 0.11 vs. 0.12 ± 0.01 of Sham group, *P* < 0.01), and Caspase3 (0.78 ± 0.07 vs. 0.13 ± 0.01 of Sham group, *P* < 0.01) were significantly increased in the MCAO group. Sul-F-H treated group significantly decreased the expression levels of p-PERK (0.28 ± 0.01 vs. 0.56 ± 0.02 of MCAO group, *P* < 0.01), p-eIF2α (0.27 ± 0.02 vs. 0.60±0.08 of MCAO group, *P* < 0.01), ATF (0.36 ± 0.04 vs. 0.68 ± 0.08 of MCAO group, *P*＜0.01), p-IRE1 (0.49 ± 0.02 vs. 0.84 ± 0.03 of MCAO group, *P* < 0.01), Caspase12 (0.40 ± 0.05 vs. 0.71 ± 0.0.11 of MCAO group, *P* < 0.05) and Caspase3 (0.40 ± 0.02 vs. 0.78 ± 0.07 of MCAO group, *P* < 0.01).

Further investigation of qRT-PCR for ER stress marker genes revealed that there was a noticeable increase in PERK mRNA (1.82 ± 0.01 vs. 1.08 ± 0.02 of Sham group, *P* < 0.01), eIF2α mRNA (1.83 ± 0.01 vs. 1.06 ± 0.03 of Sham group, *P* < 0.01), ATF4 mRNA (1.82 ± 0.01 vs. 1.06 ± 0.03 of Sham group, *P* < 0.01), IRE1 mRNA (1.83 ± 0.01 vs. 1.09 ± 0.02 of Sham group, *P* < 0.01), Caspase12 mRNA (1.83 ± 0.01 vs. 1.07 ± 0.07 of Sham group, *P* < 0.01) and Caspase3 mRNA (1.83 ± 0.01 vs. 1.07 ± 0.03 of Sham group, *P* < 0.01) expression of the ischemic penumbra regions in MCAO group. Sul-F-H treatment could significantly suppress PERK mRNA (1.44 ± 0.01 vs. 1.82 ± 0.01 of MCAO group, *P* < 0.01), eIF2α mRNA (1.43 ± 0.01 vs. 1.83 ± 0.01 of MCAO group, *P* < 0.01), ATF4 mRNA (1.46 ± 0.01 vs. 1.82 ± 0.01 of MCAO group, *P* < 0.01), IRE1 mRNA (1.43 ± 0.01 vs. 1.83 ± 0.01 of MCAO group, *P* < 0.01), Caspase12 mRNA (1.45 ± 0.01 vs. 1.83 ± 0.01 of MCAO group, *P* < 0.01) and Caspase3 mRNA (1.44 ± 0.01 vs. 1.83 ± 0.01 of MCAO group, *P* < 0.01) expression (Fig. 4E, F). Together, the above data revealed that Sul-F exerted the protective effect in cerebral I/R injury through inhibited ER stress-induced apoptosis of neuron cells in ischemic penumbra.

## Discussion

Ischemic stroke causes an estimated 4.4 million deaths each year worldwide, placing a huge physical, emotional and financial burden on patients, families and national health service [37]. Current therapeutic options in stroke are still limited and brain injury caused by cerebral I/R remains a major challenge for the application of conventional management approaches. So there is an urgent need for a comprehensive strategy including neuroprotection and maximizing cerebral reperfusion rate to reduce reperfusion injury, and a comprehensive understanding of the pathophysiological process involved in cerebral I/R injury.

The neuronal damage in the ischemic region (penumbra) after cerebral I/R injury is slow and reversible [38, 39]. Therefore, the key of the clinical treatment is to save the ischemic penumbra of dying neurons and promote damage nerve function recovery. TCM has accumulated a wealth of experience in the treatment of stroke and modern pharmacology studies have shown that many Chinese herbal extracts can protect the neurological function from cerebral I/R injury by reducing penumbra apoptosis in a variety of ways [40–43]. Sul-F, a synthesized compound of formononetin, exerted beneficial effects in multiple cardiovascular and cerebrovascular diseases, including acute myocardial infarction [23] and stroke [24]. The aim of our study is to figure out the effect of Sul-F on cerebral I/R injury and to verify whether it works through suppressing ER stress-mediated apoptosis.

First of all, we want to figure out whether Sul-F has therapeutic effect on the neuron damage induced by cerebral I/R. To this end, we used MCAO model in rats established by wire embolization to mimic the alterations of cerebral I/R injury [44]. Consistent with previous studies [45, 46], our results indicated that I/R increased the numbers of TUNEL-positive cells and protein expression levels of CHOP and Bax after 24 h of cerebral I/R in the penumbra, which partially indicated that apoptosis was activated in the penumbra. Our results demonstrated that Sul-F alleviated neurological deficits evaluated by Zea-Longa, decreased infarct volume, and ameliorated pathological injury of brain tissue after 24 h of reperfusion. These data suggest that Sul-F could attenuate neuronal damage during cerebral I/R injury by inhibiting apoptosis in the penumbra area.

Inflammatory response plays an important role in cerebral I/R injury. Activation of microglia and astrocytes and exudation of leukocytes are key steps of inflammatory response in the central nervous system [47]. During cerebral I/R injury, the activated inflammatory cells synthesize and release inflammatory mediators, which in turn can further activate inflammatory cells, forming a vicious cycle and aggravating brain injury. IL-1 is secreted by activated astrocytes, oligodendrocytes and infiltrating macrophages after cerebral ischemia, which can promote the expression of adhesion molecules in endothelial cells, thereby aggravating local inflammatory response [48].The level of IL-1β in brain tissue of MCAO model rats began to increase at 6 h and reached the peak at 24 h, indicating that IL-1β was involved in the inflammatory response after cerebral I/R injury [49]. IL-6 plays a dual role in cerebral I/R injury. In the acute phase, IL-6 acts as an inflammatory mediator to promote brain injury, while in the subacute phase, it acts as a neurotrophic mediator to play a neuroprotective role [50]. TNF-α, mainly derives from activated glial cells, especially microglia, has complex biological activities, and its inhibitors can alleviate cerebral I/R injury [51].Previous researches demonstrated that a variety of TCM monomers could exert neuroprotective effects by reducing the levels of the inflammatory mediators TNF-α, IL-1β and IL-6 induced by brain ischemia reperfusion [52–54]. In addition, the inflammatory response is related to the endoplasmic reticulum stress signaling pathway, which participate together in the development of cerebral I/R injury [55, 56]. Meanwhile, our result showed that the levels of TNF-α, IL-1β, and IL-6 elevated accompanied with the activation of ERS signaling pathway in the MCAO rats. Interestingly, Sul-F treatment can significantly decrease the concentration of these inflammatory mediators and also inhibit the activation of the ER stress pathway, which suggested that the neuroprotective effects of Sul-F may be associated with the inhibition of neurogenic inflammation through suppressing ER stress pathway.

EDA, a free radical scavenger [57], is an efficacy drug in the therapy of cerebral infarction [58] and has been recommended for AIS treatment by Chinese and Japanese stroke care guidelines [59, 60]. EDA can scavenge many free radicals, such as hydroxyl (-OH), nitric oxide (NO) and peroxynitrite anion (ONOO-), and sequentially relieves cerebral oedema and inhibits delayed neuron death [61]. In addition, we also found that EDA could play a protective role on cerebral ischemia-reperfusion injury by inhibiting apoptosis mediated by ER stress signaling pathway, consistent with the previous study [62]. However, EDA is known to have a fairly short T1/2 and it should not be taken more than twice a day for those with impaired liver and kidney function based on its possible side effects [63]. Therefore, it is imperative to develop new pharmaceuticals. In preliminary study, it has been demonstrated that Sul-F at doses up to 2000 mg has no hepatorenal toxicity [64]. Additionally, we found that there was no difference between EDA (3 mg/kg) group and Sul-F-H (80 mg/kg) group in terms of therapeutic effect on cerebral ischemia–reperfusion injury, indicating the potential of Sul-F to be a clinical drug for the treatment of ischemic stroke.

ER stress is one of the main molecular events underlying the pathology of cerebral I/R injury [65]. Three major transmembrane proteins are involved in the ER stress-activated UPR: IRE1, PERK and ATF4. All these proteins are coupled with the GRP78 and stay inactive under physiological conditions. When the UPR is activated, after the GRP78 dissociation, the IRE1 and PERK oligomerize and phosphorylate to activate their downstream signals, and ATF4 is cleaved by the golgi and moves into the nucleus to act as a transcription promoter [66]. Subsequently, the activated PERK (p-PERK) promotes phosphorylation of eIF2α and activates selective translation of ATF4) [67]. ATF4 is an important mediator of UPR, which can promote cell survival by inducing amino acid metabolism, redox reaction, stress response and ER stress target genes of protein secretion. When cells are in stage of stress for a long time, ATF4 will activate the expression of its downstream target CHOP, a pro-apoptotic gene [68]. A previous study has indicated that CHOP gene transcription is one of the most critical pathways leading to apoptosis, and then, apoptosis can be regulated by regulating the expression of multiple anti-apoptotic and pro-apoptotic genes, such as Bcl-2 and Bax [66]. In addition, activation of IRE1 can promote the downstream Caspase12 signaling pathway to accelerate cell death [69]. Our present results indicated that Sul-F exerted its protection via suppression of Caspase12 signaling pathway.

**Fig. 5:**
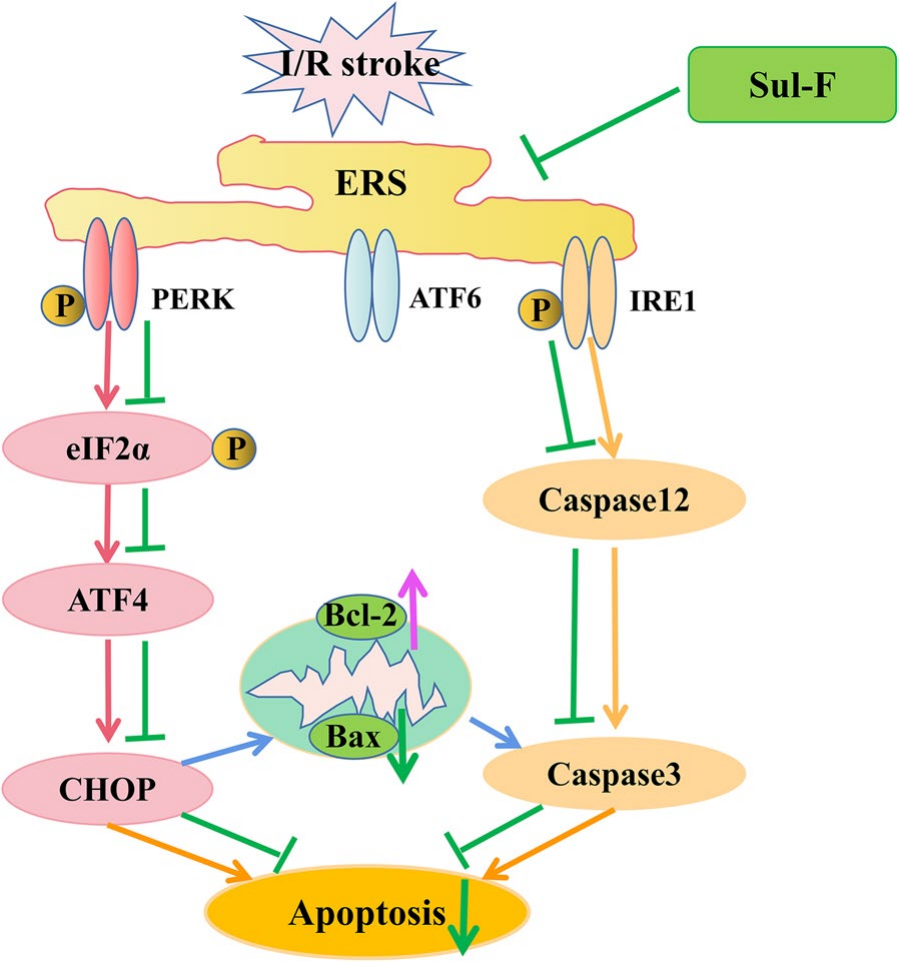
Schematic diagram of the molecular mechanisms underlying the protective effects of Sul-F treatment against cerebral I/R injury. Sul-F treatment alleviated the I/R-induced activation of ER stress, thereby inhibiting multiple downstream pathways, including PERK/eIF2α/ATF4, IRE1/Caspase12/Caspase3 and CHOP/Bcl-2/Bax signal pathway

Therefore, ER stress signaling pathway could be considered as the key molecular or signaling transduction pathway that modulates multiple targets in cerebral I/R injury. In the present study, we observed that I/R significantly activated ER stress evidenced by the increase in ATF4 as well as the hyper-phosphorylation of PERK and eIF2α, which was markedly reversed by Sul-F treatment. In conclusion, Sul-F treatment can rescue neurons against I/R injury through inhibiting PERK/eIF2α/ATF4 and IRE1/Caspase12/Caspase3 associated apoptosis pathways in the penumbra (Fig. 5).

In summary, Sul-F treatment attenuates cerebral I/R injury by inhibiting ER stress mediated apoptosis in ischemic penumbra through suppression of PERK/eIF2α/ATF4 and IRE1/Caspase12/Caspase3 signaling pathway. Our findings shed light on the novel therapeutic strategy of the administration of Sul-F in ischemic stroke.

## Supplementary Information

**Additional file 1: Table S1.** The list of primer sequence

**Additional file 2: **Real time PCR result

**Additional file 3: **Gel electrophoresis map

## Data Availability

The datasets supporting the conclusions of this article are included within the article and its additional files.

## Abbreviations

ER: Endoplasmic reticulum
I/R: Ischemia‐reperfusion
Sul-F: Sodium formononetin-3ʹ-sulphonate
BBB: Blood brain barrier
MCAO: Middle cerebral artery occlusion
BHD: Buyang Huanwu Decoction
UPR: Unfolded protein response
CCA: Common carotid artery
ECA: External carotid artery
ICA: Internal carotid artery
ATF4: Activating transcription factor 4
Bcl-2: B-cell lymphoma-2
Bax: Bcl-2-associated X protein
Caspase-3: Cysteine aspartic specific protease-3
Caspase-12: Cysteine aspartic specific protease-12
CHOP: C-EBP homologous protein
eIF2α: Eukaryotic initiation factor 2α
FMN: Formononetin
IL-1β: Interleukin-1β
IL-6: Interleukin-6
TNF-α: Tumor necrosis factor-α
GRP78: Glucose-regulated protein of 78 kDa
IRE1: Inositol-requiring enzyme 1
-OH: Hydroxyl
NO: Nitric oxide
ONOO-: Peroxynitrite anion
