# Supplementary material for: Sodium formononetin-3'-sulphonate alleviates cerebral ischemia–reperfusion injury in rats via suppressing endoplasmic reticulum stress-mediated apoptosis
Source: BMC Neurosci. 2022 Dec 9;23:74. doi: 10.1186/s12868-022-00762-4 (PMC9733209; doi:10.1186/s12868-022-00762-4)
Supplement: Supplementary file 1 — Additional file 1: Table S1. The list of primer sequence [file 12868_2022_762_MOESM1_ESM.doc]

Additional file Table.S1 The list of primer sequence

| gene |  | primer sequence |
| --- | --- | --- |
| β-actin | Forward(5’-3’) | TGTCACCAACTGGGACGATA |
|  | Reverse(5’-3’) | GGGGTGTTGAAGGTCTCAAA |
| Bcl-2 | Forward(5’-3’) | CCCCATCCCTGAAGAGTTCCT |
|  | Reverse(5’-3’) | GCCACCTACCTGAATGACCAC |
| Bax | Forward(5’-3’) | ATTGGCGATGAACTGGACAACA |
|  | Reverse(5’-3’) | CCCAGTTGAAGTTGCCGTCT |
| Caspase-3 | Forward(5’-3’) | TGTGGACCTGAAAAAAC |
|  | Reverse(5’-3’) | GCCTGAATGATGAAGAG |
| Caspase-12 | Forward(5’-3’) | ATAGCCACTGCTGATACAGA |
|  | Reverse(5’-3’) | CCACTCTTGCCTACCTTCC |
| CHOP | Forward(5’-3’) | AGCCTGGTATGAGGATCTGC |
|  | Reverse(5’-3’) | GACTGGAATCTGGAGAGCGA |
| PERK | Forward(5’-3’) | CCTTCCAGCAATCACAGCTC |
|  | Reverse(5’-3’) | TGGTTTCCAGTAGCTTCCGT |
| eIF2α | Forward(5’-3’) | TTATGCCTGCGAAAGCAAC |
|  | Reverse(5’-3’) | TTCCATTTGTCCTCGAAGGT |
| IRE1 | Forward(5’-3’) | AGAGCCCATCACCTTGCTT |
|  | Reverse(5’-3’) | TGATCCTGCCATGTGCGTT |
| ATF4 | Forward(5’-3’) | GCCAAGCACTTCAAACCTCA |
|  | Reverse(5’-3’) | GCATGGTTTCCAGGTCATCC |
