# Supplementary material for: Sodium formononetin-3'-sulphonate alleviates cerebral ischemia–reperfusion injury in rats via suppressing endoplasmic reticulum stress-mediated apoptosis
Source: BMC Neurosci. 2022 Dec 9;23:74. doi: 10.1186/s12868-022-00762-4 (PMC9733209; doi:10.1186/s12868-022-00762-4)
Supplement: Supplementary file 2 — Additional file 2: Real time PCR result [file 12868_2022_762_MOESM2_ESM.pdf]

Real time PCR result

1. Amplification curve and melt curve

β-actin

Amplification curve

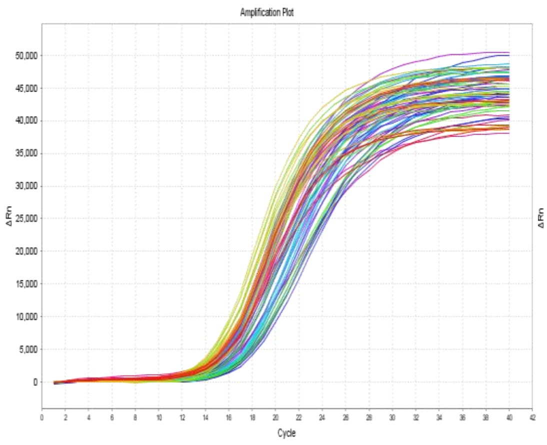

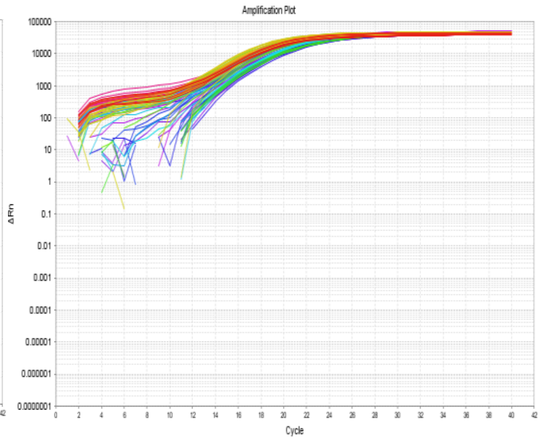

Melt curve

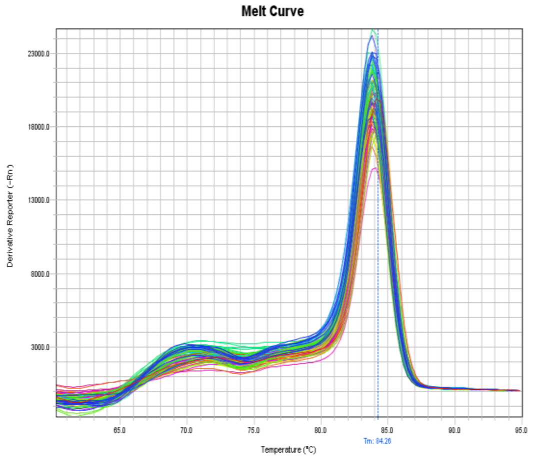

Bcl-2

Amplification curve

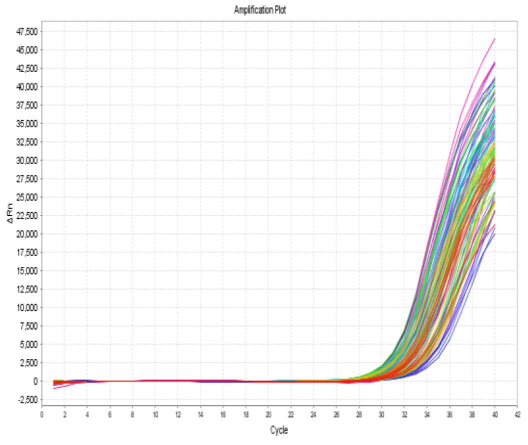

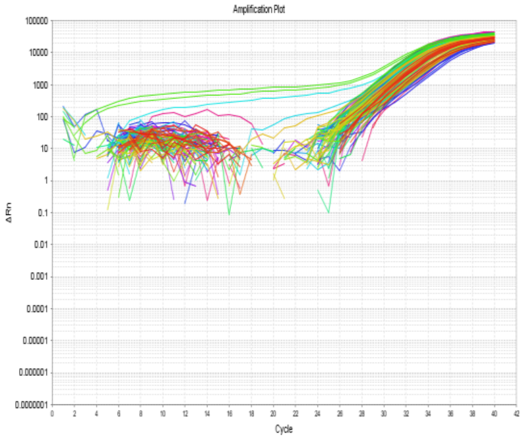

Melt curve

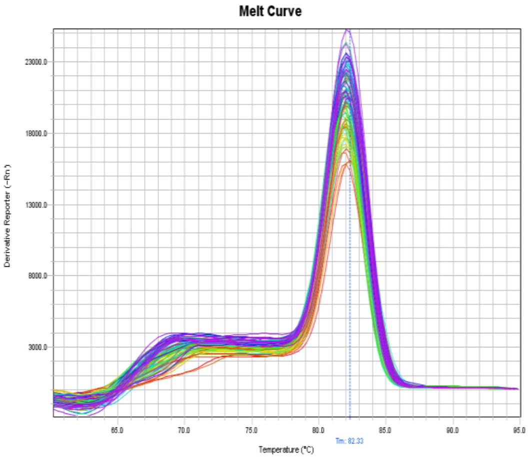

Bax

Amplification curve

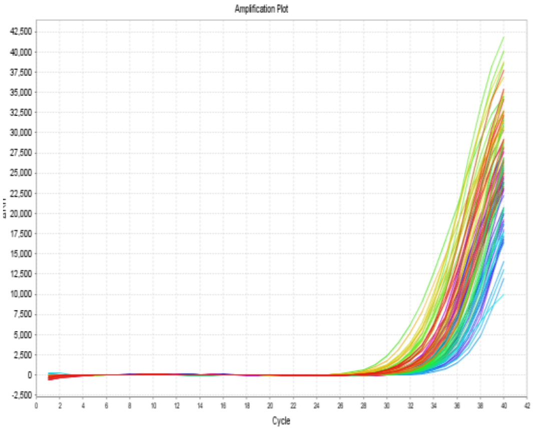

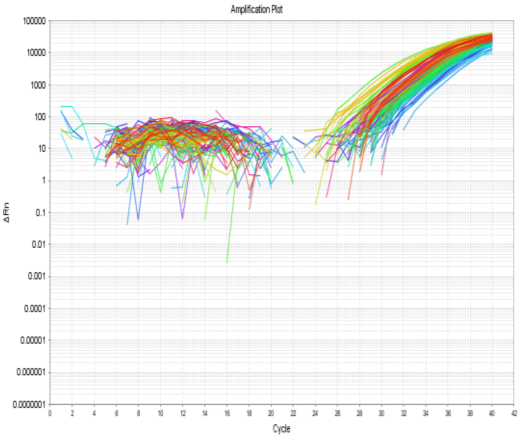

Melt curve

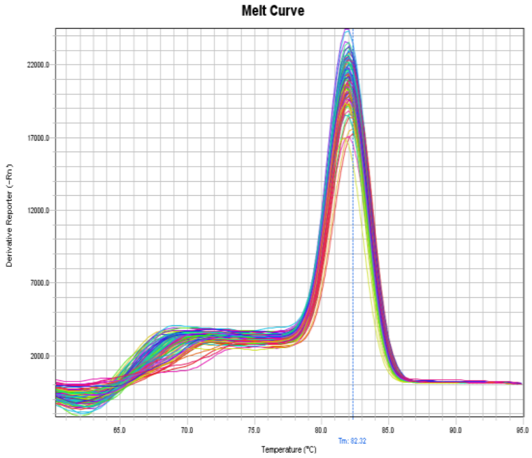

Caspase-3

Amplification curve

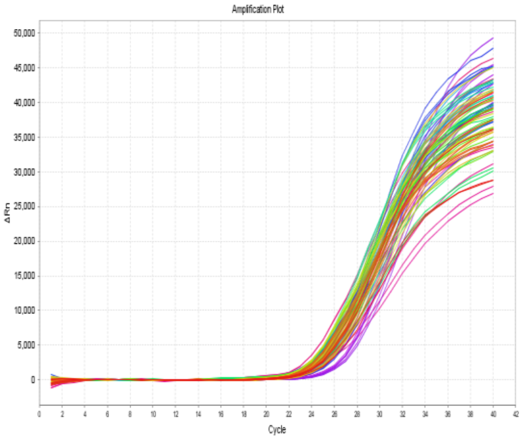

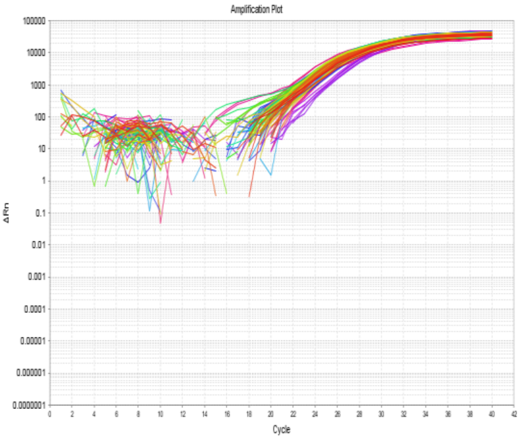

Melt curve

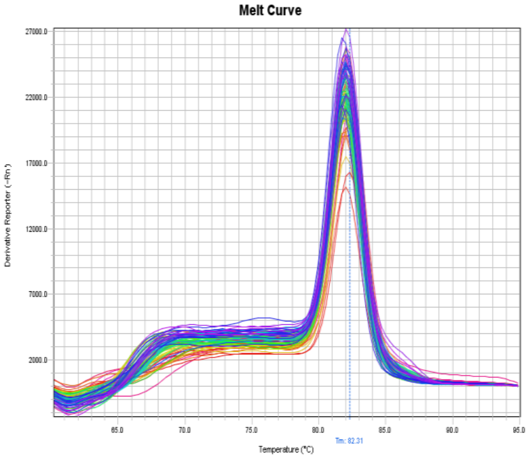

Caspase-12

Amplification curve

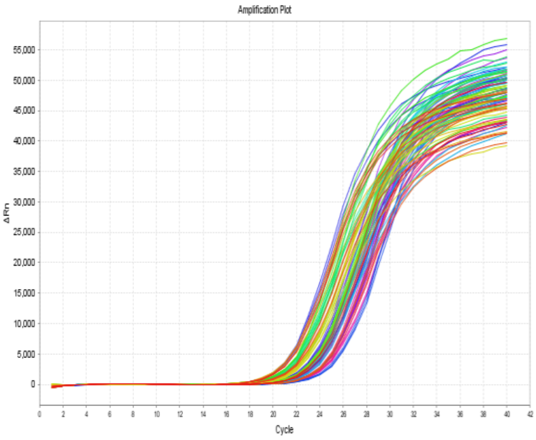

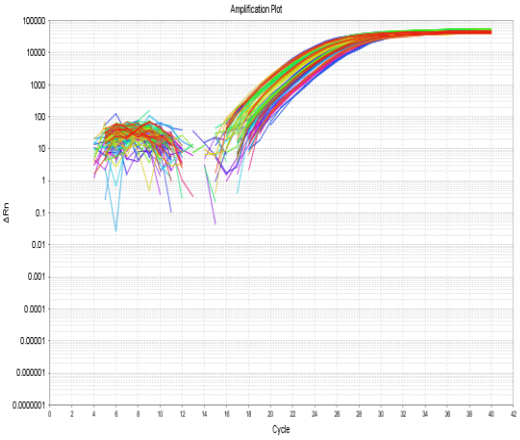

Melt curve

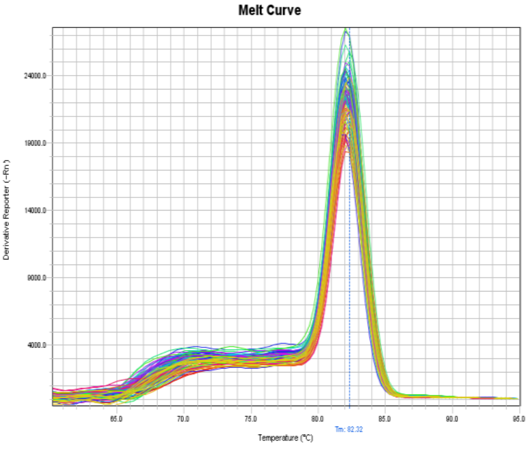

CHOP

Amplification curve

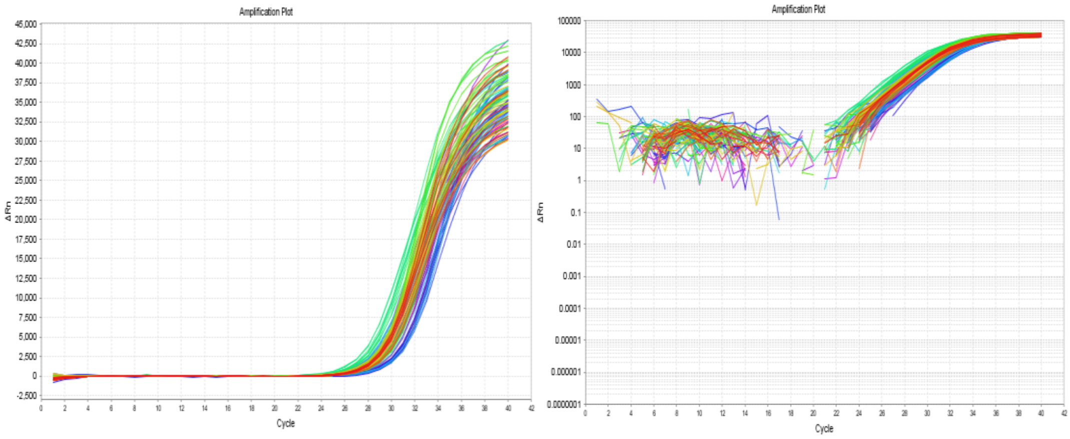

Melt curve

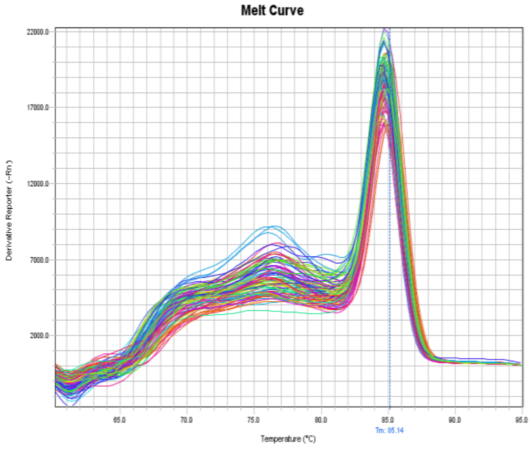

PERK

Amplification curve

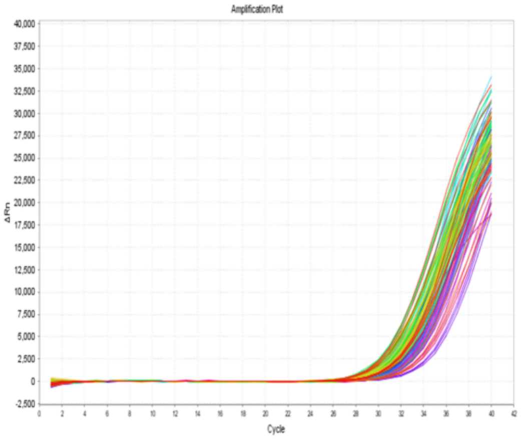

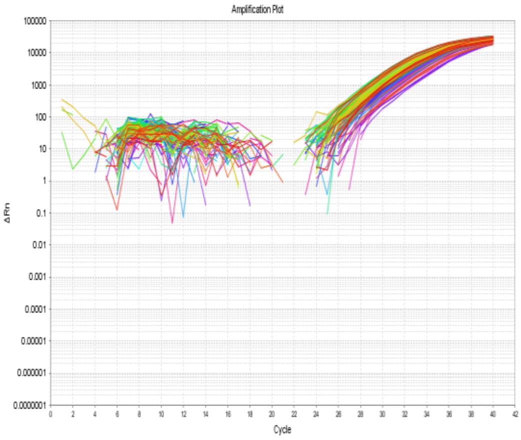

Melt curve

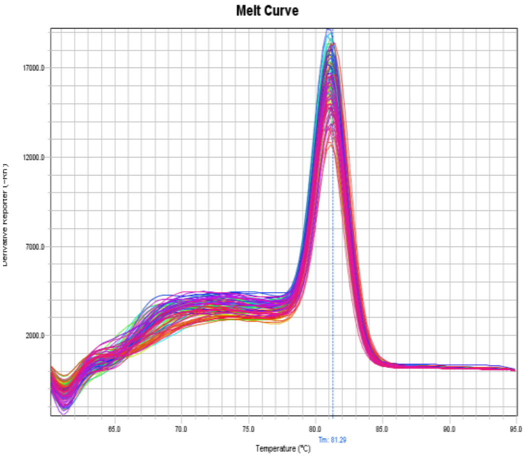

eIF2α

Amplification curve

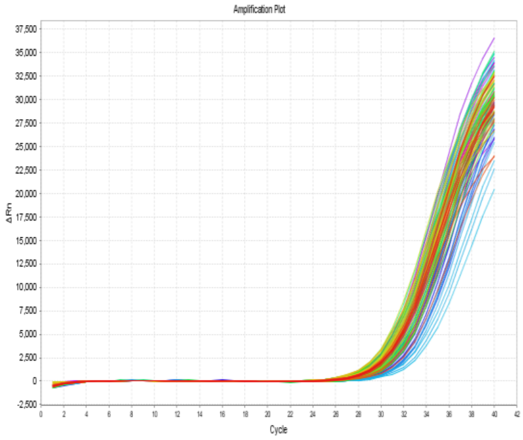

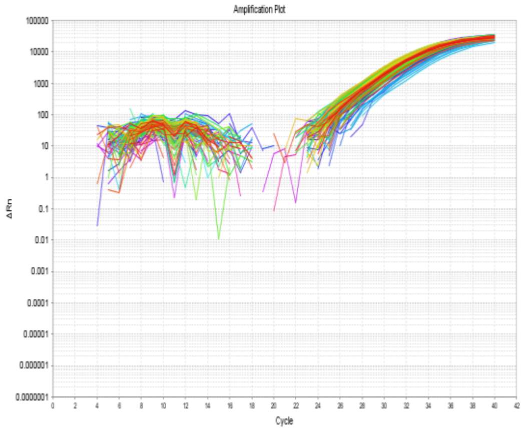

Melt curve

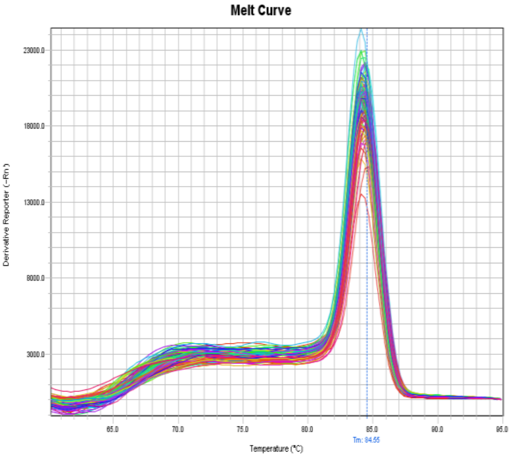

IRE1

Amplification curve

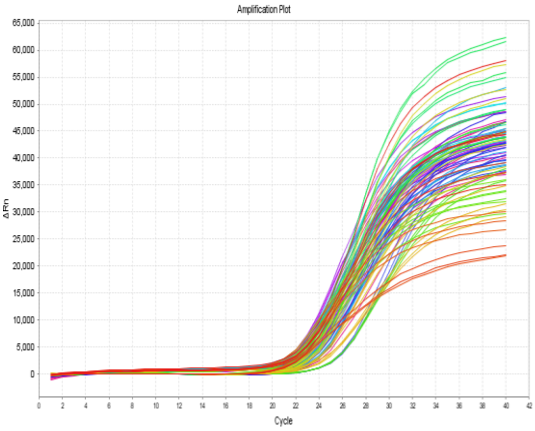

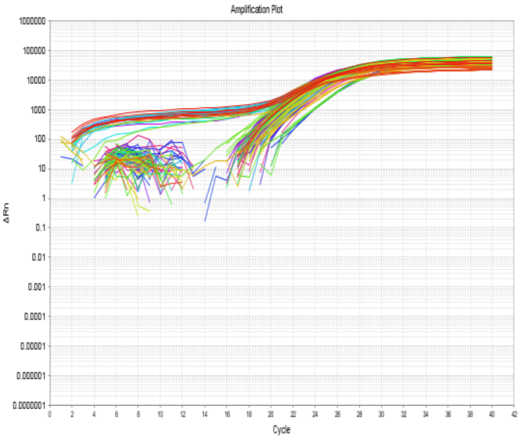

Melt curve

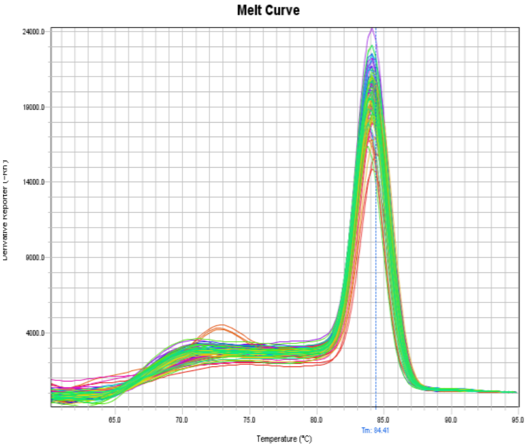

ATF4

Amplification curve

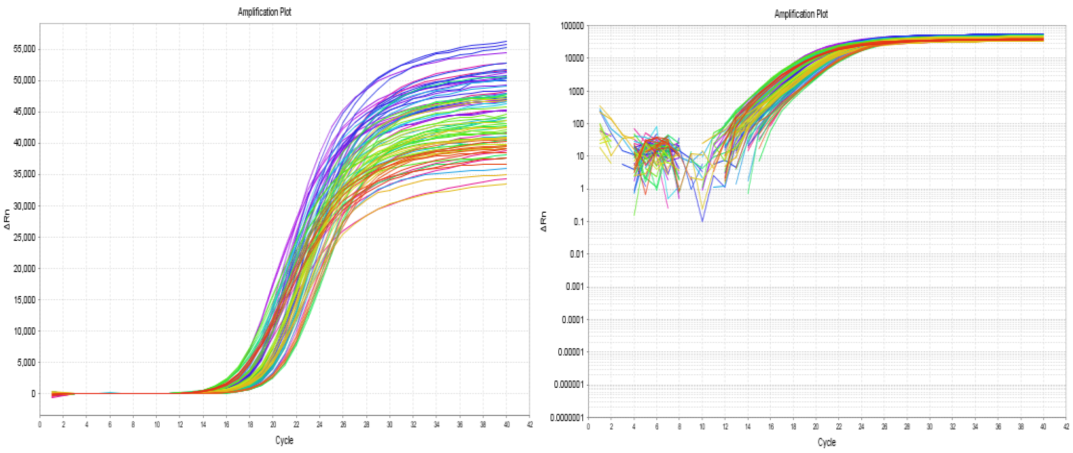

Melt curve

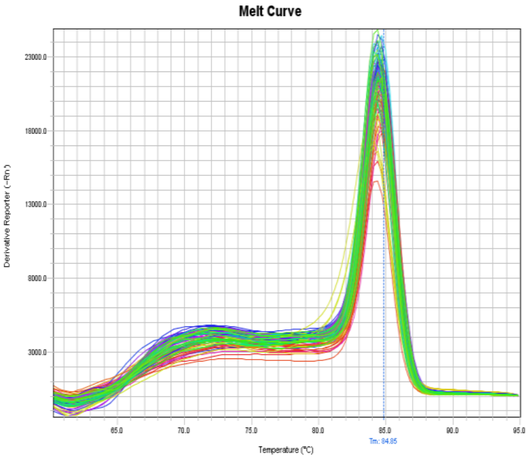

2. Data analysis

Calculation formula of relative expression：

$RQ=2^{-(\triangle Ct_q-\triangle Ct_{cb})}$

$\triangle Ct_q$=Average CT value of target gene in the tested group- that of internal reference in the tested group

$\triangle Ct_{cb}$=Average CT value of target gene in the control group- that of internal reference in the control group

Sample Name	Target Name	CT	Average CT value
1-1	Actin	17.00252279	17.0298576
1-1	Actin	17.01252496	
1-1	Actin	17.07452505	
1-2	Actin	15.63752303	15.58652436
1-2	Actin	15.62852465	
1-2	Actin	15.4935254	
2-1	Actin	15.36852471	15.47319067
2-1	Actin	15.36852406	
2-1	Actin	15.68252323	
2-2	Actin	16.20952297	16.16052321
2-2	Actin	16.18152337	
2-2	Actin	16.09052329	

2-3	Actin	15.21452256	15.19919014
2-3	Actin	15.01952252	
2-3	Actin	15.36352534	
2-4	Actin	15.12852344	15.03552333
2-4	Actin	14.99952358	
2-4	Actin	14.97852297	
2-5	Actin	16.04952404	16.09219046
2-5	Actin	16.15652364	
2-5	Actin	16.0705237	
2-6	Actin	15.66452332	15.60218973
2-6	Actin	15.62152303	
2-6	Actin	15.52052283	
3-1	Actin	16.46152499	16.38919075
3-1	Actin	16.33452415	
3-1	Actin	16.3715231	
3-2	Actin	14.03752271	14.29085624
3-2	Actin	14.34852259	
3-2	Actin	14.48652343	
3-3	Actin	16.5605243	16.66752465
3-3	Actin	16.51352507	
3-3	Actin	16.92852458	
3-4	Actin	16.10552426	15.95285735
3-4	Actin	15.97052321	
3-4	Actin	15.78252459	
3-5	Actin	16.95252321	16.72352385
3-5	Actin	16.6375249	
3-5	Actin	16.58052345	
3-6	Actin	16.27252442	16.19613357
3-6	Actin	16.27835248	
3-6	Actin	16.03752382	
4-1	Actin	17.27452462	17.11685739
4-1	Actin	17.01552503	
4-1	Actin	17.06052253	
4-2	Actin	16.477524	16.50252398
4-2	Actin	16.50752536	
4-2	Actin	16.52252256	
4-3	Actin	15.51252299	15.5588572
4-3	Actin	15.53252469	
4-3	Actin	15.63152391	
4-4	Actin	17.481524	17.40385715
4-4	Actin	17.44152317	
4-4	Actin	17.28852428	
4-5	Actin	15.8045246	15.6598568
4-5	Actin	15.56752254	
4-5	Actin	15.60752324	
4-6	Actin	15.48052358	15.4335246

4-6	Actin	15.51952535	
4-6	Actin	15.30052487	
5-1	Actin	16.21252531	16.0991913
5-1	Actin	16.29352518	
5-1	Actin	15.79152341	
5-2	Actin	16.22652341	16.14419047
5-2	Actin	16.11052297	
5-2	Actin	16.09552501	
5-3	Actin	16.96352307	16.9361907
5-3	Actin	16.81052498	
5-3	Actin	17.03452406	
5-4	Actin	16.54252397	16.3958571
5-4	Actin	16.20252306	
5-4	Actin	16.44252427	
5-5	Actin	17.05952282	17.22919065
5-5	Actin	17.45952432	
5-5	Actin	17.16852481	
5-6	Actin	17.17452363	17.20019013
5-6	Actin	17.36452331	
5-6	Actin	17.06152345	
6-1	Actin	15.8315234	15.7738576
6-1	Actin	15.75152532	
6-1	Actin	15.73852407	
6-2	Actin	16.53152354	16.30852355
6-2	Actin	16.23252278	
6-2	Actin	16.16152434	
6-3	Actin	16.16652325	16.06952362
6-3	Actin	15.93852347	
6-3	Actin	16.10352415	
6-4	Actin	15.5135231	15.5225231
6-4	Actin	15.66152254	
6-4	Actin	15.39252368	
6-5	Actin	15.42652272	15.43919081
6-5	Actin	15.32352431	
6-5	Actin	15.56752539	
6-6	Actin	16.94952271	16.75085754
6-6	Actin	16.85452477	
6-6	Actin	16.44852514	

Sample Name	Target Name	CT	Average CT value
1-1	Bcl-2	34.19552481	34.38819086
1-1	Bcl-2	34.3805233	
1-1	Bcl-2	34.58852448	
1-2	Bcl-2	33.11852501	32.95152436
1-2	Bcl-2	32.76752348	
1-2	Bcl-2	32.96852459	

2-1	Bcl-2	34.16952533	34.08319176
2-1	Bcl-2	34.25852506	
2-1	Bcl-2	33.8215249	
2-2	Bcl-2	34.50652477	34.64285779
2-2	Bcl-2	34.43052446	
2-2	Bcl-2	34.99152413	
2-3	Bcl-2	33.93152351	33.87819024
2-3	Bcl-2	33.76052353	
2-3	Bcl-2	33.94252369	
2-4	Bcl-2	33.63652407	33.77785734
2-4	Bcl-2	33.75752338	
2-4	Bcl-2	33.93952457	
2-5	Bcl-2	34.61652347	34.64652353
2-5	Bcl-2	34.70952256	
2-5	Bcl-2	34.61352456	
2-6	Bcl-2	34.45152381	34.26885782
2-6	Bcl-2	34.22452497	
2-6	Bcl-2	34.13052467	
3-1	Bcl-2	33.85652428	34.21885719
3-1	Bcl-2	33.96452359	
3-1	Bcl-2	34.8355237	
3-2	Bcl-2	32.19952441	32.17385756
3-2	Bcl-2	32.06252448	
3-2	Bcl-2	32.25952378	
3-3	Bcl-2	34.74752497	34.50385822
3-3	Bcl-2	34.39152469	
3-3	Bcl-2	34.37252499	
3-4	Bcl-2	33.81452266	33.81885648
3-4	Bcl-2	33.76152381	
3-4	Bcl-2	33.88052296	
3-5	Bcl-2	34.3765227	34.46652364
3-5	Bcl-2	34.37852528	
3-5	Bcl-2	34.64452293	
3-6	Bcl-2	34.29152342	33.98685737
3-6	Bcl-2	33.95052337	
3-6	Bcl-2	33.71852533	
4-1	Bcl-2	34.37352273	34.42652312
4-1	Bcl-2	34.43052381	
4-1	Bcl-2	34.47552283	
4-2	Bcl-2	32.96252402	33.60919059
4-2	Bcl-2	33.90752332	
4-2	Bcl-2	33.95752445	
4-3	Bcl-2	32.62252317	32.73818983
4-3	Bcl-2	32.96852319	
4-3	Bcl-2	32.62352314	
4-4	Bcl-2	34.92752353	34.75219039

4-4	Bcl-2	34.51852479	
4-4	Bcl-2	34.81052286	
4-5	Bcl-2	33.13752391	33.00352364
4-5	Bcl-2	32.79952395	
4-5	Bcl-2	33.07352307	
4-6	Bcl-2	32.85052303	32.80485738
4-6	Bcl-2	32.60252412	
4-6	Bcl-2	32.961525	
5-1	Bcl-2	34.03952509	34.14052404
5-1	Bcl-2	34.23352404	
5-1	Bcl-2	34.14852299	
5-2	Bcl-2	34.19252434	34.22285751
5-2	Bcl-2	34.2325228	
5-2	Bcl-2	34.24352539	
5-3	Bcl-2	34.94352519	34.90652377
5-3	Bcl-2	34.8675233	
5-3	Bcl-2	34.90852281	
5-4	Bcl-2	34.33152358	34.42552365
5-4	Bcl-2	34.61952442	
5-4	Bcl-2	34.32552297	
5-5	Bcl-2	35.3105247	35.32652378
5-5	Bcl-2	35.43752311	
5-5	Bcl-2	35.23152353	
5-6	Bcl-2	35.27952416	35.25752421
5-6	Bcl-2	35.32452499	
5-6	Bcl-2	35.1685235	
6-1	Bcl-2	33.66152518	33.65152435
6-1	Bcl-2	33.56252468	
6-1	Bcl-2	33.73052319	
6-2	Bcl-2	34.21152447	34.04652402
6-2	Bcl-2	33.98252354	
6-2	Bcl-2	33.94552403	
6-3	Bcl-2	33.9275235	33.93152401
6-3	Bcl-2	33.96052511	
6-3	Bcl-2	33.90652342	
6-4	Bcl-2	33.27352445	33.3931907
6-4	Bcl-2	33.6165246	
6-4	Bcl-2	33.28952303	
6-5	Bcl-2	33.26852411	33.28952408
6-5	Bcl-2	33.16552416	
6-5	Bcl-2	33.43452398	
6-6	Bcl-2	34.49315251	34.57973298
6-6	Bcl-2	34.72252264	
6-6	Bcl-2	34.52352379	
Sample Name	Target Name	CT	Average CT value

1-1	Bax	32.74952292	32.85019028
1-1	Bax	32.83252344	
1-1	Bax	32.96852447	
1-2	Bax	31.26335243	31.34546714
1-2	Bax	31.49252527	
1-2	Bax	31.28052371	
2-1	Bax	30.22452338	30.40085679
2-1	Bax	30.48252445	
2-1	Bax	30.49552254	
2-2	Bax	31.12852453	31.11019104
2-2	Bax	31.07552523	
2-2	Bax	31.12652336	
2-3	Bax	30.16952385	30.14452367
2-3	Bax	30.01052359	
2-3	Bax	30.25352358	
2-4	Bax	30.09152353	29.97985694
2-4	Bax	29.84552455	
2-4	Bax	30.00252273	
2-5	Bax	31.04452276	31.04185638
2-5	Bax	31.1845234	
2-5	Bax	30.89652298	
2-6	Bax	30.55052383	30.54719046
2-6	Bax	30.57052389	
2-6	Bax	30.52052367	
3-1	Bax	31.74852409	31.75819029
3-1	Bax	31.563523	
3-1	Bax	31.96252378	
3-2	Bax	29.95752329	29.69519013
3-2	Bax	29.69552255	
3-2	Bax	29.43252454	
3-3	Bax	32.00852328	32.05318968
3-3	Bax	32.07652281	
3-3	Bax	32.07452296	
3-4	Bax	31.04752359	31.31252392
3-4	Bax	31.38552426	
3-4	Bax	31.50452391	
3-5	Bax	31.97952276	32.07519047
3-5	Bax	32.19052349	
3-5	Bax	32.05552515	
3-6	Bax	31.3285242	31.5821909
3-6	Bax	31.68352428	
3-6	Bax	31.73452422	
4-1	Bax	32.56825245	32.82010009
4-1	Bax	32.92752357	
4-1	Bax	32.96452424	
4-2	Bax	32.00352306	32.28985767

4-2	Bax	32.49552524	
4-2	Bax	32.37052471	
4-3	Bax	31.2585232	31.32219078
4-3	Bax	31.44652378	
4-3	Bax	31.26152537	
4-4	Bax	33.41152496	33.25819159
4-4	Bax	33.16652493	
4-4	Bax	33.19652489	
4-5	Bax	31.67052537	31.46252436
4-5	Bax	31.40652505	
4-5	Bax	31.31052267	
4-6	Bax	31.40552486	31.15652367
4-6	Bax	31.04552288	
4-6	Bax	31.01852326	
5-1	Bax	31.14952514	31.28319146
5-1	Bax	31.36652462	
5-1	Bax	31.33352462	
5-2	Bax	31.41352468	31.33849986
5-2	Bax	31.43745234	
5-2	Bax	31.16452257	
5-3	Bax	32.14752534	32.09719061
5-3	Bax	32.1555239	
5-3	Bax	31.98852259	
5-4	Bax	31.5595254	31.61052391
5-4	Bax	31.68652254	
5-4	Bax	31.5855238	
5-5	Bax	32.3045245	32.39485751
5-5	Bax	32.3765251	
5-5	Bax	32.50352293	
5-6	Bax	32.46552323	32.37085675
5-6	Bax	32.14552331	
5-6	Bax	32.50152371	
6-1	Bax	31.03852458	31.08119113
6-1	Bax	31.27852516	
6-1	Bax	30.92652364	
6-2	Bax	31.63852276	31.60252284
6-2	Bax	31.37452269	
6-2	Bax	31.79452308	
6-3	Bax	31.30852347	31.37119082
6-3	Bax	31.54952542	
6-3	Bax	31.25552357	
6-4	Bax	30.84352484	30.84185794
6-4	Bax	30.80452466	
6-4	Bax	30.87752433	
6-5	Bax	30.64352376	30.71185708
6-5	Bax	30.82052365	

6-5	Bax	30.67152382	
6-6	Bax	31.7515234	32.04252439
6-6	Bax	32.12352464	
6-6	Bax	32.25252513	

Sample Name	Target Name	CT	Average CT value
1-1	Caspase-3	26.91952286	27.02219014
1-1	Caspase-3	27.26952427	
1-1	Caspase-3	26.87752329	
1-2	Caspase-3	25.36523899	25.44842937
1-2	Caspase-3	25.45652494	
1-2	Caspase-3	25.52352419	
2-1	Caspase-3	24.41652424	24.61218982
2-1	Caspase-3	24.71552257	
2-1	Caspase-3	24.70452264	
2-2	Caspase-3	25.26552469	25.28085737
2-2	Caspase-3	25.16952468	
2-2	Caspase-3	25.40752276	
2-3	Caspase-3	24.31252275	24.31785735
2-3	Caspase-3	24.3495242	
2-3	Caspase-3	24.29152511	
2-4	Caspase-3	24.12352454	24.1525241
2-4	Caspase-3	24.08252458	
2-4	Caspase-3	24.25152317	
2-5	Caspase-3	25.16552363	25.23985653
2-5	Caspase-3	25.44952328	
2-5	Caspase-3	25.10452267	
2-6	Caspase-3	24.86552304	24.70418956
2-6	Caspase-3	24.7545226	
2-6	Caspase-3	24.49252303	
3-1	Caspase-3	25.82752385	25.94519058
3-1	Caspase-3	26.01452517	
3-1	Caspase-3	25.99352273	
3-2	Caspase-3	23.86552433	23.82885674
3-2	Caspase-3	23.9335226	
3-2	Caspase-3	23.68752329	
3-3	Caspase-3	26.37552436	26.20852334
3-3	Caspase-3	26.059523	
3-3	Caspase-3	26.19052266	
3-4	Caspase-3	25.452523	25.5251904
3-4	Caspase-3	25.56552393	
3-4	Caspase-3	25.55752429	
3-5	Caspase-3	26.29052413	26.30452414
3-5	Caspase-3	26.29852401	
3-5	Caspase-3	26.32452427	
3-6	Caspase-3	25.77652476	25.76380006

3-6	Caspase-3	25.9453523	
3-6	Caspase-3	25.56952314	
4-1	Caspase-3	27.28352308	27.13176018
4-1	Caspase-3	27.06652384	
4-1	Caspase-3	27.04523362	
4-2	Caspase-3	26.64052372	26.46585734
4-2	Caspase-3	26.1855236	
4-2	Caspase-3	26.57152471	
4-3	Caspase-3	25.38552378	25.33585688
4-3	Caspase-3	25.45052366	
4-3	Caspase-3	25.17152319	
4-4	Caspase-3	27.16252465	27.21052395
4-4	Caspase-3	27.25952354	
4-4	Caspase-3	27.20952366	
4-5	Caspase-3	25.42152391	25.55452395
4-5	Caspase-3	25.34352517	
4-5	Caspase-3	25.89852277	
4-6	Caspase-3	25.4885241	25.36552424
4-6	Caspase-3	25.24352486	
4-6	Caspase-3	25.36452375	
5-1	Caspase-3	25.54352314	25.44719014
5-1	Caspase-3	25.21952437	
5-1	Caspase-3	25.57852291	
5-2	Caspase-3	25.40952297	25.53919066
5-2	Caspase-3	25.55852492	
5-2	Caspase-3	25.64952408	
5-3	Caspase-3	26.21152431	26.34119129
5-3	Caspase-3	26.58952483	
5-3	Caspase-3	26.22252473	
5-4	Caspase-3	25.63152475	25.79819104
5-4	Caspase-3	25.88352422	
5-4	Caspase-3	25.87952415	
5-5	Caspase-3	26.62052428	26.61843197
5-5	Caspase-3	26.45524655	
5-5	Caspase-3	26.77952507	
5-6	Caspase-3	26.69852478	26.54352424
5-6	Caspase-3	26.50752273	
5-6	Caspase-3	26.4245252	
6-1	Caspase-3	25.34652337	25.23119056
6-1	Caspase-3	25.41052326	
6-1	Caspase-3	24.93652505	
6-2	Caspase-3	25.78452402	25.80252394
6-2	Caspase-3	25.95552494	
6-2	Caspase-3	25.66752287	
6-3	Caspase-3	25.79752529	25.54185764
6-3	Caspase-3	25.36852398	

6-3	Caspase-3	25.45952366	
6-4	Caspase-3	24.99652441	24.96152408
6-4	Caspase-3	24.9535247	
6-4	Caspase-3	24.93452312	
6-5	Caspase-3	24.79952519	24.88419096
6-5	Caspase-3	24.87752481	
6-5	Caspase-3	24.97552288	
6-6	Caspase-3	26.42052312	26.23385658
6-6	Caspase-3	26.28452399	
6-6	Caspase-3	25.99652262	

Sample Name	Target Name	CT	Average CT value
1-1	Caspase-12	24.54052503	24.37152412
1-1	Caspase-12	24.24852403	
1-1	Caspase-12	24.32552329	
1-2	Caspase-12	22.05752423	22.68219141
1-2	Caspase-12	22.03752521	
1-2	Caspase-12	23.95152478	
2-1	Caspase-12	21.42352407	21.92752405
2-1	Caspase-12	21.36052508	
2-1	Caspase-12	22.99852298	
2-2	Caspase-12	22.44252302	22.64990023
2-2	Caspase-12	22.87865238	
2-2	Caspase-12	22.62852528	
2-3	Caspase-12	21.74552314	21.67652422
2-3	Caspase-12	21.40852439	
2-3	Caspase-12	21.87552511	
2-4	Caspase-12	21.50752374	21.51219036
2-4	Caspase-12	21.40652371	
2-4	Caspase-12	21.62252363	
2-5	Caspase-12	22.34652338	22.53519003
2-5	Caspase-12	22.75652298	
2-5	Caspase-12	22.50252374	
2-6	Caspase-12	22.02552371	22.09085706
2-6	Caspase-12	22.13152404	
2-6	Caspase-12	22.11552342	
3-1	Caspase-12	23.38452293	23.30418986
3-1	Caspase-12	23.3955237	
3-1	Caspase-12	23.13252295	
3-2	Caspase-12	21.07952466	21.19052487
3-2	Caspase-12	21.30452527	
3-2	Caspase-12	21.18752468	
3-3	Caspase-12	23.40952295	23.59352329
3-3	Caspase-12	23.6675238	
3-3	Caspase-12	23.70352311	
3-4	Caspase-12	22.94852436	22.8925245

3-4	Caspase-12	22.95552505	
3-4	Caspase-12	22.77352409	
3-5	Caspase-12	23.46552257	23.63218964
3-5	Caspase-12	23.72852301	
3-5	Caspase-12	23.70252335	
3-6	Caspase-12	23.09652301	23.10252305
3-6	Caspase-12	23.03652332	
3-6	Caspase-12	23.17452281	
4-1	Caspase-12	24.67952325	24.5361906
4-1	Caspase-12	24.46452475	
4-1	Caspase-12	24.4645238	
4-2	Caspase-12	23.57352319	23.6931901
4-2	Caspase-12	23.90352445	
4-2	Caspase-12	23.60252266	
4-3	Caspase-12	22.85052319	22.95052429
4-3	Caspase-12	22.94652482	
4-3	Caspase-12	23.05452485	
4-4	Caspase-12	24.25052417	24.26485738
4-4	Caspase-12	24.29552283	
4-4	Caspase-12	24.24852514	
4-5	Caspase-12	23.07852285	23.03652366
4-5	Caspase-12	23.01852378	
4-5	Caspase-12	23.01252436	
4-6	Caspase-12	22.95052382	22.75300015
4-6	Caspase-12	22.74695244	
4-6	Caspase-12	22.56152419	
5-1	Caspase-12	23.00252277	22.84852331
5-1	Caspase-12	22.83652268	
5-1	Caspase-12	22.70652448	
5-2	Caspase-12	22.84252389	22.89585738
5-2	Caspase-12	22.7595248	
5-2	Caspase-12	23.08552344	
5-3	Caspase-12	23.53652542	23.62985803
5-3	Caspase-12	23.53252329	
5-3	Caspase-12	23.82052538	
5-4	Caspase-12	23.16752325	23.13285668
5-4	Caspase-12	23.28552268	
5-4	Caspase-12	22.94552412	
5-5	Caspase-12	24.02052434	23.9408577
5-5	Caspase-12	23.83052445	
5-5	Caspase-12	23.97152432	
5-6	Caspase-12	23.79052442	23.92119061
5-6	Caspase-12	23.93952443	
5-6	Caspase-12	24.03352299	
6-1	Caspase-12	22.48152342	22.61752379
6-1	Caspase-12	22.58852291	

6-1	Caspase-12	22.78252503	
6-2	Caspase-12	23.04452315	23.11218987
6-2	Caspase-12	23.10952373	
6-2	Caspase-12	23.18252274	
6-3	Caspase-12	22.83152395	22.87519022
6-3	Caspase-12	22.96352268	
6-3	Caspase-12	22.83052402	
6-4	Caspase-12	22.31852393	22.3125239
6-4	Caspase-12	22.38252474	
6-4	Caspase-12	22.23652302	
6-5	Caspase-12	22.26052415	22.24385768
6-5	Caspase-12	22.10152426	
6-5	Caspase-12	22.36952464	
6-6	Caspase-12	23.61952314	23.55185724
6-6	Caspase-12	23.53552467	
6-6	Caspase-12	23.50052392	

Sample Name	Target Name	CT	Average CT value
1-1	CHOP	31.10052325	30.98519082
1-1	CHOP	30.9965241	
1-1	CHOP	30.85852511	
1-2	CHOP	29.11552354	29.29452376
1-2	CHOP	29.35652347	
1-2	CHOP	29.41152428	
2-1	CHOP	28.62152487	28.51885763
2-1	CHOP	28.39052464	
2-1	CHOP	28.54452337	
2-2	CHOP	29.36052414	29.22219136
2-2	CHOP	29.17352511	
2-2	CHOP	29.13252484	
2-3	CHOP	28.14052541	28.30519134
2-3	CHOP	28.36352484	
2-3	CHOP	28.41152376	
2-4	CHOP	28.21152464	28.13319102
2-4	CHOP	28.09052498	
2-4	CHOP	28.09752344	
2-5	CHOP	29.19052327	29.16152457
2-5	CHOP	29.2785252	
2-5	CHOP	29.01552524	
2-6	CHOP	28.56752345	28.68719031
2-6	CHOP	28.8445233	
2-6	CHOP	28.64952419	
3-1	CHOP	29.94852538	29.96885827
3-1	CHOP	29.98852404	
3-1	CHOP	29.96952539	

3-2	CHOP	27.8845228	27.81352412
3-2	CHOP	27.88452476	
3-2	CHOP	27.67152479	
3-3	CHOP	30.30952465	30.2521902
3-3	CHOP	30.44952341	
3-3	CHOP	29.99752255	
3-4	CHOP	29.49252538	29.50852428
3-4	CHOP	29.32052348	
3-4	CHOP	29.712524	
3-5	CHOP	30.06452253	30.24385627
3-5	CHOP	30.37452326	
3-5	CHOP	30.29252302	
3-6	CHOP	29.87125249	29.74410075
3-6	CHOP	29.53552476	
3-6	CHOP	29.82552501	
4-1	CHOP	30.71352526	30.85385845
4-1	CHOP	30.93052495	
4-1	CHOP	30.91752515	
4-2	CHOP	30.08952461	30.20985754
4-2	CHOP	30.10052327	
4-2	CHOP	30.43952473	
4-3	CHOP	29.48152261	29.3985236
4-3	CHOP	29.58552331	
4-3	CHOP	29.12852487	
4-4	CHOP	30.91152536	31.22419076
4-4	CHOP	31.39152272	
4-4	CHOP	31.36952419	
4-5	CHOP	29.6215226	29.46052429
4-5	CHOP	29.43652504	
4-5	CHOP	29.32352523	
4-6	CHOP	29.39052474	29.38819125
4-6	CHOP	29.22152442	
4-6	CHOP	29.55252459	
5-1	CHOP	29.44852304	29.45385708
5-1	CHOP	29.41952326	
5-1	CHOP	29.49352492	
5-2	CHOP	29.54852426	29.49119088
5-2	CHOP	29.52552418	
5-2	CHOP	29.39952419	
5-3	CHOP	30.22325251	30.27343338
5-3	CHOP	30.26352441	
5-3	CHOP	30.3335232	
5-4	CHOP	29.9845239	29.70052367
5-4	CHOP	29.50652405	
5-4	CHOP	29.61052307	
5-5	CHOP	30.42952327	30.52019012

5-5	CHOP	30.33452438	
5-5	CHOP	30.7965227	
5-6	CHOP	30.45652291	30.54085709
5-6	CHOP	30.53252448	
5-6	CHOP	30.63352389	
6-1	CHOP	29.42452525	29.20719181
6-1	CHOP	29.27152501	
6-1	CHOP	28.92552517	
6-2	CHOP	29.94252428	29.73819043
6-2	CHOP	29.71452418	
6-2	CHOP	29.55752283	
6-3	CHOP	29.53552266	29.49885683
6-3	CHOP	29.47152465	
6-3	CHOP	29.48952317	
6-4	CHOP	28.83752393	28.98485759
6-4	CHOP	29.21552365	
6-4	CHOP	28.9015252	
6-5	CHOP	28.77352322	28.89285704
6-5	CHOP	28.96952491	
6-5	CHOP	28.93552297	
6-6	CHOP	29.95752326	30.21485736
6-6	CHOP	30.28952361	
6-6	CHOP	30.39752521	

Sample Name	Target Name	CT	Average CT value
1-1	PERK	33.66852507	33.39319116
1-1	PERK	33.1545254	
1-1	PERK	33.35652301	
1-2	PERK	32.03952414	31.90052418
1-2	PERK	31.81052399	
1-2	PERK	31.85152441	
2-1	PERK	31.0365237	30.96919071
2-1	PERK	30.86452302	
2-1	PERK	31.0065254	
2-2	PERK	31.54952338	31.67952361
2-2	PERK	31.98552378	
2-2	PERK	31.50352366	
2-3	PERK	30.66752291	30.71019042
2-3	PERK	30.72152523	
2-3	PERK	30.74152312	
2-4	PERK	30.53152512	30.52585761
2-4	PERK	30.69752303	
2-4	PERK	30.34852467	
2-5	PERK	31.92652313	31.59819059
2-5	PERK	31.46452421	
2-5	PERK	31.40352442	

2-6	PERK	31.0685226	31.09652338
2-6	PERK	31.12152428	
2-6	PERK	31.09952328	
3-1	PERK	32.28452312	32.33485698
3-1	PERK	32.36252326	
3-1	PERK	32.35752456	
3-2	PERK	30.28352329	30.27852388
3-2	PERK	30.28352396	
3-2	PERK	30.26852438	
3-3	PERK	32.65752364	32.60452368
3-3	PERK	32.58452411	
3-3	PERK	32.5715233	
3-4	PERK	31.87052429	31.93452428
3-4	PERK	32.06252431	
3-4	PERK	31.87052424	
3-5	PERK	32.91552386	32.70685783
3-5	PERK	32.68052505	
3-5	PERK	32.52452459	
3-6	PERK	32.01452331	32.11219053
3-6	PERK	32.33152433	
3-6	PERK	31.99052396	
4-1	PERK	33.34352358	33.44752341
4-1	PERK	33.32252275	
4-1	PERK	33.67652391	
4-2	PERK	32.65352275	32.75919063
4-2	PERK	32.63352509	
4-2	PERK	32.99052405	
4-3	PERK	31.97552363	31.88352404
4-3	PERK	31.72252494	
4-3	PERK	31.95252357	
4-4	PERK	33.44152498	33.66619127
4-4	PERK	33.67452345	
4-4	PERK	33.88252538	
4-5	PERK	31.84952263	31.81519054
4-5	PERK	31.79752418	
4-5	PERK	31.79852482	
4-6	PERK	31.40852273	31.62585715
4-6	PERK	31.85952521	
4-6	PERK	31.60952349	
5-1	PERK	31.8625246	31.83485793
5-1	PERK	31.92652471	
5-1	PERK	31.71552448	
5-2	PERK	32.30152425	31.89919022
5-2	PERK	32.0855236	
5-2	PERK	31.31052282	
5-3	PERK	32.89752438	32.68519138

5-3	PERK	32.72352479	
5-3	PERK	32.43452496	
5-4	PERK	31.95852482	32.15452398
5-4	PERK	32.22252328	
5-4	PERK	32.28252384	
5-5	PERK	33.00652392	32.95319052
5-5	PERK	32.93152305	
5-5	PERK	32.92152458	
5-6	PERK	33.01152374	32.91219032
5-6	PERK	32.87952321	
5-6	PERK	32.84552402	
6-1	PERK	31.62452395	31.59919076
6-1	PERK	31.57352505	
6-1	PERK	31.59952327	
6-2	PERK	32.37852484	32.16885771
6-2	PERK	32.07252533	
6-2	PERK	32.05552296	
6-3	PERK	31.92552266	31.91319019
6-3	PERK	31.92452422	
6-3	PERK	31.8895237	
6-4	PERK	31.11252384	31.33885778
6-4	PERK	31.34552448	
6-4	PERK	31.55852501	
6-5	PERK	31.01852306	31.2731897
6-5	PERK	31.34652301	
6-5	PERK	31.45452304	
6-6	PERK	32.49952412	32.56919006
6-6	PERK	32.64052252	
6-6	PERK	32.56752353	

Sample Name	Target Name	CT	Average CT value
1-1	eIF2α	33.77952285	33.69385709
1-1	eIF2α	33.602524	
1-1	eIF2α	33.69952442	
1-2	eIF2α	32.41252478	32.2355244
1-2	eIF2α	32.13152441	
1-2	eIF2α	32.16252402	
2-1	eIF2α	31.32752392	31.27919007
2-1	eIF2α	31.41752324	
2-1	eIF2α	31.09252303	
2-2	eIF2α	31.95052489	31.92919052
2-2	eIF2α	31.98652301	
2-2	eIF2α	31.85052367	
2-3	eIF2α	31.35952327	30.96819118
2-3	eIF2α	30.79652504	
2-3	eIF2α	30.74852522	

2-4	eIF2α	30.6665233	30.83252348
2-4	eIF2α	30.94252357	
2-4	eIF2α	30.88852358	
2-5	eIF2α	31.72652506	31.90552473
2-5	eIF2α	31.99952488	
2-5	eIF2α	31.99052425	
2-6	eIF2α	31.19752262	31.3871911
2-6	eIF2α	31.61952541	
2-6	eIF2α	31.34452527	
3-1	eIF2α	32.50152498	32.65019075
3-1	eIF2α	32.68452334	
3-1	eIF2α	32.76452393	
3-2	eIF2α	30.31552332	30.52985721
3-2	eIF2α	30.57352388	
3-2	eIF2α	30.70052443	
3-3	eIF2α	32.91052429	32.89285832
3-3	eIF2α	32.8425254	
3-3	eIF2α	32.92552528	
3-4	eIF2α	32.16752285	32.17185707
3-4	eIF2α	32.14452401	
3-4	eIF2α	32.20352435	
3-5	eIF2α	33.4785233	32.96152414
3-5	eIF2α	32.941525	
3-5	eIF2α	32.46452413	
3-6	eIF2α	32.49152507	32.47352443
3-6	eIF2α	32.54352296	
3-6	eIF2α	32.38552526	
4-1	eIF2α	33.41952526	33.6161914
4-1	eIF2α	33.54252371	
4-1	eIF2α	33.88652522	
4-2	eIF2α	33.1945245	33.20885744
4-2	eIF2α	33.36652276	
4-2	eIF2α	33.06552507	
4-3	eIF2α	32.27852334	32.13252321
4-3	eIF2α	32.0985234	
4-3	eIF2α	32.0205229	
4-4	eIF2α	33.8565524	33.82320056
4-4	eIF2α	33.95652412	
4-4	eIF2α	33.65652516	
4-5	eIF2α	32.49652508	32.35619103
4-5	eIF2α	32.38852393	
4-5	eIF2α	32.18352407	
4-6	eIF2α	32.31452393	32.02952443
4-6	eIF2α	31.97052523	
4-6	eIF2α	31.80352412	
5-1	eIF2α	32.04052275	32.14885626

5-1	eIF2α	32.22252287	
5-1	eIF2α	32.18352315	
5-2	eIF2α	32.16052296	32.19519046
5-2	eIF2α	32.20252301	
5-2	eIF2α	32.22252541	
5-3	eIF2α	32.9775236	32.97919014
5-3	eIF2α	32.98652401	
5-3	eIF2α	32.97352282	
5-4	eIF2α	33.04652416	32.44052418
5-4	eIF2α	32.21552298	
5-4	eIF2α	32.05952539	
5-5	eIF2α	33.20052284	33.24652426
5-5	eIF2α	33.0535247	
5-5	eIF2α	33.48552523	
5-6	eIF2α	33.20452454	33.24819027
5-6	eIF2α	33.24652268	
5-6	eIF2α	33.29352358	
6-1	eIF2α	31.99852515	31.92219119
6-1	eIF2α	31.92752424	
6-1	eIF2α	31.84052419	
6-2	eIF2α	32.57552428	32.44985715
6-2	eIF2α	32.31352438	
6-2	eIF2α	32.46052279	
6-3	eIF2α	32.48552374	32.24385794
6-3	eIF2α	32.34352517	
6-3	eIF2α	31.9025249	
6-4	eIF2α	31.76752466	31.65319087
6-4	eIF2α	31.30452409	
6-4	eIF2α	31.88752386	
6-5	eIF2α	31.63052386	31.60719062
6-5	eIF2α	31.57752451	
6-5	eIF2α	31.6135235	
6-6	eIF2α	32.70952454	32.85385782
6-6	eIF2α	32.85352455	
6-6	eIF2α	32.99852438	

Sample Name	Target Name	CT	Average CT value
1-1	IRE1	23.89152365	23.84085675
1-1	IRE1	23.66152391	
1-1	IRE1	23.96952271	
1-2	IRE1	22.39052349	22.14385661
1-2	IRE1	22.16252291	
1-2	IRE1	21.87852343	
2-1	IRE1	21.43152367	21.39419139
2-1	IRE1	21.35952522	
2-1	IRE1	21.39152529	

2-2	IRE1	22.07352433	22.10619073
2-2	IRE1	22.16952358	
2-2	IRE1	22.07552426	
2-3	IRE1	21.18252302	21.14019019
2-3	IRE1	21.06052272	
2-3	IRE1	21.17752482	
2-4	IRE1	21.03352531	20.99685718
2-4	IRE1	21.03552283	
2-4	IRE1	20.92152339	
2-5	IRE1	21.93452445	22.00685709
2-5	IRE1	22.05652287	
2-5	IRE1	22.02952395	
2-6	IRE1	21.54252295	21.53709588
2-6	IRE1	21.49524122	
2-6	IRE1	21.57352346	
3-1	IRE1	22.72152502	22.80452436
3-1	IRE1	22.70352323	
3-1	IRE1	22.98852483	
3-2	IRE1	20.71052271	20.68385723
3-2	IRE1	20.76552479	
3-2	IRE1	20.5755242	
3-3	IRE1	23.03752509	23.01052352
3-3	IRE1	23.0005228	
3-3	IRE1	22.99352266	
3-4	IRE1	22.24452263	22.35252314
3-4	IRE1	22.4445242	
3-4	IRE1	22.36852257	
3-5	IRE1	22.93452449	23.08019062
3-5	IRE1	22.97452396	
3-5	IRE1	23.3315234	
3-6	IRE1	22.52952331	22.60419018
3-6	IRE1	22.41652364	
3-6	IRE1	22.8665236	
4-1	IRE1	23.5145239	23.8014908
4-1	IRE1	23.96502397	
4-1	IRE1	23.92492451	
4-2	IRE1	23.15952397	23.20519095
4-2	IRE1	23.27652478	
4-2	IRE1	23.1795241	
4-3	IRE1	22.06552509	22.23985758
4-3	IRE1	22.34452354	
4-3	IRE1	22.3095241	
4-4	IRE1	23.99252284	24.07919015
4-4	IRE1	24.3585249	
4-4	IRE1	23.88652272	
4-5	IRE1	22.48352484	22.24819096

4-5	IRE1	22.09252312	
4-5	IRE1	22.16852492	
4-6	IRE1	22.38052527	22.23519087
4-6	IRE1	22.038524	
4-6	IRE1	22.28652334	
5-1	IRE1	22.00552347	22.25952455
5-1	IRE1	22.34252477	
5-1	IRE1	22.4305254	
5-2	IRE1	22.243524	22.278191
5-2	IRE1	22.10252385	
5-2	IRE1	22.48852515	
5-3	IRE1	23.3255253	23.15785753
5-3	IRE1	23.02252346	
5-3	IRE1	23.12552383	
5-4	IRE1	22.53752359	22.5705236
5-4	IRE1	22.5895241	
5-4	IRE1	22.58452313	
5-5	IRE1	23.40952518	23.438191
5-5	IRE1	23.43152273	
5-5	IRE1	23.47352508	
5-6	IRE1	23.39535243	23.37713366
5-6	IRE1	23.49952329	
5-6	IRE1	23.23652526	
6-1	IRE1	22.09452358	22.10052332
6-1	IRE1	22.03752355	
6-1	IRE1	22.16952284	
6-2	IRE1	22.49752255	22.59652386
6-2	IRE1	22.85952505	
6-2	IRE1	22.43252397	
6-3	IRE1	22.19052375	22.32885757
6-3	IRE1	22.25152367	
6-3	IRE1	22.5445253	
6-4	IRE1	21.93452264	21.8501901
6-4	IRE1	21.87352332	
6-4	IRE1	21.74252434	
6-5	IRE1	21.82752316	21.72152349
6-5	IRE1	21.57052264	
6-5	IRE1	21.76652468	
6-6	IRE1	22.93352265	23.06919049
6-6	IRE1	23.28052481	
6-6	IRE1	22.993524	

Sample Name	Target Name	CT	Average CT value
1-1	ATF4	20.25752376	20.47552342
1-1	ATF4	20.17852274	
1-1	ATF4	20.99052376	

1-2	ATF4	18.8765245	18.79419035
1-2	ATF4	18.75052394	
1-2	ATF4	18.75552261	
2-1	ATF4	17.98952419	18.07052443
2-1	ATF4	18.28052535	
2-1	ATF4	17.94152375	
2-2	ATF4	18.87052375	18.77319031
2-2	ATF4	18.75352456	
2-2	ATF4	18.69552262	
2-3	ATF4	17.87652403	17.80152446
2-3	ATF4	17.87352532	
2-3	ATF4	17.65452403	
2-4	ATF4	17.52252284	17.59019092
2-4	ATF4	17.48852462	
2-4	ATF4	17.7595253	
2-5	ATF4	18.61152326	18.64085724
2-5	ATF4	18.7635244	
2-5	ATF4	18.54752405	
2-6	ATF4	18.09952391	18.16385729
2-6	ATF4	18.2915241	
2-6	ATF4	18.10052386	
3-1	ATF4	19.40752401	19.40185669
3-1	ATF4	19.4855232	
3-1	ATF4	19.31252285	
3-2	ATF4	17.30352509	17.325858
3-2	ATF4	17.39552424	
3-2	ATF4	17.27852468	
3-3	ATF4	19.36152542	19.64319069
3-3	ATF4	19.57252268	
3-3	ATF4	19.99552398	
3-4	ATF4	19.18052484	19.00485778
3-4	ATF4	18.87652355	
3-4	ATF4	18.95752494	
3-5	ATF4	19.81452519	19.75785827
3-5	ATF4	19.73952421	
3-5	ATF4	19.71952542	
3-6	ATF4	17.50252353	17.39519117
3-6	ATF4	17.43152512	
3-6	ATF4	17.25152484	
4-1	ATF4	20.43952517	20.36152382
4-1	ATF4	20.27652351	
4-1	ATF4	20.36852277	
4-2	ATF4	19.82752478	19.78767435
4-2	ATF4	19.68252316	
4-2	ATF4	19.8529751	
4-3	ATF4	19.16952467	19.09085713

4-3	ATF4	18.97852406	
4-3	ATF4	19.12452266	
4-4	ATF4	20.85852486	20.71252463
4-4	ATF4	20.71952529	
4-4	ATF4	20.55952373	
4-5	ATF4	19.01152352	19.10052336
4-5	ATF4	19.19452315	
4-5	ATF4	19.09552341	
4-6	ATF4	18.71452523	18.79485753
4-6	ATF4	18.77352427	
4-6	ATF4	18.8965231	
5-1	ATF4	19.02852378	18.93352406
5-1	ATF4	18.9005247	
5-1	ATF4	18.87152369	
5-2	ATF4	18.93852456	18.95285758
5-2	ATF4	18.92252447	
5-2	ATF4	18.99752372	
5-3	ATF4	19.72052269	19.79209987
5-3	ATF4	19.79652447	
5-3	ATF4	19.85925245	
5-4	ATF4	19.35552478	19.2195246
5-4	ATF4	19.09852439	
5-4	ATF4	19.20452464	
5-5	ATF4	20.31252258	20.04485628
5-5	ATF4	19.99852319	
5-5	ATF4	19.82352307	
5-6	ATF4	20.09752334	20.05252386
5-6	ATF4	19.87552541	
5-6	ATF4	20.18452284	
6-1	ATF4	18.86952277	18.67585687
6-1	ATF4	18.47252484	
6-1	ATF4	18.68552301	
6-2	ATF4	19.34352377	19.2618574
6-2	ATF4	19.15852322	
6-2	ATF4	19.2835252	
6-3	ATF4	18.900524	18.95019134
6-3	ATF4	18.62352462	
6-3	ATF4	19.32652539	
6-4	ATF4	18.46352477	18.39819068
6-4	ATF4	18.27352349	
6-4	ATF4	18.45752377	
6-5	ATF4	18.23052343	18.3285244
6-5	ATF4	18.16752438	
6-5	ATF4	18.5875254	
6-6	ATF4	19.85292408	19.63465728
6-6	ATF4	19.42652477	

6-6	ATF4	19.62452299	

Sample Name	Target Name	CT	Average CT value	RQ
1-1	Bcl-2	34.19552481	34.38819086	1
1-1	Bcl-2	34.3805233		
1-1	Bcl-2	34.58852448		
1-2	Bcl-2	33.11852501	32.95152436	0.995389631
1-2	Bcl-2	32.76752348		
1-2	Bcl-2	32.96852459		
2-1	Bcl-2	34.16952533	34.08319176	0.419962427
2-1	Bcl-2	34.25852506		
2-1	Bcl-2	33.8215249		
2-2	Bcl-2	34.50652477	34.64285779	0.458819524
2-2	Bcl-2	34.43052446		
2-2	Bcl-2	34.99152413		
2-3	Bcl-2	33.93152351	33.87819024	0.400349847
2-3	Bcl-2	33.76052353		
2-3	Bcl-2	33.94252369		
2-4	Bcl-2	33.63652407	33.77785734	0.383154791
2-4	Bcl-2	33.75752338		
2-4	Bcl-2	33.93952457		
2-5	Bcl-2	34.61652347	34.64652353	0.436483853
2-5	Bcl-2	34.70952256		
2-5	Bcl-2	34.61352456		
2-6	Bcl-2	34.45152381	34.26885782	0.403786666
2-6	Bcl-2	34.22452497		
2-6	Bcl-2	34.13052467		
3-1	Bcl-2	33.85652428	34.21885719	0.721297745
3-1	Bcl-2	33.96452359		
3-1	Bcl-2	34.8355237		
3-2	Bcl-2	32.19952441	32.17385756	0.69511903
3-2	Bcl-2	32.06252448		
3-2	Bcl-2	32.25952378		
3-3	Bcl-2	34.74752497	34.50385822	0.717972104
3-3	Bcl-2	34.39152469		
3-3	Bcl-2	34.37252499		
3-4	Bcl-2	33.81452266	33.81885648	0.703359485
3-4	Bcl-2	33.76152381		
3-4	Bcl-2	33.88052296		
3-5	Bcl-2	34.3765227	34.46652364	0.765956027
3-5	Bcl-2	34.37852528		
3-5	Bcl-2	34.64452293		
3-6	Bcl-2	34.29152342	33.98685737	0.74103288
3-6	Bcl-2	33.95052337		
3-6	Bcl-2	33.71852533		

4-1	Bcl-2	34.37352273	34.42652312	1.034309196
4-1	Bcl-2	34.43052381		
4-1	Bcl-2	34.47552283		
4-2	Bcl-2	32.96252402	33.60919059	1.190581715
4-2	Bcl-2	33.90752332		
4-2	Bcl-2	33.95752445		
4-3	Bcl-2	32.62252317	32.73818983	1.132099393
4-3	Bcl-2	32.96852319		
4-3	Bcl-2	32.62352314		
4-4	Bcl-2	34.92752353	34.75219039	1.006955563
4-4	Bcl-2	34.51852479		
4-4	Bcl-2	34.81052286		
4-5	Bcl-2	33.13752391	33.00352364	1.010217833
4-5	Bcl-2	32.79952395		
4-5	Bcl-2	33.07352307		
4-6	Bcl-2	32.85052303	32.80485738	0.991029891
4-6	Bcl-2	32.60252412		
4-6	Bcl-2	32.961525		
5-1	Bcl-2	34.03952509	34.14052404	0.622868932
5-1	Bcl-2	34.23352404		
5-1	Bcl-2	34.14852299		
5-2	Bcl-2	34.19252434	34.22285751	0.606957
5-2	Bcl-2	34.2325228		
5-2	Bcl-2	34.24352539		
5-3	Bcl-2	34.94352519	34.90652377	0.654289125
5-3	Bcl-2	34.8675233		
5-3	Bcl-2	34.90852281		
5-4	Bcl-2	34.33152358	34.42552365	0.627926111
5-4	Bcl-2	34.61952442		
5-4	Bcl-2	34.32552297		
5-5	Bcl-2	35.3105247	35.32652378	0.599154565
5-5	Bcl-2	35.43752311		
5-5	Bcl-2	35.23152353		
5-6	Bcl-2	35.27952416	35.25752421	0.615998685
5-6	Bcl-2	35.32452499		
5-6	Bcl-2	35.1685235		
6-1	Bcl-2	33.66152518	33.65152435	0.697694085
6-1	Bcl-2	33.56252468		
6-1	Bcl-2	33.73052319		
6-2	Bcl-2	34.21152447	34.04652402	0.768614872
6-2	Bcl-2	33.98252354		
6-2	Bcl-2	33.94552403		
6-3	Bcl-2	33.9275235	33.93152401	0.705311699
6-3	Bcl-2	33.96052511		
6-3	Bcl-2	33.90652342		
6-4	Bcl-2	33.27352445	33.3931907	0.701087136

6-4	Bcl-2	33.6165246		
6-4	Bcl-2	33.28952303		
6-5	Bcl-2	33.26852411	33.28952408	0.711038698
6-5	Bcl-2	33.16552416		
6-5	Bcl-2	33.43452398		
6-6	Bcl-2	34.49315251	34.57973298	0.721693329
6-6	Bcl-2	34.72252264		
6-6	Bcl-2	34.52352379		

Sample Name	Target Name	CT	Average CT value	RQ
1-1	Bax	32.74952292	32.85019028	1
1-1	Bax	32.83252344		
1-1	Bax	32.96852447		
1-2	Bax	31.26335243	31.34546714	1.043470562
1-2	Bax	31.49252527		
1-2	Bax	31.28052371		
2-1	Bax	30.22452338	30.40085679	1.856604543
2-1	Bax	30.48252445		
2-1	Bax	30.49552254		
2-2	Bax	31.12852453	31.11019104	1.828505352
2-2	Bax	31.07552523		
2-2	Bax	31.12652336		
2-3	Bax	30.16952385	30.14452367	1.834007004
2-3	Bax	30.01052359		
2-3	Bax	30.25352358		
2-4	Bax	30.09152353	29.97985694	1.835278583
2-4	Bax	29.84552455		
2-4	Bax	30.00252273		
2-5	Bax	31.04452276	31.04185638	1.828507769
2-5	Bax	31.1845234		
2-5	Bax	30.89652298		
2-6	Bax	30.55052383	30.54719046	1.834430116
2-6	Bax	30.57052389		
2-6	Bax	30.52052367		
3-1	Bax	31.74852409	31.75819029	1.367303139
3-1	Bax	31.563523		
3-1	Bax	31.96252378		
3-2	Bax	29.95752329	29.69519013	1.334222054
3-2	Bax	29.69552255		
3-2	Bax	29.43252454		
3-3	Bax	32.00852328	32.05318968	1.35159943
3-3	Bax	32.07652281		
3-3	Bax	32.07452296		
3-4	Bax	31.04752359	31.31252392	1.376177069
3-4	Bax	31.38552426		

3-4	Bax	31.50452391		
3-5	Bax	31.97952276	32.07519047	1.383829367
3-5	Bax	32.19052349		
3-5	Bax	32.05552515		
3-6	Bax	31.3285242	31.5821909	1.351231954
3-6	Bax	31.68352428		
3-6	Bax	31.73452422		
4-1	Bax	32.56825245	32.82010009	1.084545051
4-1	Bax	32.92752357		
4-1	Bax	32.96452424		
4-2	Bax	32.00352306	32.28985767	1.023136751
4-2	Bax	32.49552524		
4-2	Bax	32.37052471		
4-3	Bax	31.2585232	31.32219078	1.04029961
4-3	Bax	31.44652378		
4-3	Bax	31.26152537		
4-4	Bax	33.41152496	33.25819159	0.976707336
4-4	Bax	33.16652493		
4-4	Bax	33.19652489		
4-5	Bax	31.67052537	31.46252436	1.012319794
4-5	Bax	31.40652505		
4-5	Bax	31.31052267		
4-6	Bax	31.40552486	31.15652367	1.069794437
4-6	Bax	31.04552288		
4-6	Bax	31.01852326		
5-1	Bax	31.14952514	31.28319146	1.554372753
5-1	Bax	31.36652462		
5-1	Bax	31.33352462		
5-2	Bax	31.41352468	31.33849986	1.543305077
5-2	Bax	31.43745234		
5-2	Bax	31.16452257		
5-3	Bax	32.14752534	32.09719061	1.579352023
5-3	Bax	32.1555239		
5-3	Bax	31.98852259		
5-4	Bax	31.5595254	31.61052391	1.521680915
5-4	Bax	31.68652254		
5-4	Bax	31.5855238		
5-5	Bax	32.3045245	32.39485751	1.574251256
5-5	Bax	32.3765251		
5-5	Bax	32.50352293		
5-6	Bax	32.46552323	32.37085675	1.568805025
5-6	Bax	32.14552331		
5-6	Bax	32.50152371		
6-1	Bax	31.03852458	31.08119113	1.427013663
6-1	Bax	31.27852516		
6-1	Bax	30.92652364		

6-2	Bax	31.63852276	31.60252284	1.440264103
6-2	Bax	31.37452269		
6-2	Bax	31.79452308		
6-3	Bax	31.30852347	31.37119082	1.432629432
6-3	Bax	31.54952542		
6-3	Bax	31.25552357		
6-4	Bax	30.84352484	30.84185794	1.415192039
6-4	Bax	30.80452466		
6-4	Bax	30.87752433		
6-5	Bax	30.64352376	30.71185708	1.461719419
6-5	Bax	30.82052365		
6-5	Bax	30.67152382		
6-6	Bax	31.7515234	32.04252439	1.442594502
6-6	Bax	32.12352464		
6-6	Bax	32.25252513		

Sample Name	Target Name	CT	Average CT value	RQ
1-1	Caspase-3	26.91952286	27.02219014	1
1-1	Caspase-3	27.26952427		
1-1	Caspase-3	26.87752329		
1-2	Caspase-3	25.36523899	25.44842937	1.094618034
1-2	Caspase-3	25.45652494		
1-2	Caspase-3	25.52352419		
2-1	Caspase-3	24.41652424	24.61218982	1.806670475
2-1	Caspase-3	24.71552257		
2-1	Caspase-3	24.70452264		
2-2	Caspase-3	25.26552469	25.28085737	1.830196283
2-2	Caspase-3	25.16952468		
2-2	Caspase-3	25.40752276		
2-3	Caspase-3	24.31252275	24.31785735	1.832312192
2-3	Caspase-3	24.3495242		
2-3	Caspase-3	24.29152511		
2-4	Caspase-3	24.12352454	24.1525241	1.834429902
2-4	Caspase-3	24.08252458		
2-4	Caspase-3	24.25152317		
2-5	Caspase-3	25.16552363	25.23985653	1.795849532
2-5	Caspase-3	25.44952328		
2-5	Caspase-3	25.10452267		
2-6	Caspase-3	24.86552304	24.70418956	1.853603553
2-6	Caspase-3	24.7545226		
2-6	Caspase-3	24.49252303		
3-1	Caspase-3	25.82752385	25.94519058	1.353160255
3-1	Caspase-3	26.01452517		
3-1	Caspase-3	25.99352273		
3-2	Caspase-3	23.86552433	23.82885674	1.370148294

3-2	Caspase-3	23.9335226		
3-2	Caspase-3	23.68752329		
3-3	Caspase-3	26.37552436	26.20852334	1.367303822
3-3	Caspase-3	26.059523		
3-3	Caspase-3	26.19052266		
3-4	Caspase-3	25.452523	25.5251904	1.337927085
3-4	Caspase-3	25.56552393		
3-4	Caspase-3	25.55752429		
3-5	Caspase-3	26.29052413	26.30452414	1.329913357
3-5	Caspase-3	26.29852401		
3-5	Caspase-3	26.32452427		
3-6	Caspase-3	25.77652476	25.76380006	1.342261765
3-6	Caspase-3	25.9453523		
3-6	Caspase-3	25.56952314		
4-1	Caspase-3	27.28352308	27.13176018	0.984477238
4-1	Caspase-3	27.06652384		
4-1	Caspase-3	27.04523362		
4-2	Caspase-3	26.64052372	26.46585734	1.020304077
4-2	Caspase-3	26.1855236		
4-2	Caspase-3	26.57152471		
4-3	Caspase-3	25.38552378	25.33585688	1.160971746
4-3	Caspase-3	25.45052366		
4-3	Caspase-3	25.17152319		
4-4	Caspase-3	27.16252465	27.21052395	1.137341689
4-4	Caspase-3	27.25952354		
4-4	Caspase-3	27.20952366		
4-5	Caspase-3	25.42152391	25.55452395	1.07004049
4-5	Caspase-3	25.34352517		
4-5	Caspase-3	25.89852277		
4-6	Caspase-3	25.4885241	25.36552424	1.04270634
4-6	Caspase-3	25.24352486		
4-6	Caspase-3	25.36452375		
5-1	Caspase-3	25.54352314	25.44719014	1.563017256
5-1	Caspase-3	25.21952437		
5-1	Caspase-3	25.57852291		
5-2	Caspase-3	25.40952297	25.53919066	1.512916487
5-2	Caspase-3	25.55852492		
5-2	Caspase-3	25.64952408		
5-3	Caspase-3	26.21152431	26.34119129	1.502465596
5-3	Caspase-3	26.58952483		
5-3	Caspase-3	26.22252473		
5-4	Caspase-3	25.63152475	25.79819104	1.505245294
5-4	Caspase-3	25.88352422		
5-4	Caspase-3	25.87952415		
5-5	Caspase-3	26.62052428	26.61843197	1.518967736
5-5	Caspase-3	26.45524655		

5-5	Caspase-3	26.77952507		
5-6	Caspase-3	26.69852478	26.54352424	1.568079211
5-6	Caspase-3	26.50752273		
5-6	Caspase-3	26.4245252		
6-1	Caspase-3	25.34652337	25.23119056	1.448941732
6-1	Caspase-3	25.41052326		
6-1	Caspase-3	24.93652505		
6-2	Caspase-3	25.78452402	25.80252394	1.412579584
6-2	Caspase-3	25.95552494		
6-2	Caspase-3	25.66752287		
6-3	Caspase-3	25.79752529	25.54185764	1.433953781
6-3	Caspase-3	25.36852398		
6-3	Caspase-3	25.45952366		
6-4	Caspase-3	24.99652441	24.96152408	1.467470569
6-4	Caspase-3	24.9535247		
6-4	Caspase-3	24.93452312		
6-5	Caspase-3	24.79952519	24.88419096	1.461381036
6-5	Caspase-3	24.87752481		
6-5	Caspase-3	24.97552288		
6-6	Caspase-3	26.42052312	26.23385658	1.423392466
6-6	Caspase-3	26.28452399		
6-6	Caspase-3	25.99652262		

Sample Name	Target Name	CT	Average CT value	RQ
1-1	Caspase-12	24.54052503	24.37152412	1
1-1	Caspase-12	24.24852403		
1-1	Caspase-12	24.32552329		
1-2	Caspase-12	22.05752423	22.68219141	1.185914066
1-2	Caspase-12	22.03752521		
1-2	Caspase-12	23.95152478		
2-1	Caspase-12	21.42352407	21.92752405	1.84975364
2-1	Caspase-12	21.36052508		
2-1	Caspase-12	22.99852298		
2-2	Caspase-12	22.44252302	22.64990023	1.805363697
2-2	Caspase-12	22.87865238		
2-2	Caspase-12	22.62852528		
2-3	Caspase-12	21.74552314	21.67652422	1.820497101
2-3	Caspase-12	21.40852439		
2-3	Caspase-12	21.87552511		
2-4	Caspase-12	21.50752374	21.51219036	1.821339019
2-4	Caspase-12	21.40652371		
2-4	Caspase-12	21.62252363		
2-5	Caspase-12	22.34652338	22.53519003	1.864342529
2-5	Caspase-12	22.75652298		
2-5	Caspase-12	22.50252374		

2-6	Caspase-12	22.02552371	22.09085706	1.806252007
2-6	Caspase-12	22.13152404		
2-6	Caspase-12	22.11552342		
3-1	Caspase-12	23.38452293	23.30418986	1.344125088
3-1	Caspase-12	23.3955237		
3-1	Caspase-12	23.13252295		
3-2	Caspase-12	21.07952466	21.19052487	1.358484295
3-2	Caspase-12	21.30452527		
3-2	Caspase-12	21.18752468		
3-3	Caspase-12	23.40952295	23.59352329	1.333916058
3-3	Caspase-12	23.6675238		
3-3	Caspase-12	23.70352311		
3-4	Caspase-12	22.94852436	22.8925245	1.32133783
3-4	Caspase-12	22.95552505		
3-4	Caspase-12	22.77352409		
3-5	Caspase-12	23.46552257	23.63218964	1.350038663
3-5	Caspase-12	23.72852301		
3-5	Caspase-12	23.70252335		
3-6	Caspase-12	23.09652301	23.10252305	1.352170465
3-6	Caspase-12	23.03652332		
3-6	Caspase-12	23.17452281		
4-1	Caspase-12	24.67952325	24.5361906	0.947588968
4-1	Caspase-12	24.46452475		
4-1	Caspase-12	24.4645238		
4-2	Caspase-12	23.57352319	23.6931901	1.110339137
4-2	Caspase-12	23.90352445		
4-2	Caspase-12	23.60252266		
4-3	Caspase-12	22.85052319	22.95052429	0.965935945
4-3	Caspase-12	22.94652482		
4-3	Caspase-12	23.05452485		
4-4	Caspase-12	24.25052417	24.26485738	1.395387957
4-4	Caspase-12	24.29552283		
4-4	Caspase-12	24.24852514		
4-5	Caspase-12	23.07852285	23.03652366	0.976031526
4-5	Caspase-12	23.01852378		
4-5	Caspase-12	23.01252436		
4-6	Caspase-12	22.95052382	22.75300015	1.015500512
4-6	Caspase-12	22.74695244		
4-6	Caspase-12	22.56152419		
5-1	Caspase-12	23.00252277	22.84852331	1.507684447
5-1	Caspase-12	22.83652268		
5-1	Caspase-12	22.70652448		
5-2	Caspase-12	22.84252389	22.89585738	1.505246337
5-2	Caspase-12	22.7595248		
5-2	Caspase-12	23.08552344		
5-3	Caspase-12	23.53652542	23.62985803	1.566993495

5-3	Caspase-12	23.53252329		
5-3	Caspase-12	23.82052538		
5-4	Caspase-12	23.16752325	23.13285668	1.520627659
5-4	Caspase-12	23.28552268		
5-4	Caspase-12	22.94552412		
5-5	Caspase-12	24.02052434	23.9408577	1.54756442
5-5	Caspase-12	23.83052445		
5-5	Caspase-12	23.97152432		
5-6	Caspase-12	23.79052442	23.92119061	1.537584859
5-6	Caspase-12	23.93952443		
5-6	Caspase-12	24.03352299		
6-1	Caspase-12	22.48152342	22.61752379	1.412254723
6-1	Caspase-12	22.58852291		
6-1	Caspase-12	22.78252503		
6-2	Caspase-12	23.04452315	23.11218987	1.451958477
6-2	Caspase-12	23.10952373		
6-2	Caspase-12	23.18252274		
6-3	Caspase-12	22.83152395	22.87519022	1.449946758
6-3	Caspase-12	22.96352268		
6-3	Caspase-12	22.83052402		
6-4	Caspase-12	22.31852393	22.3125239	1.465777092
6-4	Caspase-12	22.38252474		
6-4	Caspase-12	22.23652302		
6-5	Caspase-12	22.26052415	22.24385768	1.450951846
6-5	Caspase-12	22.10152426		
6-5	Caspase-12	22.36952464		
6-6	Caspase-12	23.61952314	23.55185724	1.454644703
6-6	Caspase-12	23.53552467		
6-6	Caspase-12	23.50052392		

Sample Name	Target Name	CT	Average CT value	RQ
1-1	CHOP	31.10052325	30.98519082	1
1-1	CHOP	30.9965241		
1-1	CHOP	30.85852511		
1-2	CHOP	29.11552354	29.29452376	1.187011427
1-2	CHOP	29.35652347		
1-2	CHOP	29.41152428		
2-1	CHOP	28.62152487	28.51885763	1.878610876
2-1	CHOP	28.39052464		
2-1	CHOP	28.54452337		
2-2	CHOP	29.36052414	29.22219136	1.857889983
2-2	CHOP	29.17352511		
2-2	CHOP	29.13252484		
2-3	CHOP	28.14052541	28.30519134	1.801666559

2-3	CHOP	28.36352484		
2-3	CHOP	28.41152376		
2-4	CHOP	28.21152464	28.13319102	1.812103721
2-4	CHOP	28.09052498		
2-4	CHOP	28.09752344		
2-5	CHOP	29.19052327	29.16152457	1.848044008
2-5	CHOP	29.2785252		
2-5	CHOP	29.01552524		
2-6	CHOP	28.56752345	28.68719031	1.828084353
2-6	CHOP	28.8445233		
2-6	CHOP	28.64952419		
3-1	CHOP	29.94852538	29.96885827	1.297438094
3-1	CHOP	29.98852404		
3-1	CHOP	29.96952539		
3-2	CHOP	27.8845228	27.81352412	1.349724865
3-2	CHOP	27.88452476		
3-2	CHOP	27.67152479		
3-3	CHOP	30.30952465	30.2521902	1.292951064
3-3	CHOP	30.44952341		
3-3	CHOP	29.99752255		
3-4	CHOP	29.49252538	29.50852428	1.319202736
3-4	CHOP	29.32052348		
3-4	CHOP	29.712524		
3-5	CHOP	30.06452253	30.24385627	1.351911591
3-5	CHOP	30.37452326		
3-5	CHOP	30.29252302		
3-6	CHOP	29.87125249	29.74410075	1.326262222
3-6	CHOP	29.53552476		
3-6	CHOP	29.82552501		
4-1	CHOP	30.71352526	30.85385845	1.163387868
4-1	CHOP	30.93052495		
4-1	CHOP	30.91752515		
4-2	CHOP	30.08952461	30.20985754	1.18755939
4-2	CHOP	30.10052327		
4-2	CHOP	30.43952473		
4-3	CHOP	29.48152261	29.3985236	1.083475719
4-3	CHOP	29.58552331		
4-3	CHOP	29.12852487		
4-4	CHOP	30.91152536	31.22419076	1.098092524
4-4	CHOP	31.39152272		
4-4	CHOP	31.36952419		
4-5	CHOP	29.6215226	29.46052429	1.113163671
4-5	CHOP	29.43652504		
4-5	CHOP	29.32352523		
4-6	CHOP	29.39052474	29.38819125	1.00046214
4-6	CHOP	29.22152442		

4-6	CHOP	29.55252459		
5-1	CHOP	29.44852304	29.45385708	1.516417962
5-1	CHOP	29.41952326		
5-1	CHOP	29.49352492		
5-2	CHOP	29.54852426	29.49119088	1.524496475
5-2	CHOP	29.52552418		
5-2	CHOP	29.39952419		
5-3	CHOP	30.22325251	30.27343338	1.534842427
5-3	CHOP	30.26352441		
5-3	CHOP	30.3335232		
5-4	CHOP	29.9845239	29.70052367	1.56989346
5-4	CHOP	29.50652405		
5-4	CHOP	29.61052307		
5-5	CHOP	30.42952327	30.52019012	1.58483622
5-5	CHOP	30.33452438		
5-5	CHOP	30.7965227		
5-6	CHOP	30.45652291	30.54085709	1.531203744
5-6	CHOP	30.53252448		
5-6	CHOP	30.63352389		
6-1	CHOP	29.42452525	29.20719181	1.435943526
6-1	CHOP	29.27152501		
6-1	CHOP	28.92552517		
6-2	CHOP	29.94252428	29.73819043	1.439598343
6-2	CHOP	29.71452418		
6-2	CHOP	29.55752283		
6-3	CHOP	29.53552266	29.49885683	1.439931342
6-3	CHOP	29.47152465		
6-3	CHOP	29.48952317		
6-4	CHOP	28.83752393	28.98485759	1.407367141
6-4	CHOP	29.21552365		
6-4	CHOP	28.9015252		
6-5	CHOP	28.77352322	28.89285704	1.415848592
6-5	CHOP	28.96952491		
6-5	CHOP	28.93552297		
6-6	CHOP	29.95752326	30.21485736	1.405743529
6-6	CHOP	30.28952361		
6-6	CHOP	30.39752521		

Sample Name	Target Name	CT	Average CT value	RQ
1-1	PERK	33.66852507	33.39319116	1
1-1	PERK	33.1545254		
1-1	PERK	33.35652301		
1-2	PERK	32.03952414	31.90052418	1.034786935
1-2	PERK	31.81052399		
1-2	PERK	31.85152441		

2-1	PERK	31.0365237	30.96919071	1.824288024
2-1	PERK	30.86452302		
2-1	PERK	31.0065254		
2-2	PERK	31.54952338	31.67952361	1.795434682
2-2	PERK	31.98552378		
2-2	PERK	31.50352366		
2-3	PERK	30.66752291	30.71019042	1.805418488
2-3	PERK	30.72152523		
2-3	PERK	30.74152312		
2-4	PERK	30.53152512	30.52585761	1.831466469
2-4	PERK	30.69752303		
2-4	PERK	30.34852467		
2-5	PERK	31.92652313	31.59819059	1.811686637
2-5	PERK	31.46452421		
2-5	PERK	31.40352442		
2-6	PERK	31.0685226	31.09652338	1.826396383
2-6	PERK	31.12152428		
2-6	PERK	31.09952328		
3-1	PERK	32.28452312	32.33485698	1.335766029
3-1	PERK	32.36252326		
3-1	PERK	32.35752456		
3-2	PERK	30.28352329	30.27852388	1.297438295
3-2	PERK	30.28352396		
3-2	PERK	30.26852438		
3-3	PERK	32.65752364	32.60452368	1.343814992
3-3	PERK	32.58452411		
3-3	PERK	32.5715233		
3-4	PERK	31.87052429	31.93452428	1.302846069
3-4	PERK	32.06252431		
3-4	PERK	31.87052424		
3-5	PERK	32.91552386	32.70685783	1.301341477
3-5	PERK	32.68052505		
3-5	PERK	32.52452459		
3-6	PERK	32.01452331	32.11219053	1.363463993
3-6	PERK	32.33152433		
3-6	PERK	31.99052396		
4-1	PERK	33.34352358	33.44752341	1.022901722
4-1	PERK	33.32252275		
4-1	PERK	33.67652391		
4-2	PERK	32.65352275	32.75919063	1.076737748
4-2	PERK	32.63352509		
4-2	PERK	32.99052405		
4-3	PERK	31.97552363	31.88352404	1.027164122
4-3	PERK	31.72252494		
4-3	PERK	31.95252357		
4-4	PERK	33.44152498	33.66619127	1.072516204

4-4	PERK	33.67452345		
4-4	PERK	33.88252538		
4-5	PERK	31.84952263	31.81519054	1.155085641
4-5	PERK	31.79752418		
4-5	PERK	31.79852482		
4-6	PERK	31.40852273	31.62585715	1.125839379
4-6	PERK	31.85952521		
4-6	PERK	31.60952349		
5-1	PERK	31.8625246	31.83485793	1.545064355
5-1	PERK	31.92652471		
5-1	PERK	31.71552448		
5-2	PERK	32.30152425	31.89919022	1.524497524
5-2	PERK	32.0855236		
5-2	PERK	31.31052282		
5-3	PERK	32.89752438	32.68519138	1.53084996
5-3	PERK	32.72352479		
5-3	PERK	32.43452496		
5-4	PERK	31.95852482	32.15452398	1.520627393
5-4	PERK	32.22252328		
5-4	PERK	32.28252384		
5-5	PERK	33.00652392	32.95319052	1.557609617
5-5	PERK	32.93152305		
5-5	PERK	32.92152458		
5-6	PERK	33.01152374	32.91219032	1.570619125
5-6	PERK	32.87952321		
5-6	PERK	32.84552402		
6-1	PERK	31.62452395	31.59919076	1.451958687
6-1	PERK	31.57352505		
6-1	PERK	31.59952327		
6-2	PERK	32.37852484	32.16885771	1.417156811
6-2	PERK	32.07252533		
6-2	PERK	32.05552296		
6-3	PERK	31.92552266	31.91319019	1.433624299
6-3	PERK	31.92452422		
6-3	PERK	31.8895237		
6-4	PERK	31.11252384	31.33885778	1.461043254
6-4	PERK	31.34552448		
6-4	PERK	31.55852501		
6-5	PERK	31.01852306	31.2731897	1.443263448
6-5	PERK	31.34652301		
6-5	PERK	31.45452304		
6-6	PERK	32.49952412	32.56919006	1.459021403
6-6	PERK	32.64052252		
6-6	PERK	32.56752353		

Sample Name	Target Name	CT	Average CT value	RQ
1-1	eIF2α	33.77952285	33.69385709	1
1-1	eIF2α	33.602524		
1-1	eIF2α	33.69952442		
1-2	eIF2α	32.41252478	32.2355244	1.010451061
1-2	eIF2α	32.13152441		
1-2	eIF2α	32.16252402		
2-1	eIF2α	31.32752392	31.27919007	1.812523995
2-1	eIF2α	31.41752324		
2-1	eIF2α	31.09252303		
2-2	eIF2α	31.95052489	31.92919052	1.860038113
2-2	eIF2α	31.98652301		
2-2	eIF2α	31.85052367		
2-3	eIF2α	31.35952327	30.96819118	1.859607898
2-3	eIF2α	30.79652504		
2-3	eIF2α	30.74852522		
2-4	eIF2α	30.6665233	30.83252348	1.823865499
2-4	eIF2α	30.94252357		
2-4	eIF2α	30.88852358		
2-5	eIF2α	31.72652506	31.90552473	1.803332249
2-5	eIF2α	31.99952488		
2-5	eIF2α	31.99052425		
2-6	eIF2α	31.19752262	31.3871911	1.8390977
2-6	eIF2α	31.61952541		
2-6	eIF2α	31.34452527		
3-1	eIF2α	32.50152498	32.65019075	1.322254139
3-1	eIF2α	32.68452334		
3-1	eIF2α	32.76452393		
3-2	eIF2α	30.31552332	30.52985721	1.342571128
3-2	eIF2α	30.57352388		
3-2	eIF2α	30.70052443		
3-3	eIF2α	32.91052429	32.89285832	1.355350344
3-3	eIF2α	32.8425254		
3-3	eIF2α	32.92552528		
3-4	eIF2α	32.16752285	32.17185707	1.361313906
3-4	eIF2α	32.14452401		
3-4	eIF2α	32.20352435		
3-5	eIF2α	33.4785233	32.96152414	1.343502685
3-5	eIF2α	32.941525		
3-5	eIF2α	32.46452413		
3-6	eIF2α	32.49152507	32.47352443	1.307316658
3-6	eIF2α	32.54352296		
3-6	eIF2α	32.38552526		
4-1	eIF2α	33.41952526	33.6161914	1.120906148
4-1	eIF2α	33.54252371		

4-1	eIF2α	33.88652522		
4-2	eIF2α	33.1945245	33.20885744	0.97108267
4-2	eIF2α	33.36652276		
4-2	eIF2α	33.06552507		
4-3	eIF2α	32.27852334	32.13252321	1.064616237
4-3	eIF2α	32.0985234		
4-3	eIF2α	32.0205229		
4-4	eIF2α	33.8565524	33.82320056	1.184810296
4-4	eIF2α	33.95652412		
4-4	eIF2α	33.65652516		
4-5	eIF2α	32.49652508	32.35619103	0.977836568
4-5	eIF2α	32.38852393		
4-5	eIF2α	32.18352407		
4-6	eIF2α	32.31452393	32.02952443	1.04826223
4-6	eIF2α	31.97052523		
4-6	eIF2α	31.80352412		
5-1	eIF2α	32.04052275	32.14885626	1.530851708
5-1	eIF2α	32.22252287		
5-1	eIF2α	32.18352315		
5-2	eIF2α	32.16052296	32.19519046	1.529435749
5-2	eIF2α	32.20252301		
5-2	eIF2α	32.22252541		
5-3	eIF2α	32.9775236	32.97919014	1.537940888
5-3	eIF2α	32.98652401		
5-3	eIF2α	32.97352282		
5-4	eIF2α	33.04652416	32.44052418	1.536164182
5-4	eIF2α	32.21552298		
5-4	eIF2α	32.05952539		
5-5	eIF2α	33.20052284	33.24652426	1.565545984
5-5	eIF2α	33.0535247		
5-5	eIF2α	33.48552523		
5-6	eIF2α	33.20452454	33.24819027	1.532619278
5-6	eIF2α	33.24652268		
5-6	eIF2α	33.29352358		
6-1	eIF2α	31.99852515	31.92219119	1.429653864
6-1	eIF2α	31.92752424		
6-1	eIF2α	31.84052419		
6-2	eIF2α	32.57552428	32.44985715	1.436607443
6-2	eIF2α	32.31352438		
6-2	eIF2α	32.46052279		
6-3	eIF2α	32.48552374	32.24385794	1.404118973
6-3	eIF2α	32.34352517		
6-3	eIF2α	31.9025249		
6-4	eIF2α	31.76752466	31.65319087	1.447267626
6-4	eIF2α	31.30452409		
6-4	eIF2α	31.88752386		

6-5	eIF2α	31.63052386	31.60719062	1.410297644
6-5	eIF2α	31.57752451		
6-5	eIF2α	31.6135235		
6-6	eIF2α	32.70952454	32.85385782	1.475290651
6-6	eIF2α	32.85352455		
6-6	eIF2α	32.99852438		

Sample Name	Target Name	CT	Average CT value	RQ
1-1	IRE1	23.89152365	23.84085675	1
1-1	IRE1	23.66152391		
1-1	IRE1	23.96952271		
1-2	IRE1	22.39052349	22.14385661	1.192233574
1-2	IRE1	22.16252291		
1-2	IRE1	21.87852343		
2-1	IRE1	21.43152367	21.39419139	1.853174104
2-1	IRE1	21.35952522		
2-1	IRE1	21.39152529		
2-2	IRE1	22.07352433	22.10619073	1.821758397
2-2	IRE1	22.16952358		
2-2	IRE1	22.07552426		
2-3	IRE1	21.18252302	21.14019019	1.827661772
2-3	IRE1	21.06052272		
2-3	IRE1	21.17752482		
2-4	IRE1	21.03352531	20.99685718	1.802082809
2-4	IRE1	21.03552283		
2-4	IRE1	20.92152339		
2-5	IRE1	21.93452445	22.00685709	1.86132828
2-5	IRE1	22.05652287		
2-5	IRE1	22.02952395		
2-6	IRE1	21.54252295	21.53709588	1.835398084
2-6	IRE1	21.49524122		
2-6	IRE1	21.57352346		
3-1	IRE1	22.72152502	22.80452436	1.315549506
3-1	IRE1	22.70352323		
3-1	IRE1	22.98852483		
3-2	IRE1	20.71052271	20.68385723	1.336072376
3-2	IRE1	20.76552479		
3-2	IRE1	20.5755242		
3-3	IRE1	23.03752509	23.01052352	1.383190906
3-3	IRE1	23.0005228		
3-3	IRE1	22.99352266		
3-4	IRE1	22.24452263	22.35252314	1.329914385
3-4	IRE1	22.4445242		
3-4	IRE1	22.36852257		
3-5	IRE1	22.93452449	23.08019062	1.370148618

3-5	IRE1	22.97452396		
3-5	IRE1	23.3315234		
3-6	IRE1	22.52952331	22.60419018	1.322201948
3-6	IRE1	22.41652364		
3-6	IRE1	22.8665236		
4-1	IRE1	23.5145239	23.8014908	1.091540568
4-1	IRE1	23.96502397		
4-1	IRE1	23.92492451		
4-2	IRE1	23.15952397	23.20519095	1.077981322
4-2	IRE1	23.27652478		
4-2	IRE1	23.1795241		
4-3	IRE1	22.06552509	22.23985758	1.094292771
4-3	IRE1	22.34452354		
4-3	IRE1	22.3095241		
4-4	IRE1	23.99252284	24.07919015	1.098599965
4-4	IRE1	24.3585249		
4-4	IRE1	23.88652272		
4-5	IRE1	22.48352484	22.24819096	1.166887106
4-5	IRE1	22.09252312		
4-5	IRE1	22.16852492		
4-6	IRE1	22.38052527	22.23519087	1.006490029
4-6	IRE1	22.038524		
4-6	IRE1	22.28652334		
5-1	IRE1	22.00552347	22.25952455	1.569892649
5-1	IRE1	22.34252477		
5-1	IRE1	22.4305254		
5-2	IRE1	22.243524	22.278191	1.598810133
5-2	IRE1	22.10252385		
5-2	IRE1	22.48852515		
5-3	IRE1	23.3255253	23.15785753	1.504550285
5-3	IRE1	23.02252346		
5-3	IRE1	23.12552383		
5-4	IRE1	22.53752359	22.5705236	1.5543729
5-4	IRE1	22.5895241		
5-4	IRE1	22.58452313		
5-5	IRE1	23.40952518	23.438191	1.517817996
5-5	IRE1	23.43152273		
5-5	IRE1	23.47352508		
5-6	IRE1	23.39535243	23.37713366	1.551921543
5-6	IRE1	23.49952329		
5-6	IRE1	23.23652526		
6-1	IRE1	22.09452358	22.10052332	1.39893936
6-1	IRE1	22.03752355		
6-1	IRE1	22.16952284		
6-2	IRE1	22.49752255	22.59652386	1.43693903
6-2	IRE1	22.85952505		

6-2	IRE1	22.43252397		
6-3	IRE1	22.19052375	22.32885757	1.465776567
6-3	IRE1	22.25152367		
6-3	IRE1	22.5445253		
6-4	IRE1	21.93452264	21.8501901	1.397968795
6-4	IRE1	21.87352332		
6-4	IRE1	21.74252434		
6-5	IRE1	21.82752316	21.72152349	1.44259514
6-5	IRE1	21.57052264		
6-5	IRE1	21.76652468		
6-6	IRE1	22.93352265	23.06919049	1.407042796
6-6	IRE1	23.28052481		
6-6	IRE1	22.993524		

Sample Name	Target Name	CT	Average CT value	RQ
1-1	ATF4	20.25752376	20.47552342	1
1-1	ATF4	20.17852274		
1-1	ATF4	20.99052376		
1-2	ATF4	18.8765245	18.79419035	1.179356455
1-2	ATF4	18.75052394		
1-2	ATF4	18.75552261		
2-1	ATF4	17.98952419	18.07052443	1.800418203
2-1	ATF4	18.28052535		
2-1	ATF4	17.94152375		
2-2	ATF4	18.87052375	18.77319031	1.781384223
2-2	ATF4	18.75352456		
2-2	ATF4	18.69552262		
2-3	ATF4	17.87652403	17.80152446	1.79418854
2-3	ATF4	17.87352532		
2-3	ATF4	17.65452403		
2-4	ATF4	17.52252284	17.59019092	1.854458822
2-4	ATF4	17.48852462		
2-4	ATF4	17.7595253		
2-5	ATF4	18.61152326	18.64085724	1.862188405
2-5	ATF4	18.7635244		
2-5	ATF4	18.54752405		
2-6	ATF4	18.09952391	18.16385729	1.845482758
2-6	ATF4	18.2915241		
2-6	ATF4	18.10052386		
3-1	ATF4	19.40752401	19.40185669	1.350037874
3-1	ATF4	19.4855232		
3-1	ATF4	19.31252285		
3-2	ATF4	17.30352509	17.325858	1.329297536
3-2	ATF4	17.39552424		
3-2	ATF4	17.27852468		

3-3	ATF4	19.36152542	19.64319069	1.385109256
3-3	ATF4	19.57252268		
3-3	ATF4	19.99552398		
3-4	ATF4	19.18052484	19.00485778	1.3137269
3-4	ATF4	18.87652355		
3-4	ATF4	18.95752494		
3-5	ATF4	19.81452519	19.75785827	1.329912566
3-5	ATF4	19.73952421		
3-5	ATF4	19.71952542		
3-6	ATF4	17.50252353	17.39519117	4.745658296
3-6	ATF4	17.43152512		
3-6	ATF4	17.25152484		
4-1	ATF4	20.43952517	20.36152382	1.149494365
4-1	ATF4	20.27652351		
4-1	ATF4	20.36852277		
4-2	ATF4	19.82752478	19.78767435	1.117686397
4-2	ATF4	19.68252316		
4-2	ATF4	19.8529751		
4-3	ATF4	19.16952467	19.09085713	0.941913111
4-3	ATF4	18.97852406		
4-3	ATF4	19.12452266		
4-4	ATF4	20.85852486	20.71252463	1.099614886
4-4	ATF4	20.71952529		
4-4	ATF4	20.55952373		
4-5	ATF4	19.01152352	19.10052336	1.003471234
4-5	ATF4	19.19452315		
4-5	ATF4	19.09552341		
4-6	ATF4	18.71452523	18.79485753	1.060197383
4-6	ATF4	18.77352427		
4-6	ATF4	18.8965231		
5-1	ATF4	19.02852378	18.93352406	1.527670138
5-1	ATF4	18.9005247		
5-1	ATF4	18.87152369		
5-2	ATF4	18.93852456	18.95285758	1.555090675
5-2	ATF4	18.92252447		
5-2	ATF4	18.99752372		
5-3	ATF4	19.72052269	19.79209987	1.504992872
5-3	ATF4	19.79652447		
5-3	ATF4	19.85925245		
5-4	ATF4	19.35552478	19.2195246	1.539005428
5-4	ATF4	19.09852439		
5-4	ATF4	19.20452464		
5-5	ATF4	20.31252258	20.04485628	1.547565197
5-5	ATF4	19.99852319		
5-5	ATF4	19.82352307		
5-6	ATF4	20.09752334	20.05252386	1.508727324

5-6	ATF4	19.87552541		
5-6	ATF4	20.18452284		
6-1	ATF4	18.86952277	18.67585687	1.45767242
6-1	ATF4	18.47252484		
6-1	ATF4	18.68552301		
6-2	ATF4	19.34352377	19.2618574	1.406716856
6-2	ATF4	19.15852322		
6-2	ATF4	19.2835252		
6-3	ATF4	18.900524	18.95019134	1.47938557
6-3	ATF4	18.62352462		
6-3	ATF4	19.32652539		
6-4	ATF4	18.46352477	18.39819068	1.484521767
6-4	ATF4	18.27352349		
6-4	ATF4	18.45752377		
6-5	ATF4	18.23052343	18.3285244	1.470525929
6-5	ATF4	18.16752438		
6-5	ATF4	18.5875254		
6-6	ATF4	19.85292408	19.63465728	1.476177371
6-6	ATF4	19.42652477		
6-6	ATF4	19.62452299		
