# Supplementary material for: Sodium formononetin-3'-sulphonate alleviates cerebral ischemia–reperfusion injury in rats via suppressing endoplasmic reticulum stress-mediated apoptosis
Source: BMC Neurosci. 2022 Dec 9;23:74. doi: 10.1186/s12868-022-00762-4 (PMC9733209; doi:10.1186/s12868-022-00762-4)

βactin-1 Gel electrophoresis map

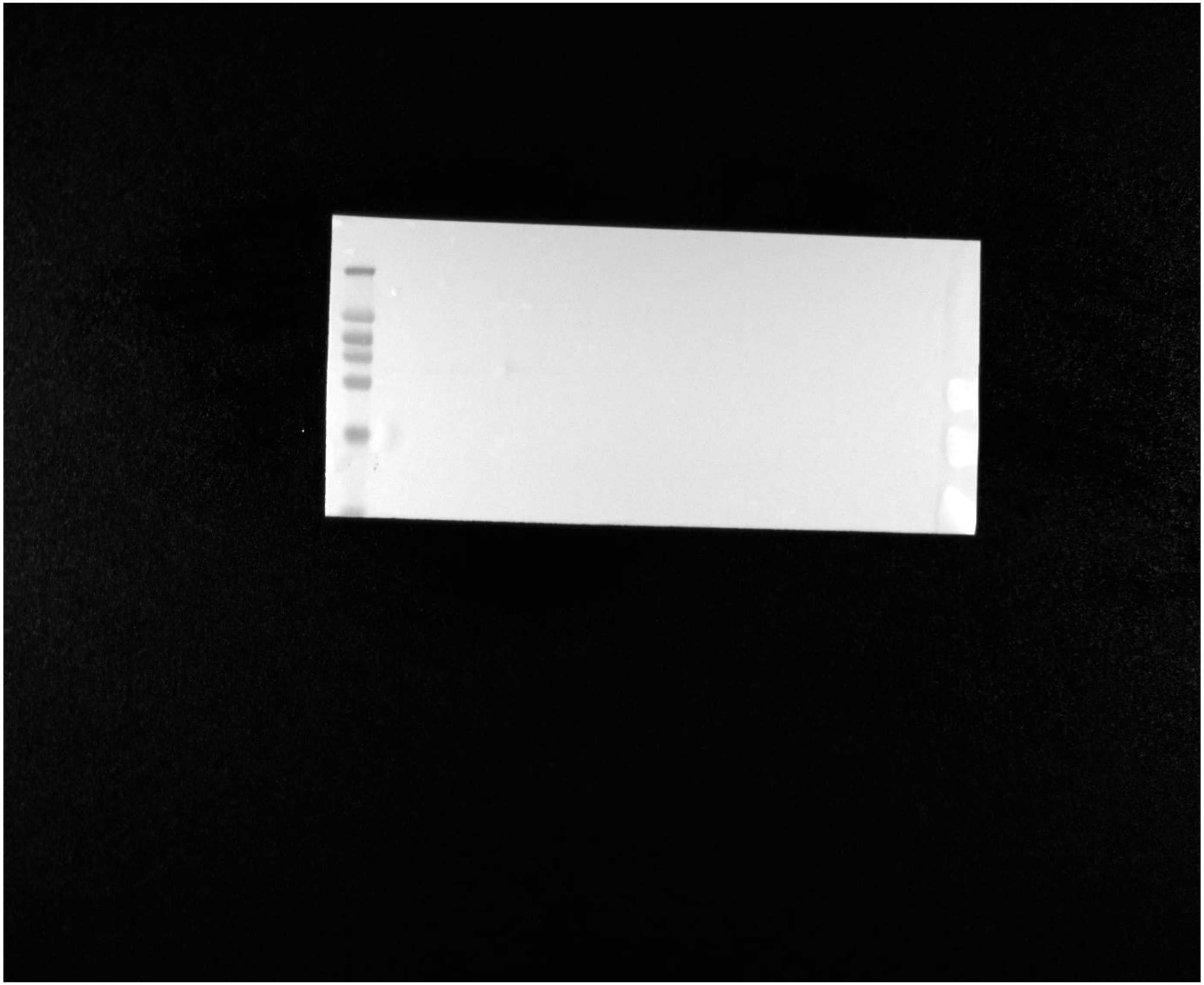

βactin-1 PVDF image

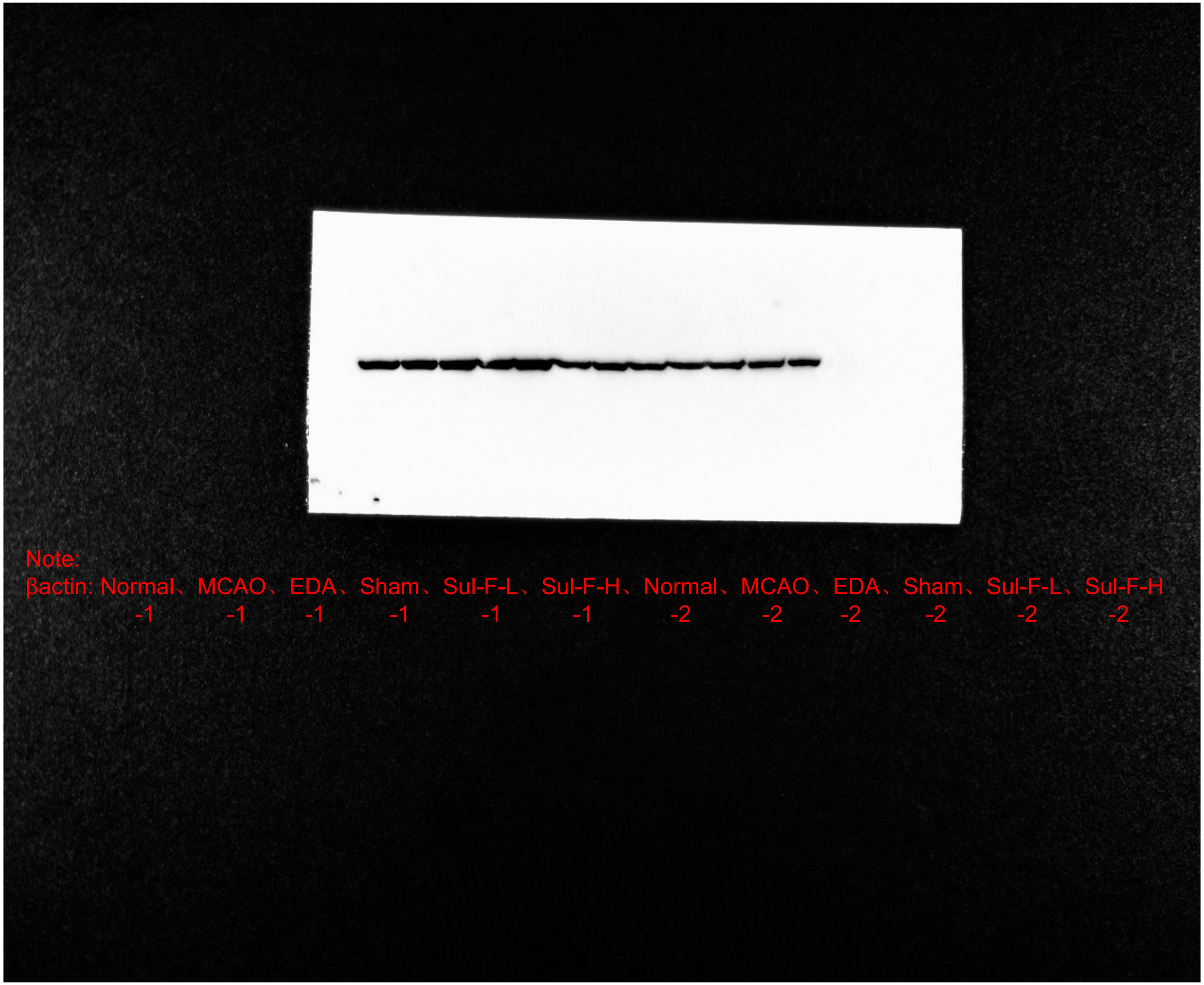

βactin-2 Gel electrophoresis map

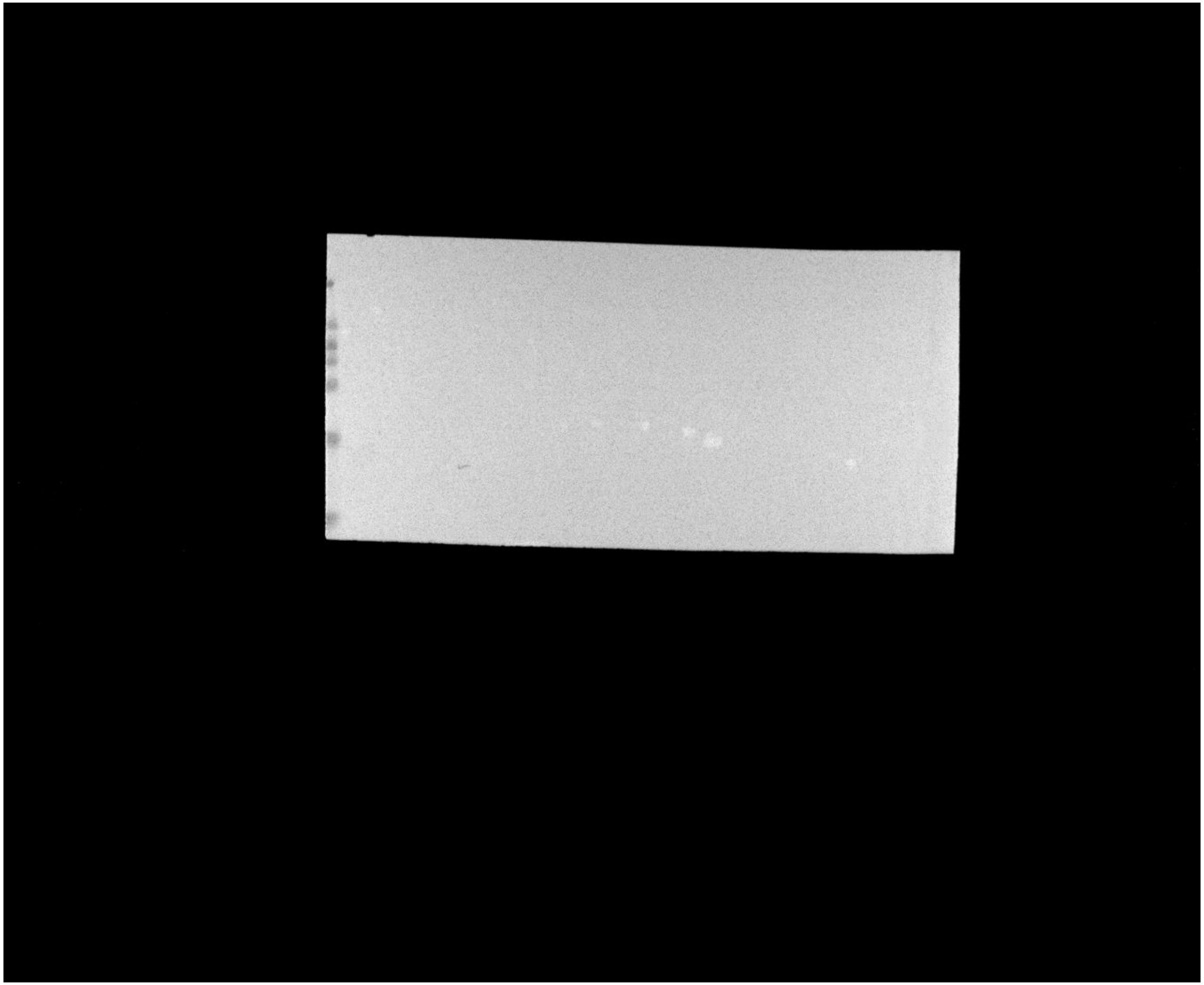

βactin-2 PVDF image

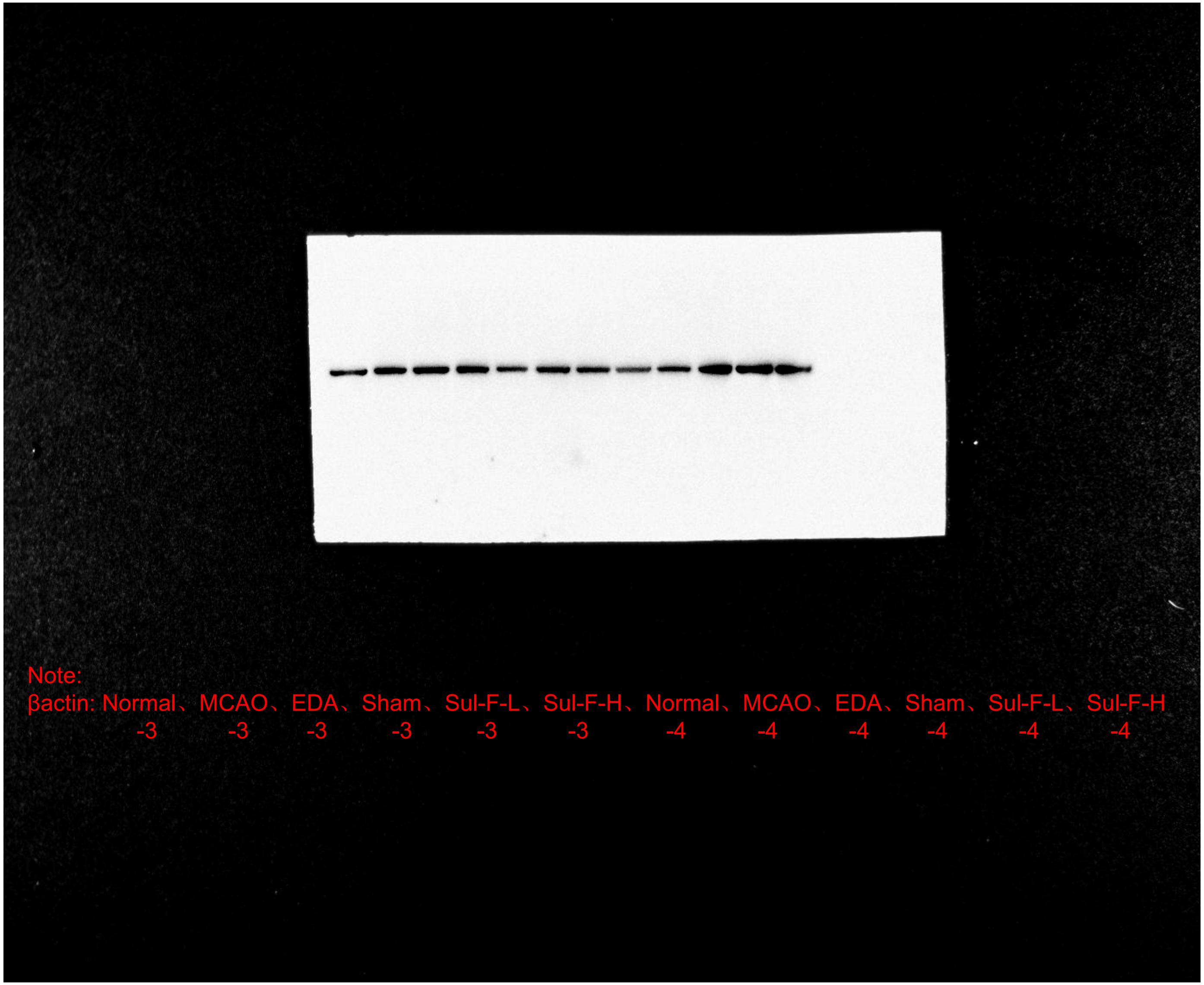

βactin-3 Gel electrophoresis map

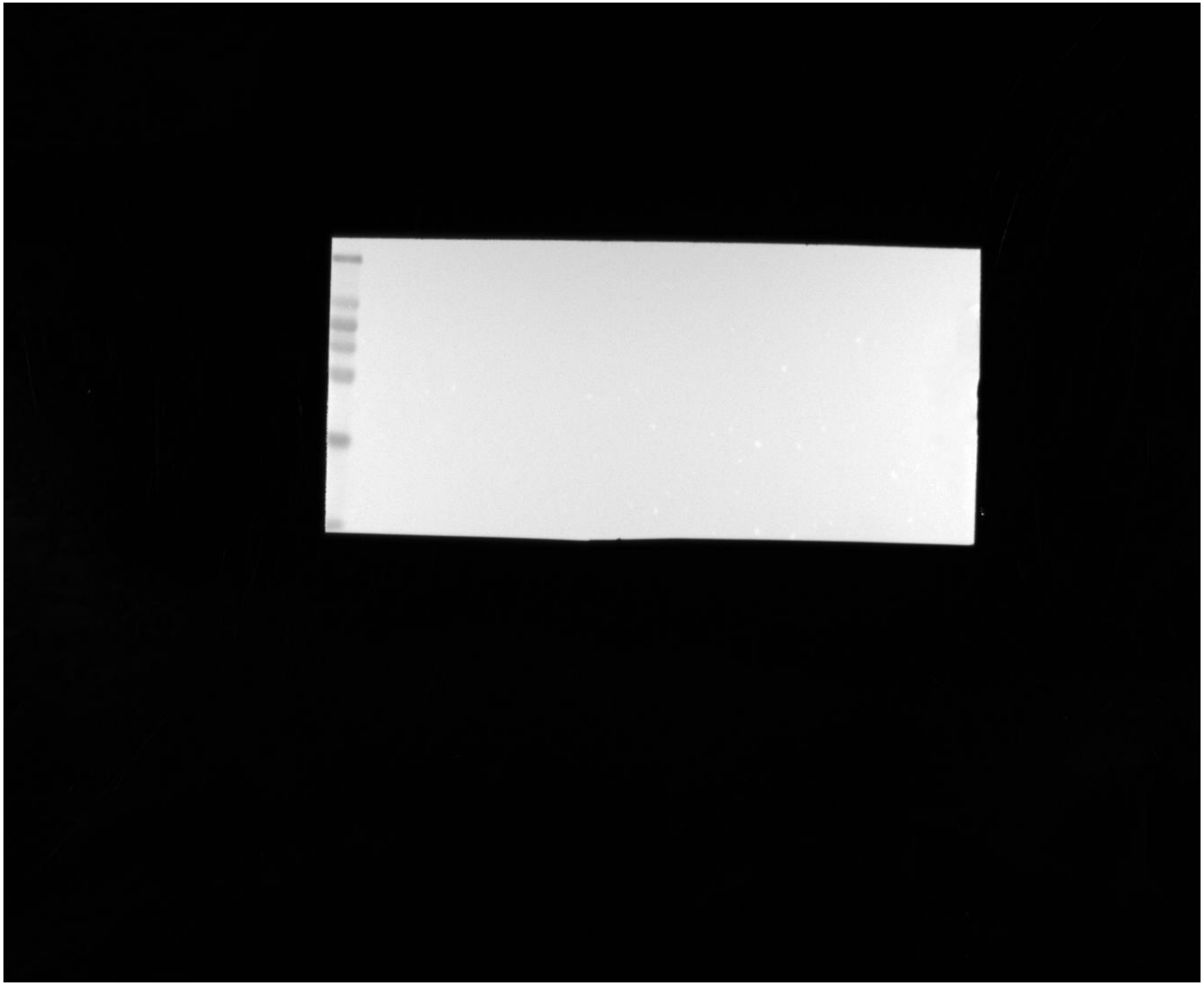

βactin-3 PVDF image

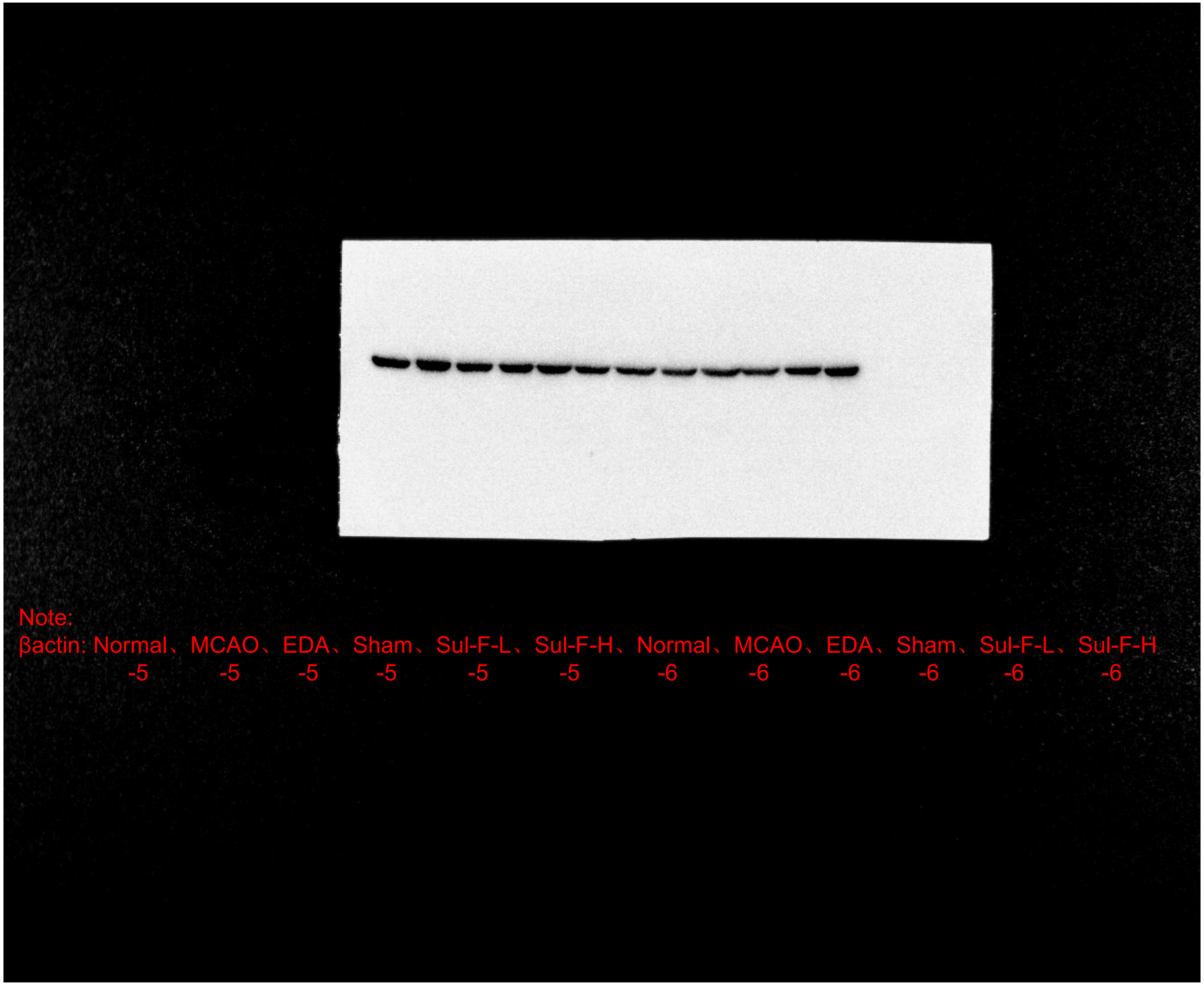

p-PERK Gel electrophoresis map-1

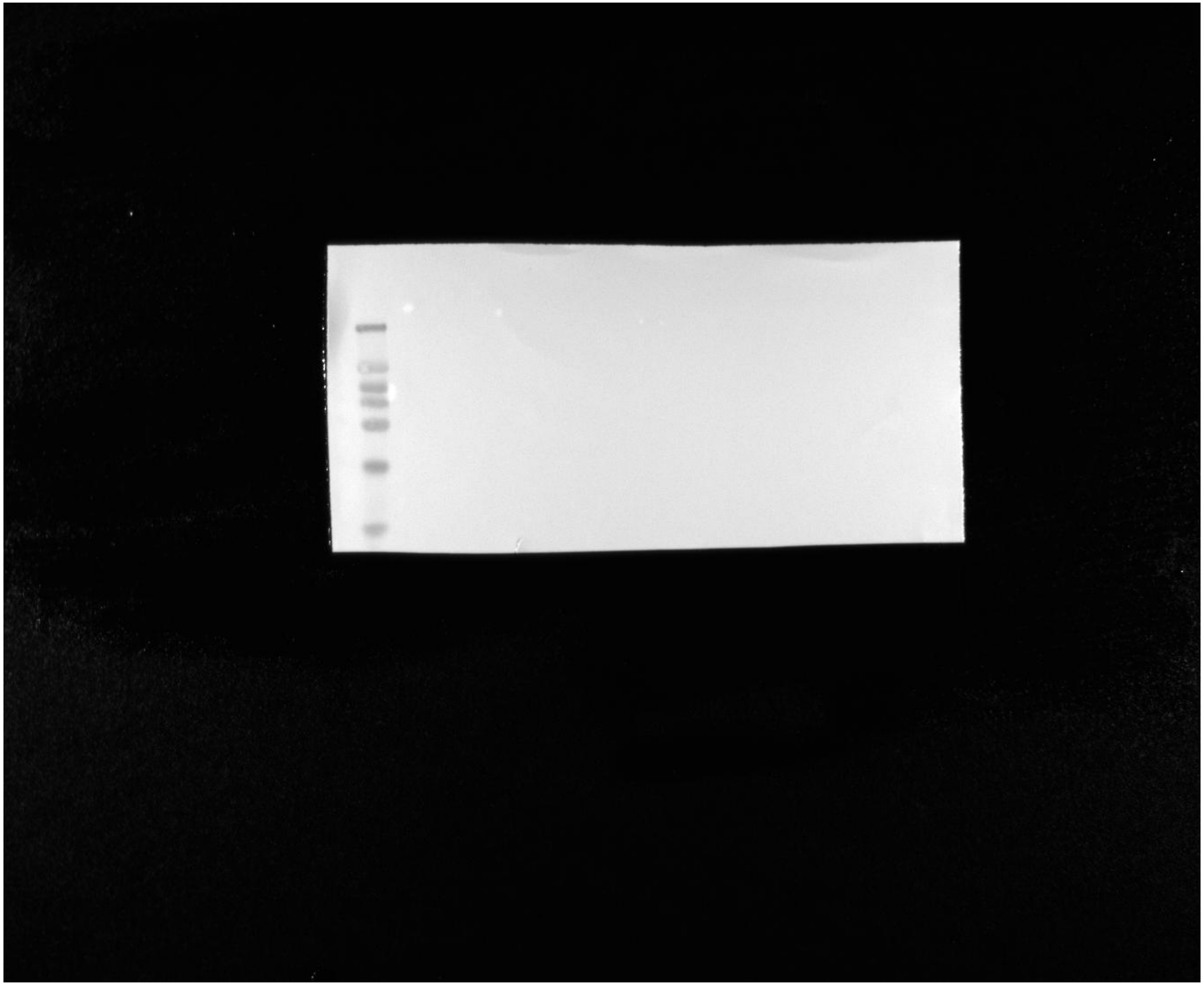

p-PERK PVDF image-1

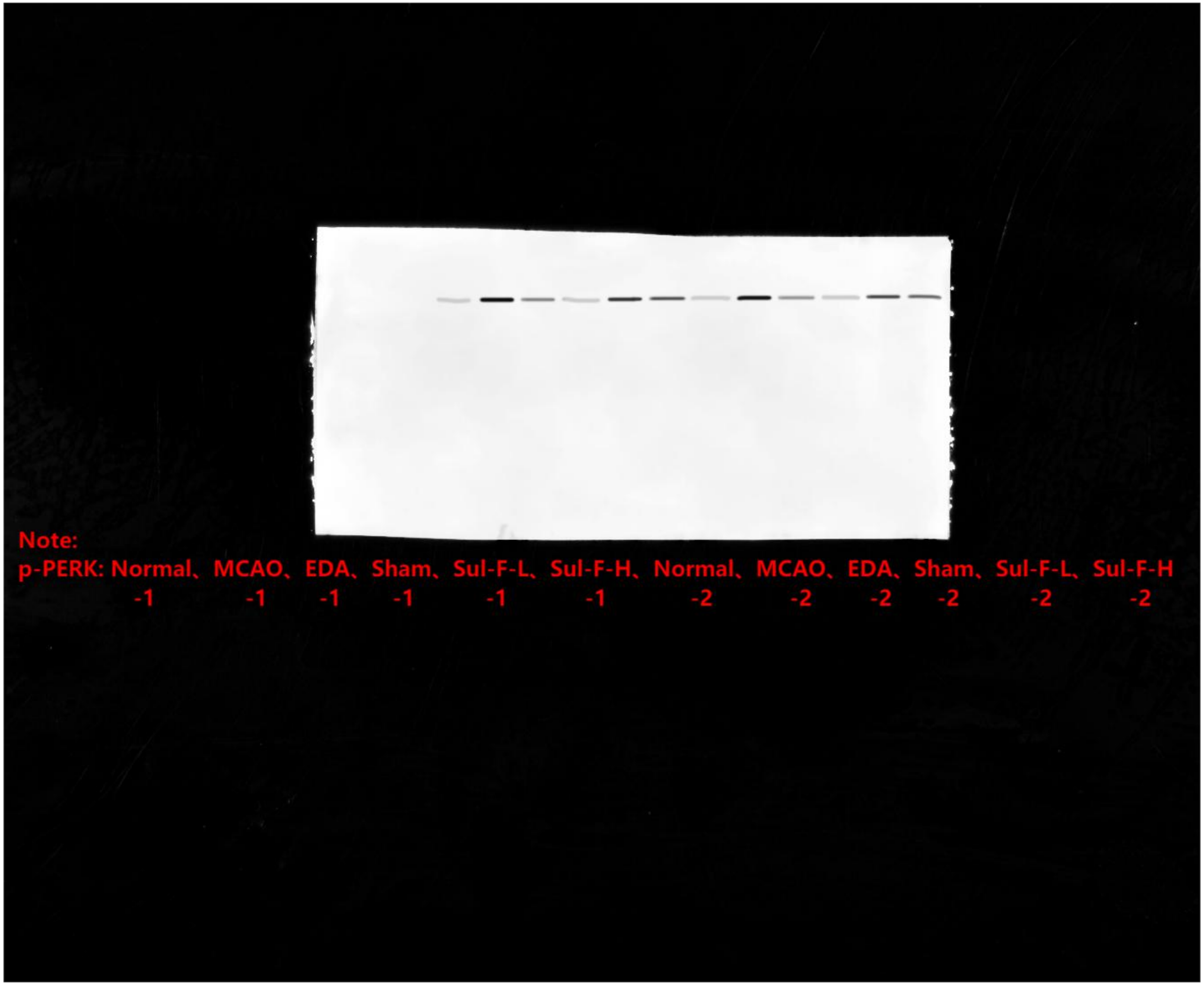

p-PERK Gel electrophoresis map-2

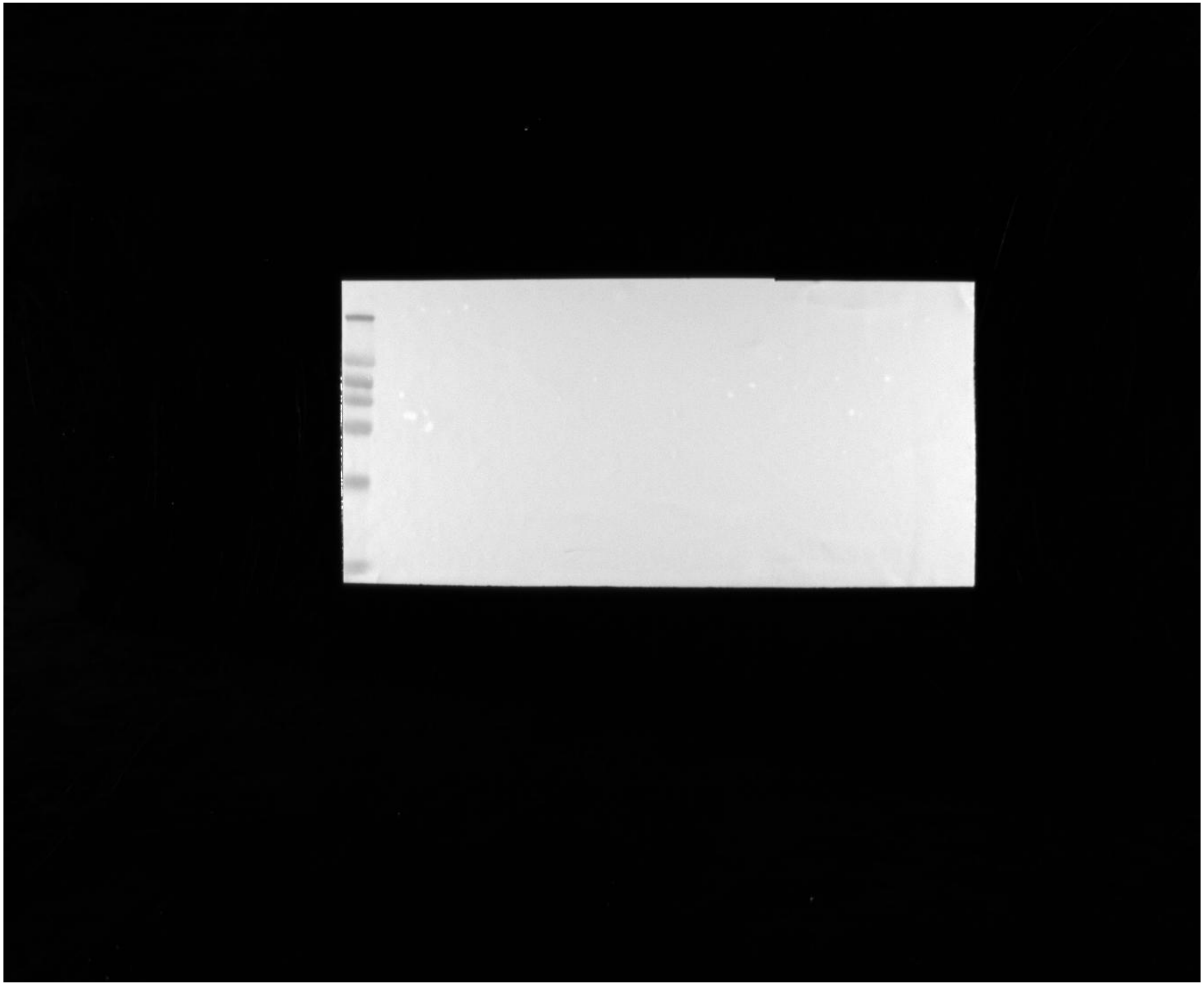

p-PERK PVDF image-2

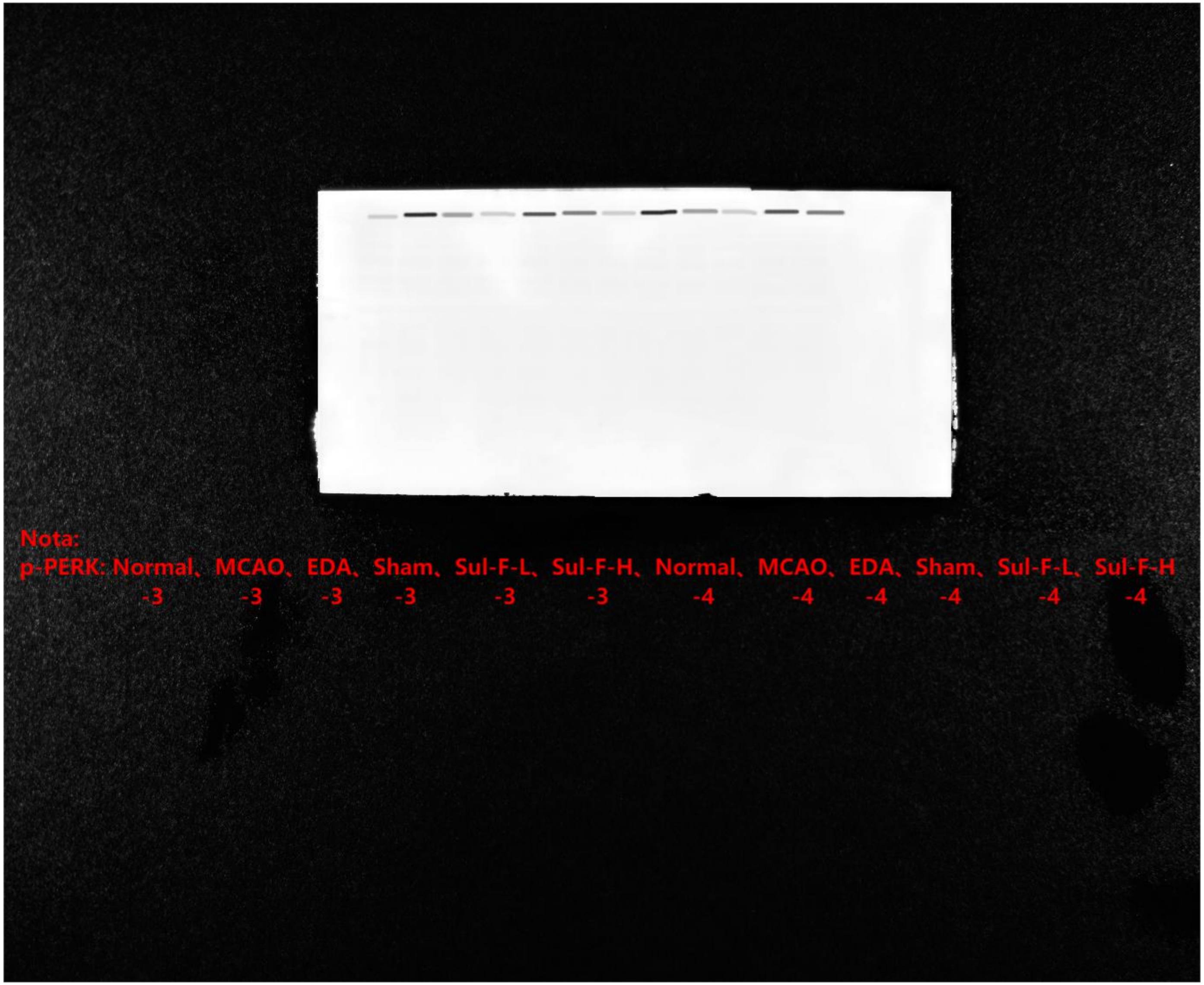

p-PERK Gel electrophoresis map-3

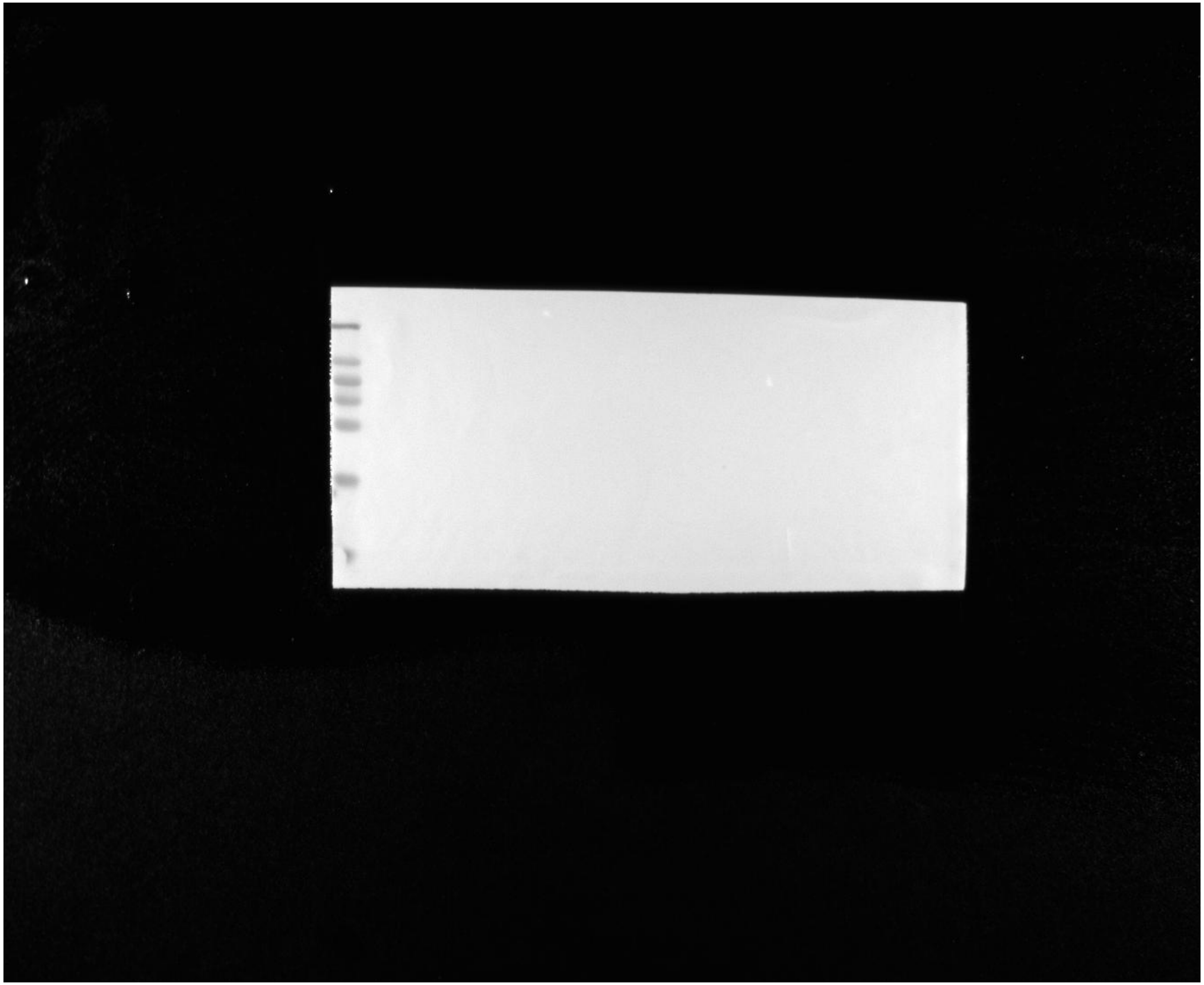

p-PERK PVDF image-3

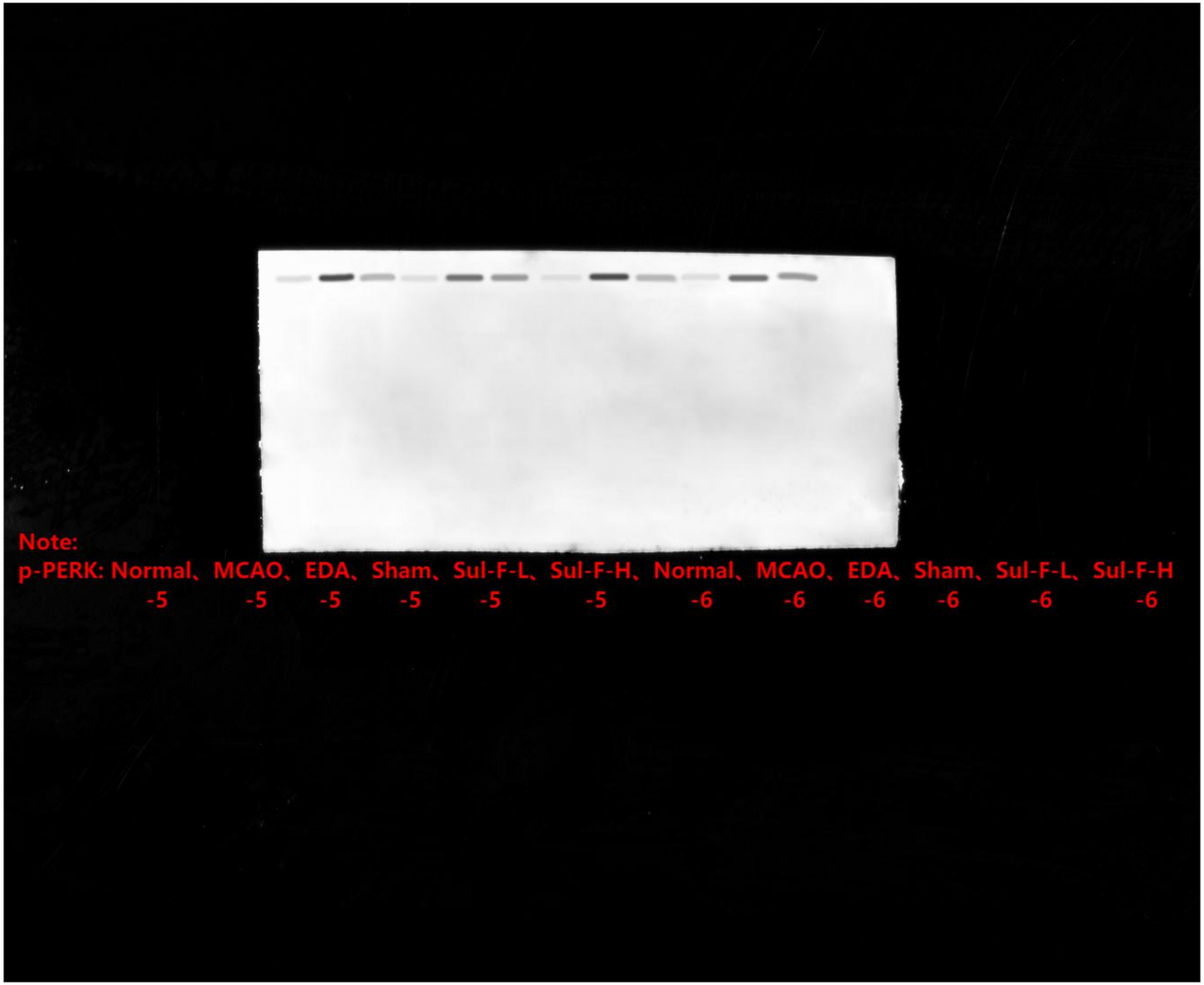

p-eIF2α Gel electrophoresis map-1

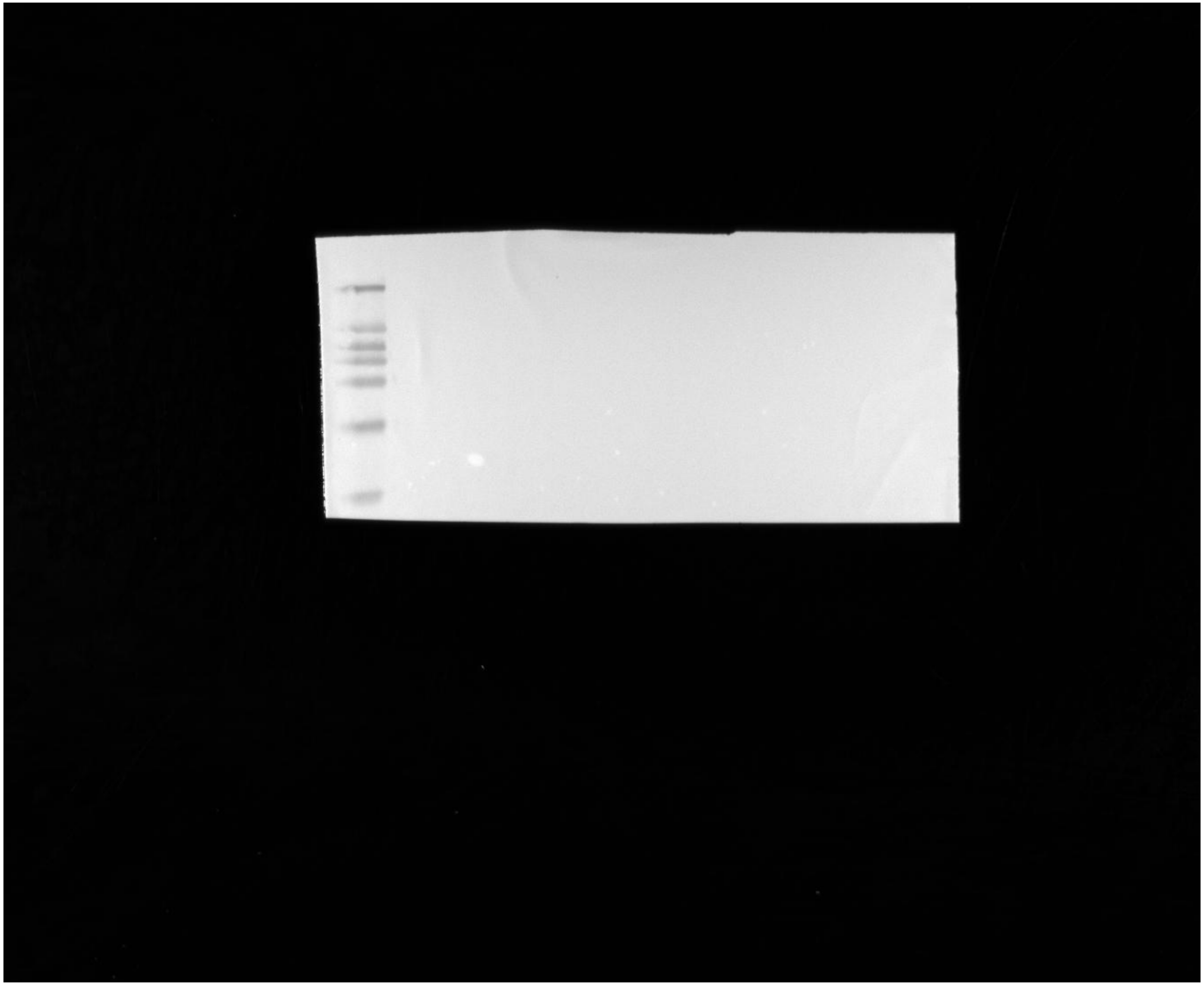

p-eIF2α PVDF image-1

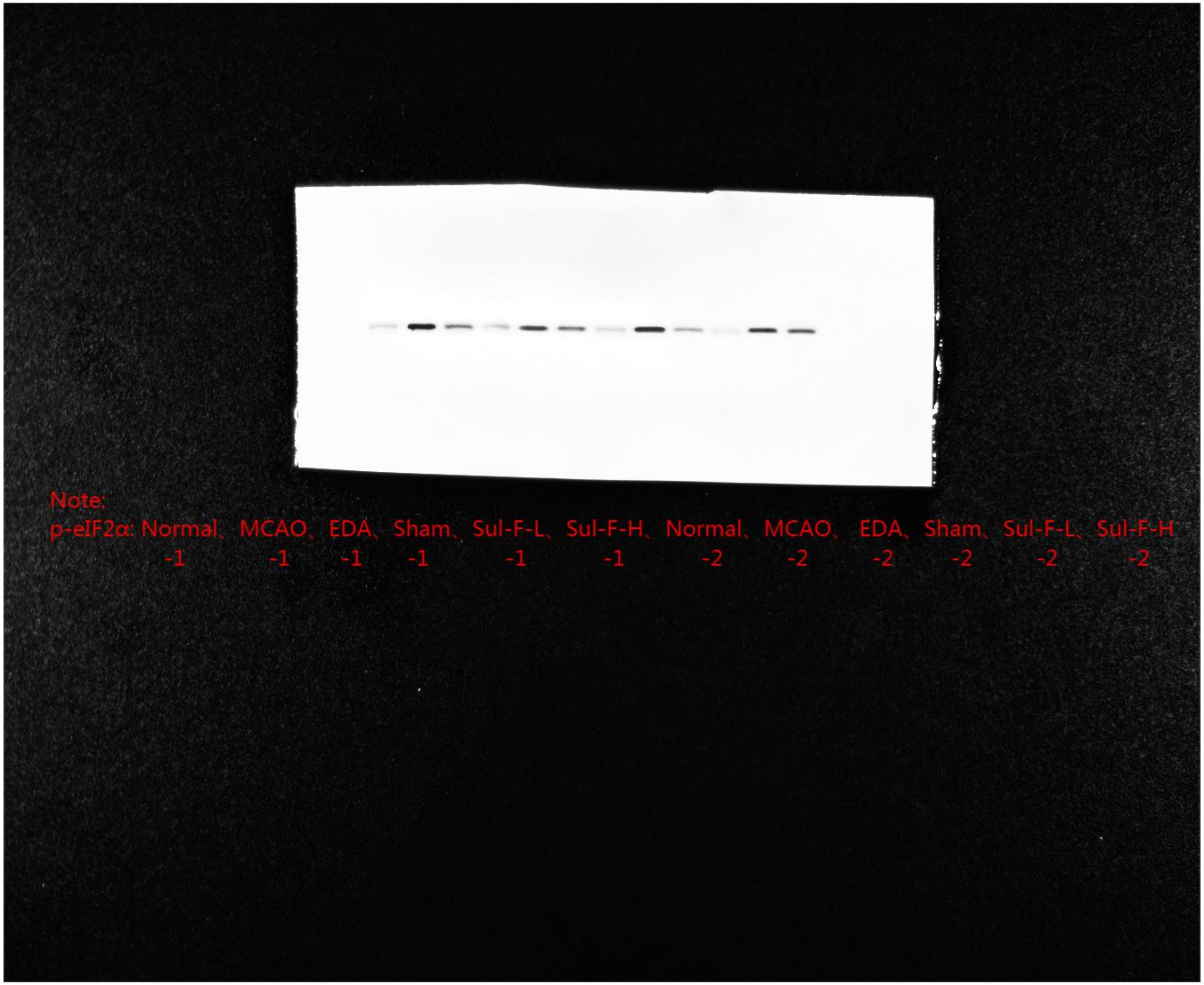

p-eIF2α Gel electrophoresis map-2

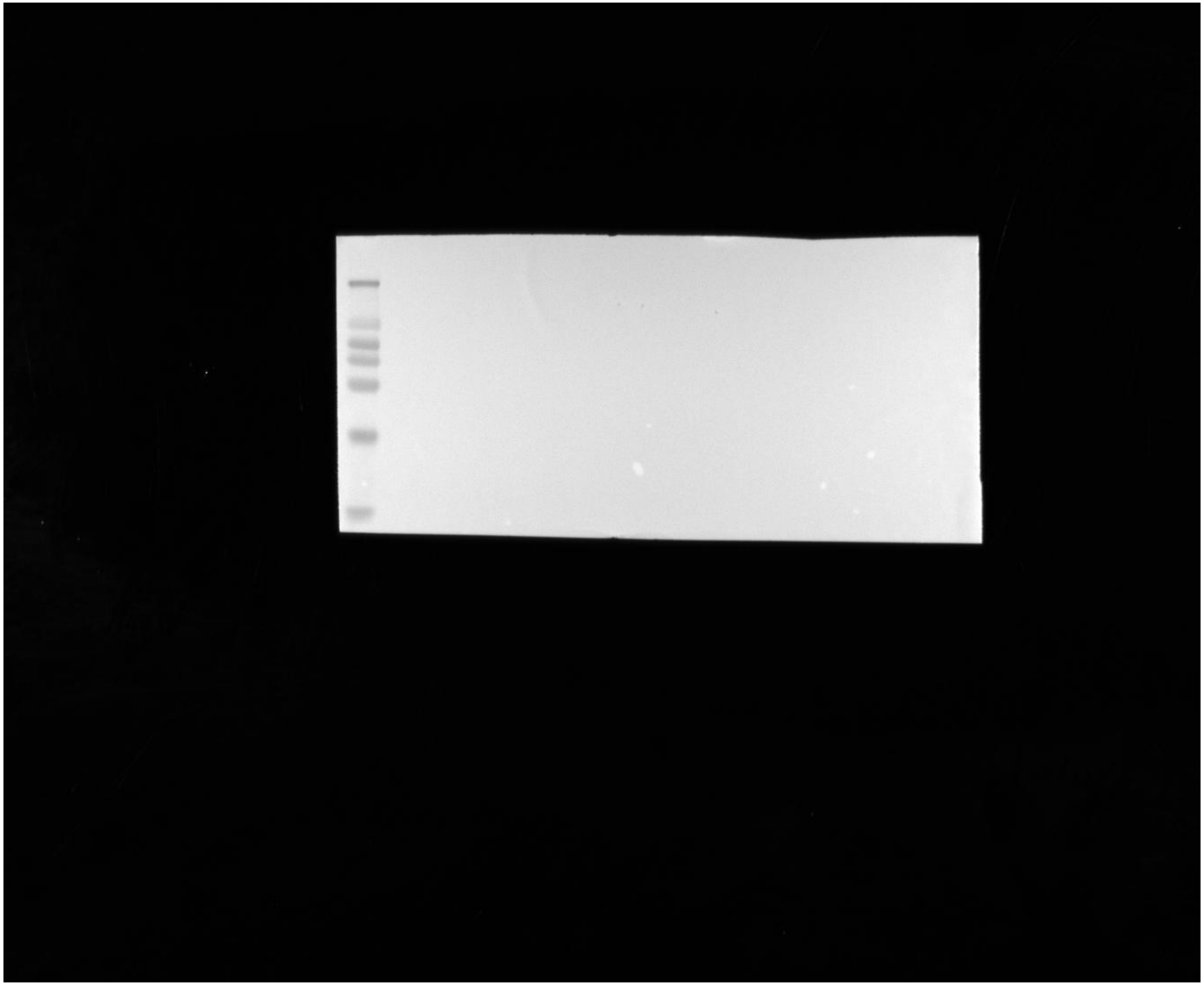

p-eIF2α PVDF image-2

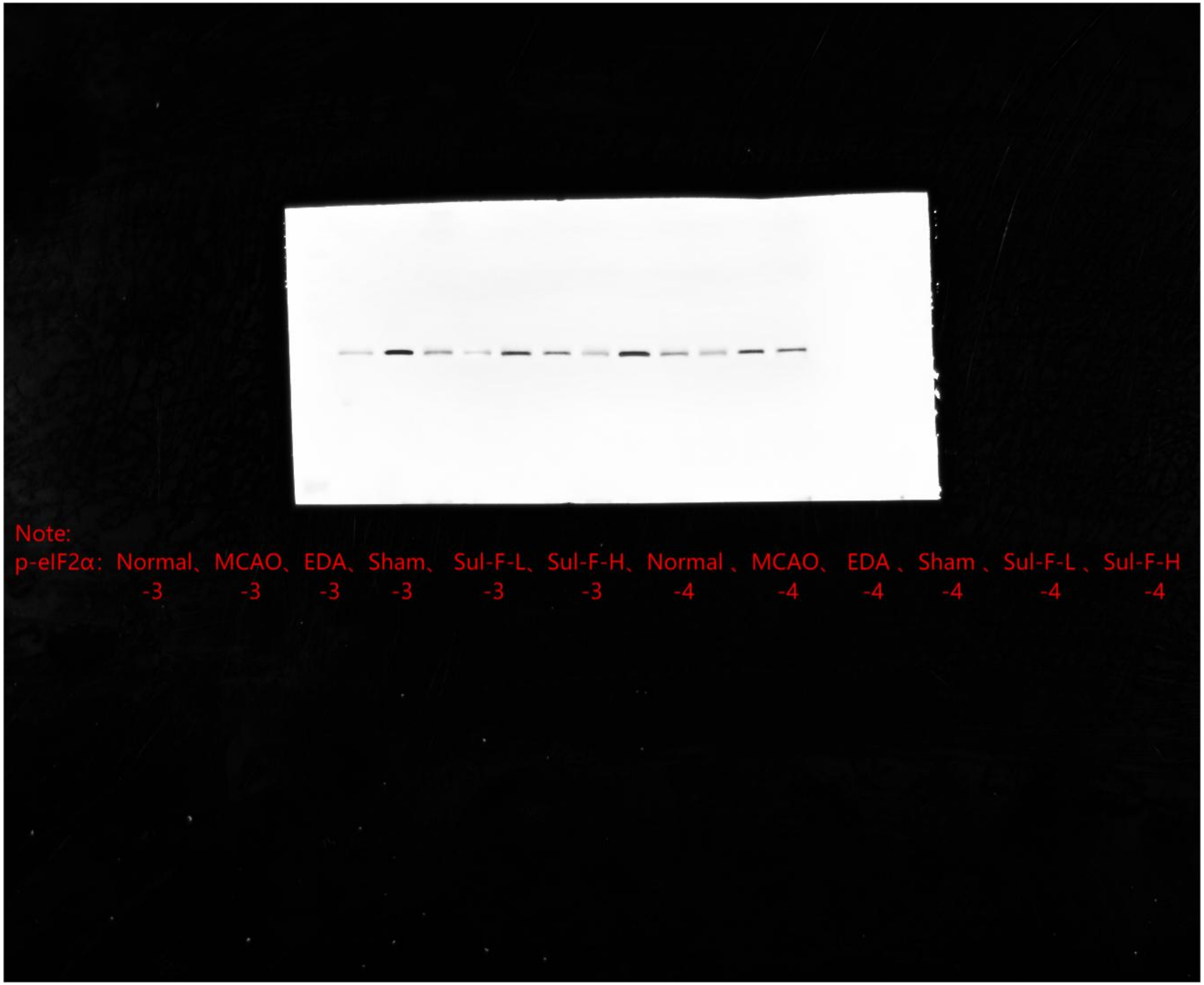

p-eIF2α Gel electrophoresis map-3

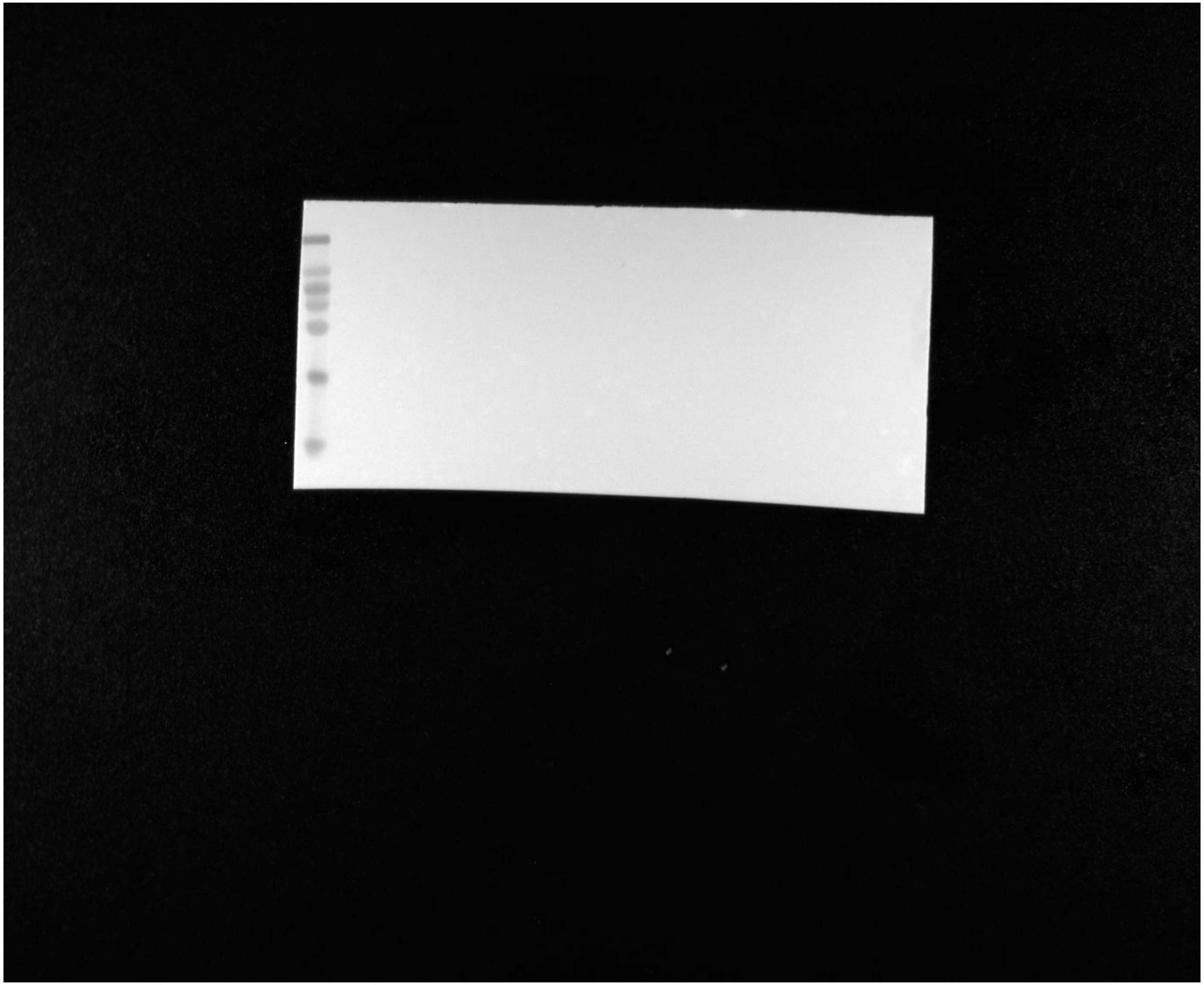

p-eIF2α PVDF image-3

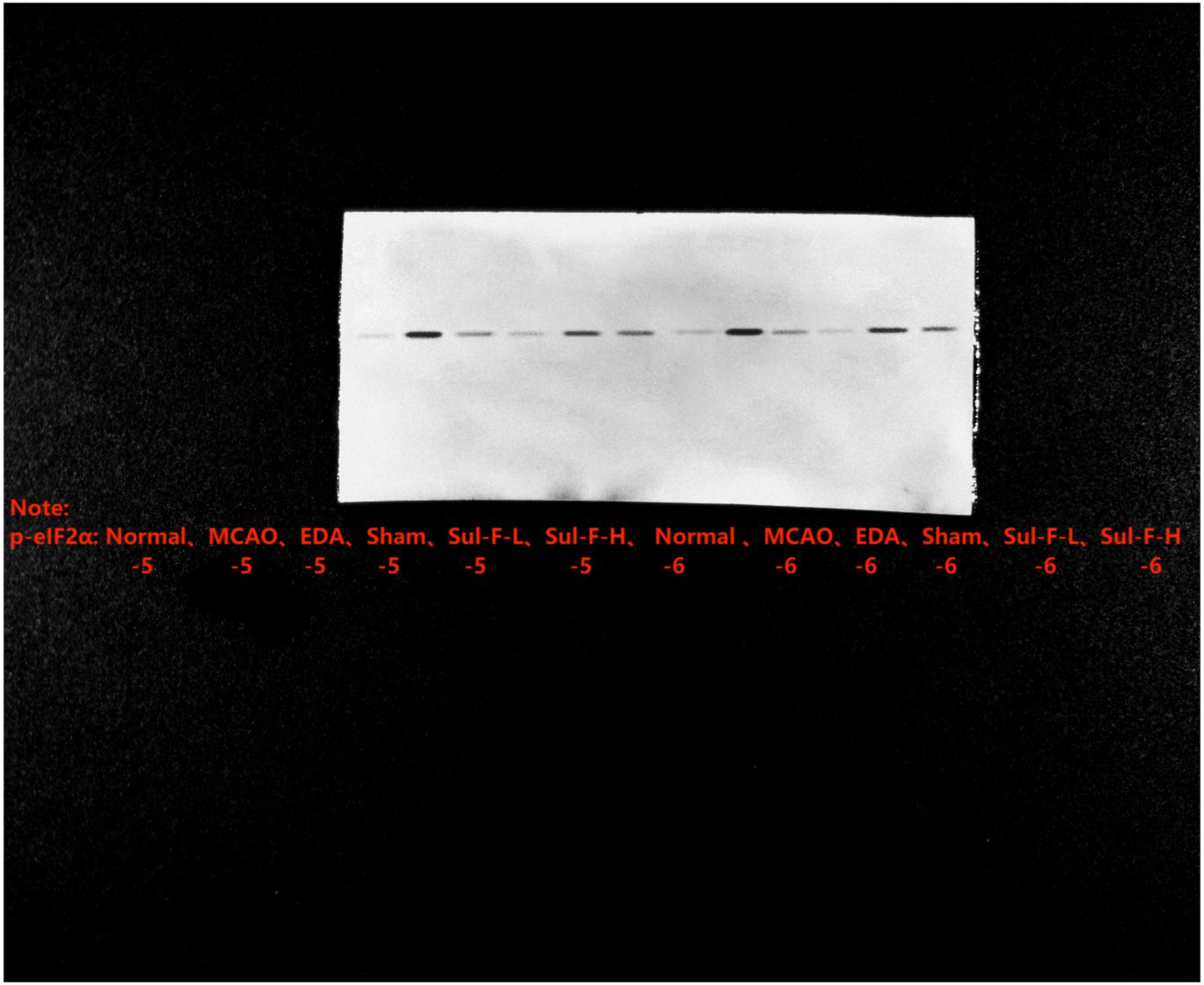

ATF4-1 Gel electrophoresis map

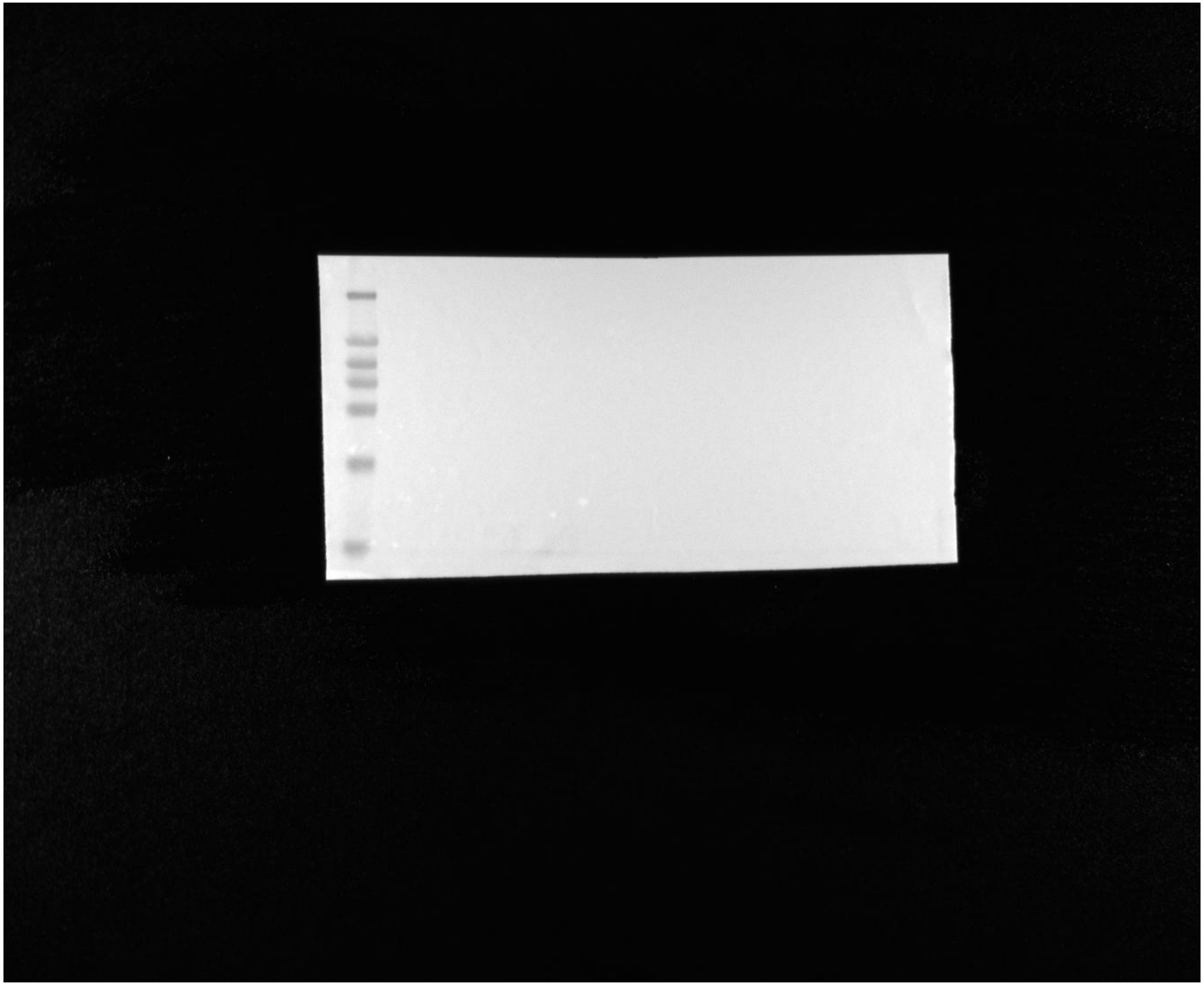

ATF4-1 PVDF image

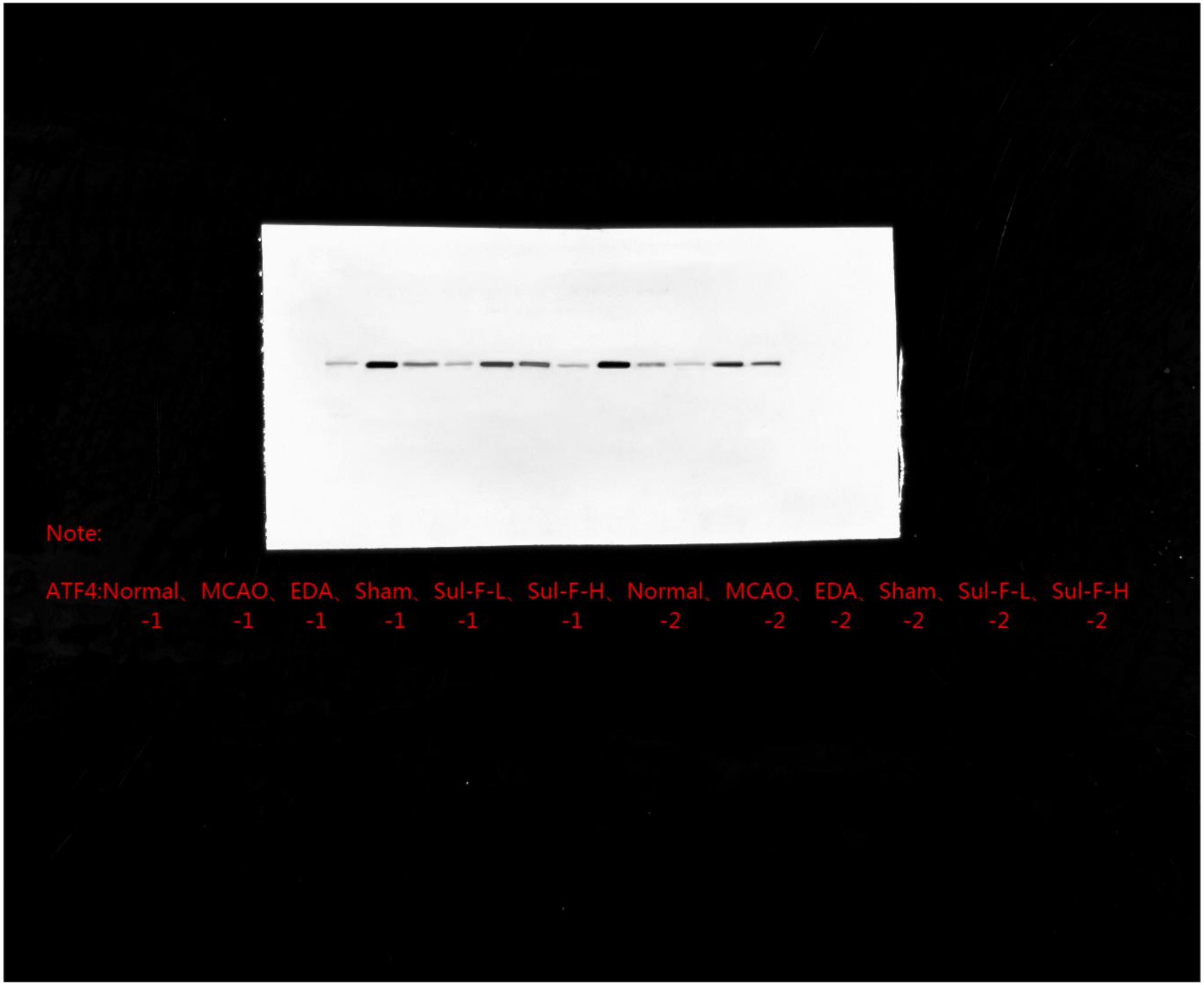

ATF4-2 Gel electrophoresis map

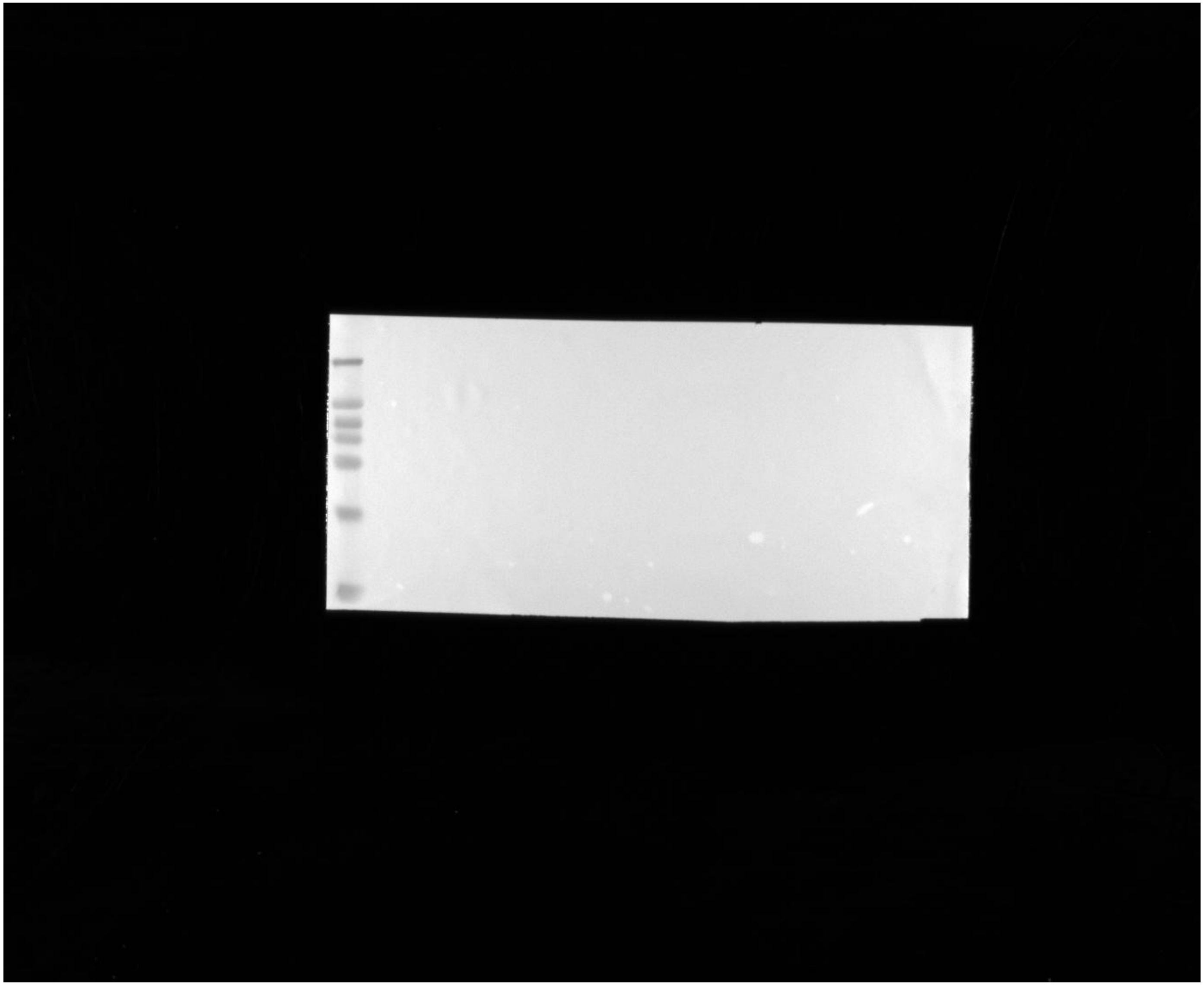

ATF4-2 PVDF image

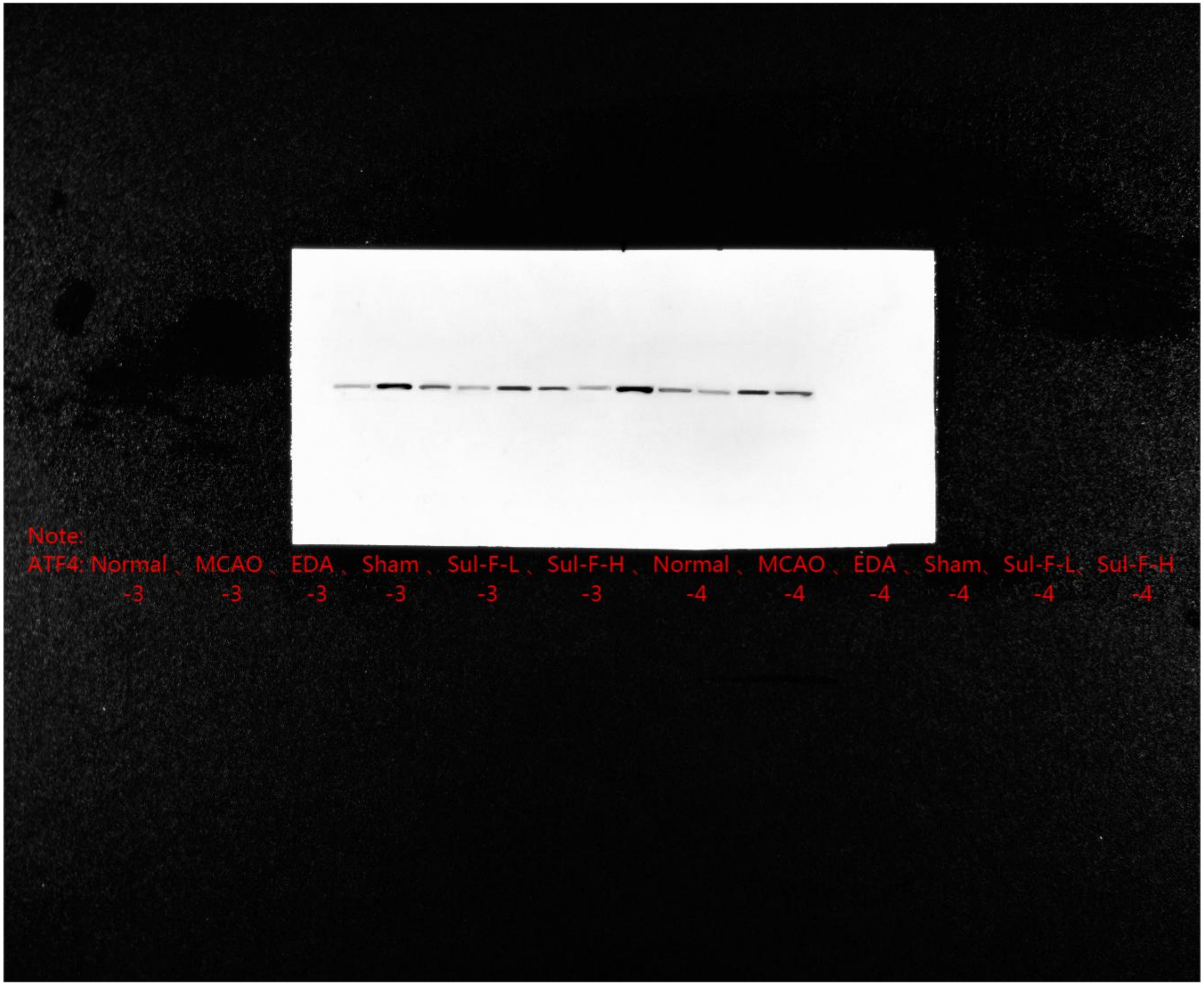

ATF4-3 Gel electrophoresis map

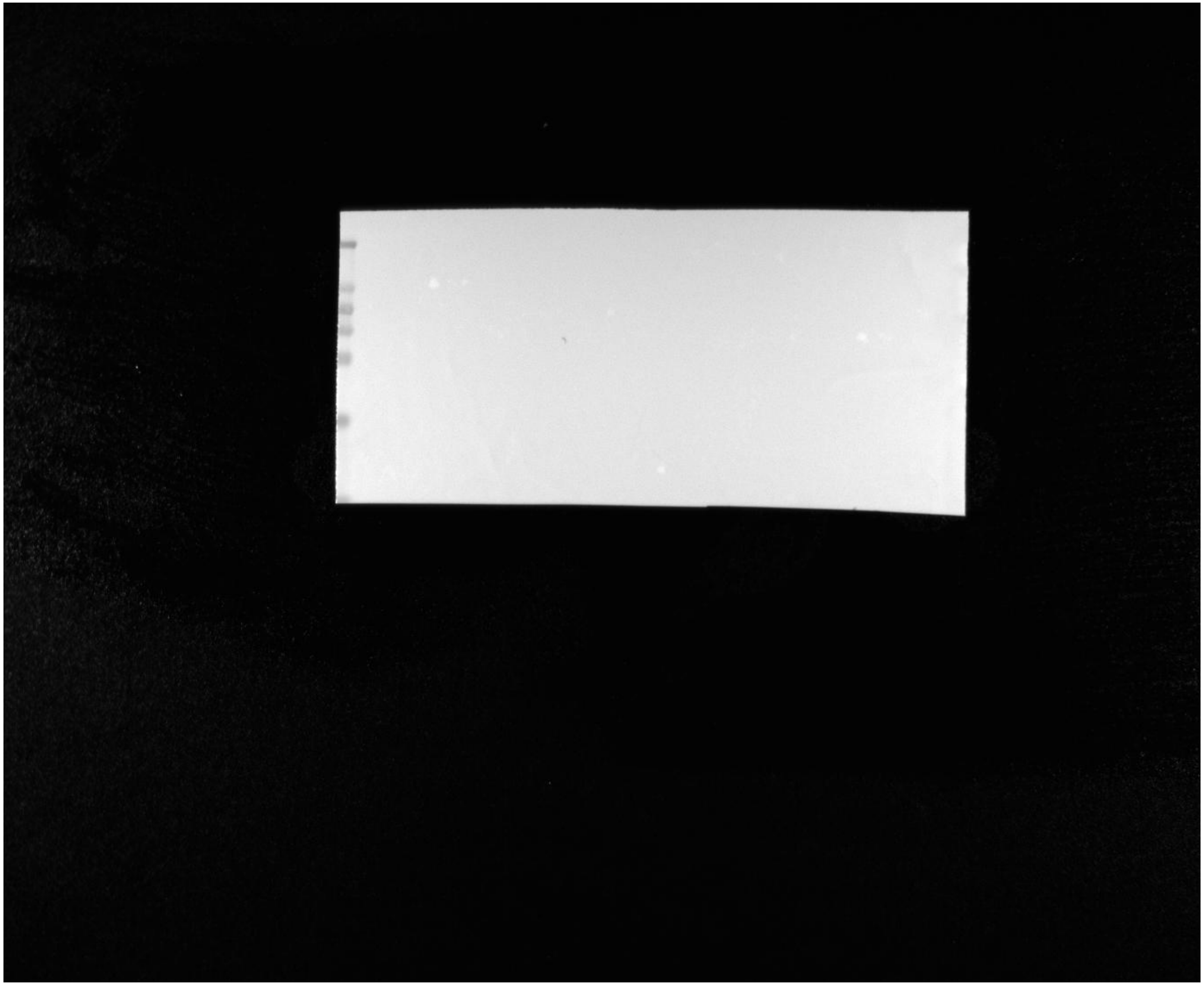

ATF4-3 PVDF image

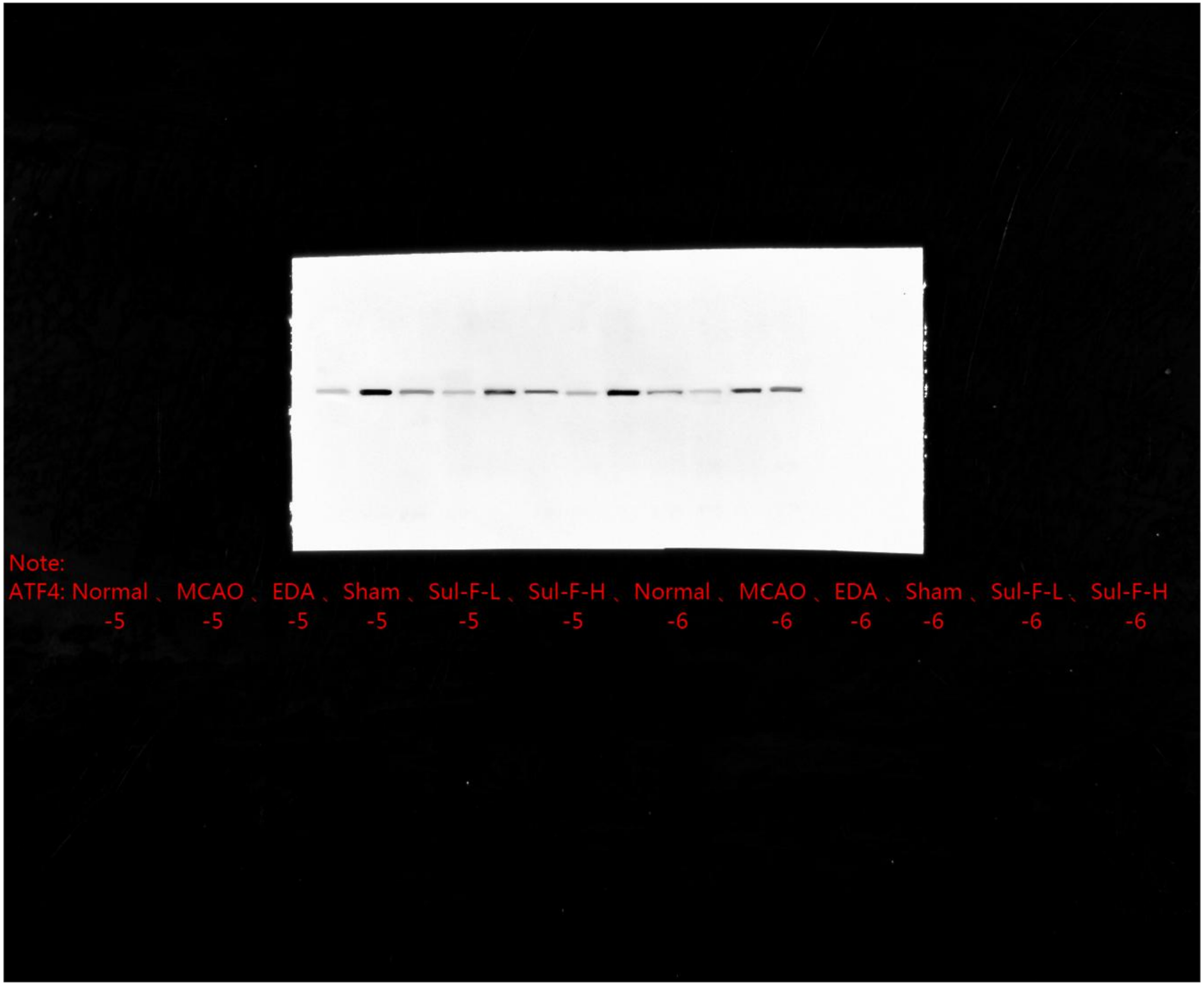

CHOP Gel electrophoresis map-1

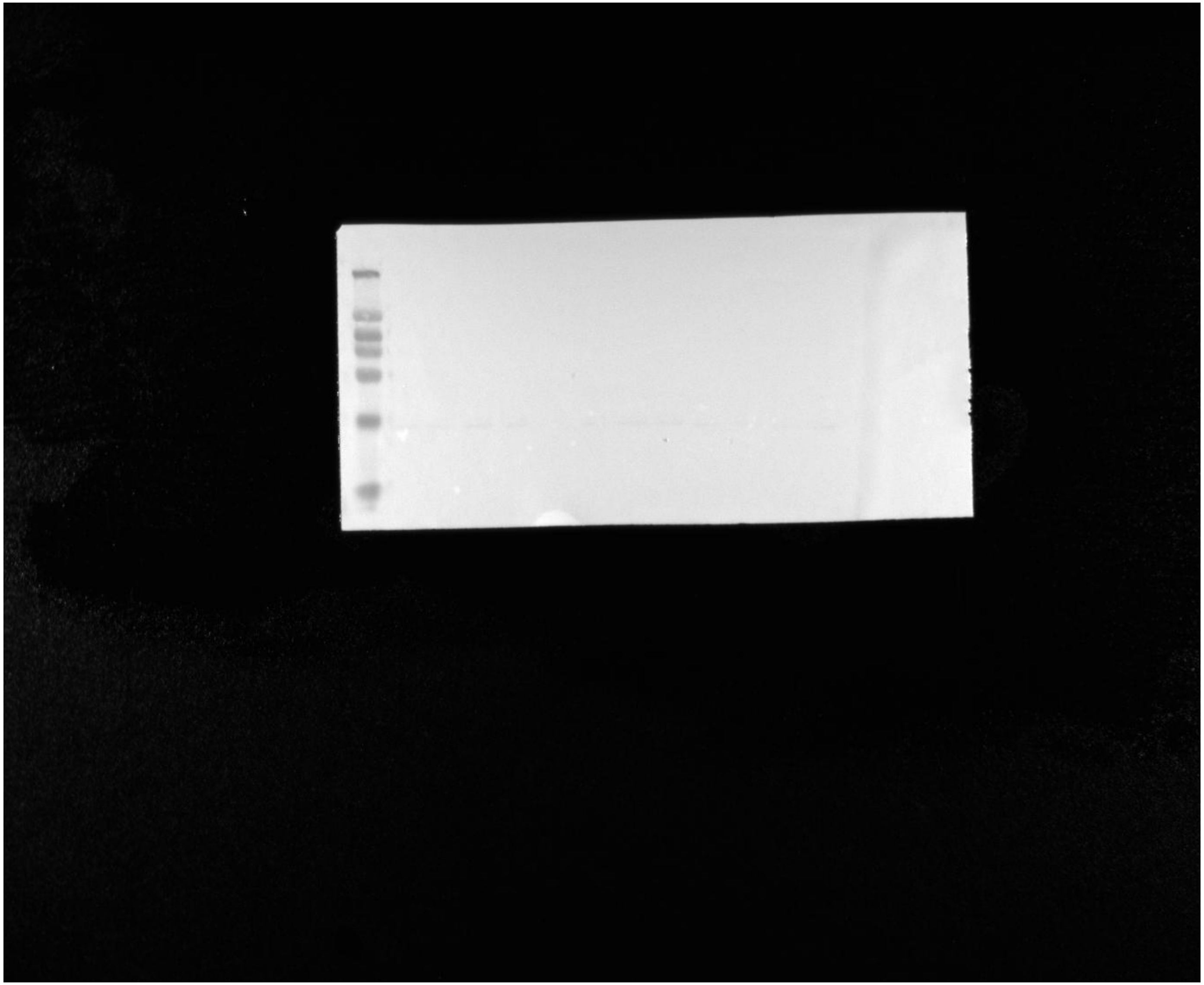

CHOP PVDF image-1

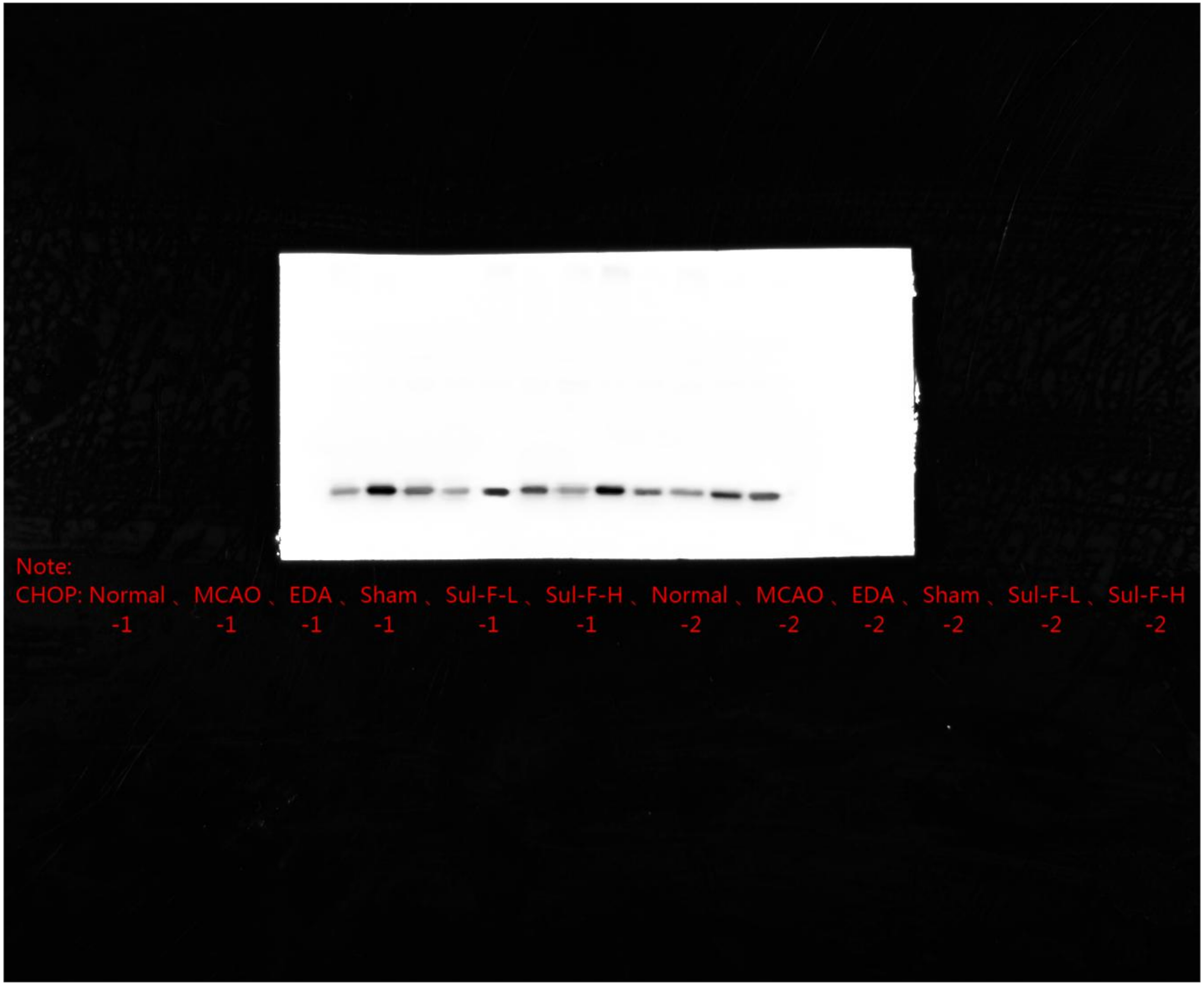

CHOP Gel electrophoresis map-2

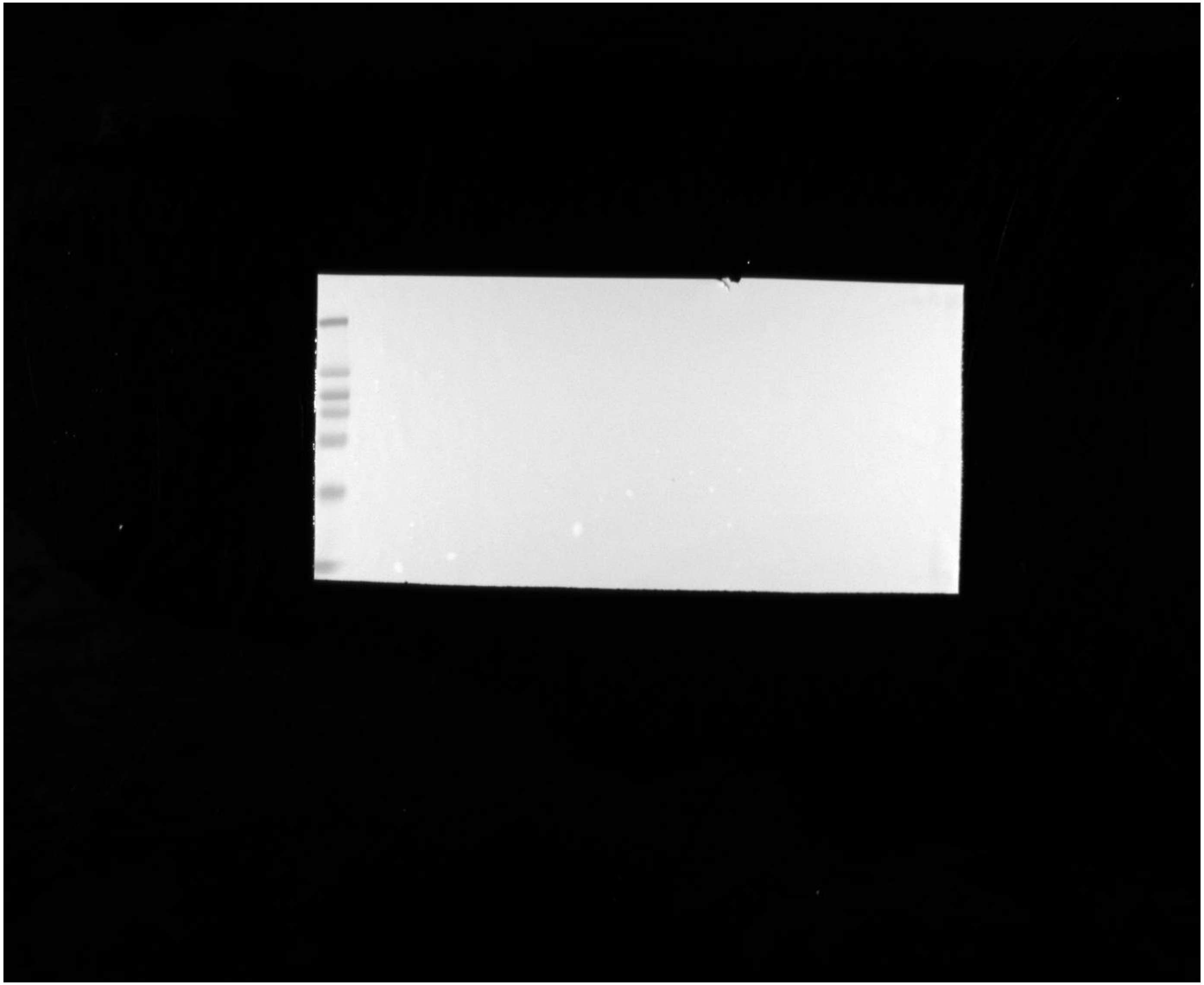

CHOP PVDF image-2

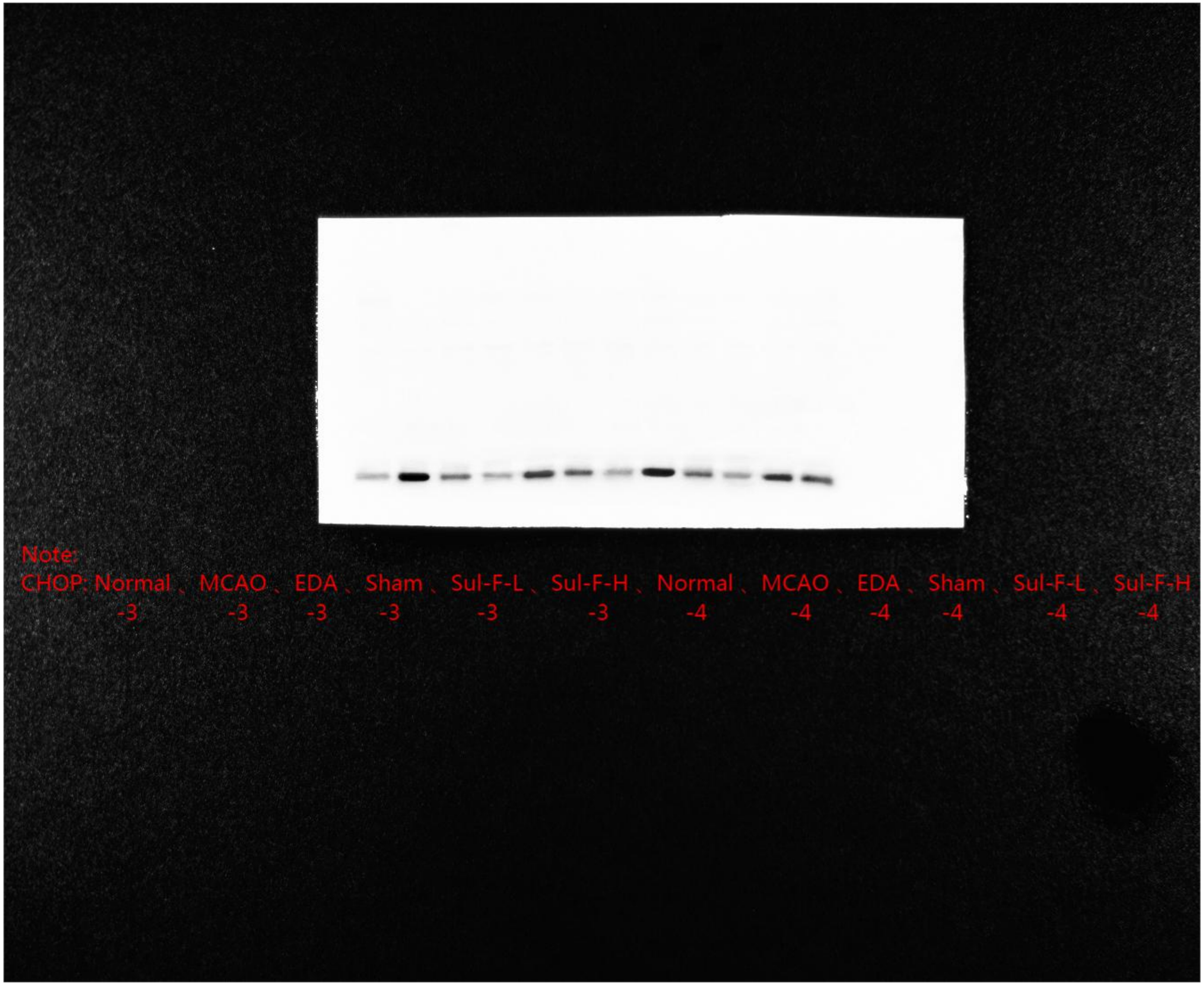

CHOP Gel electrophoresis map-3

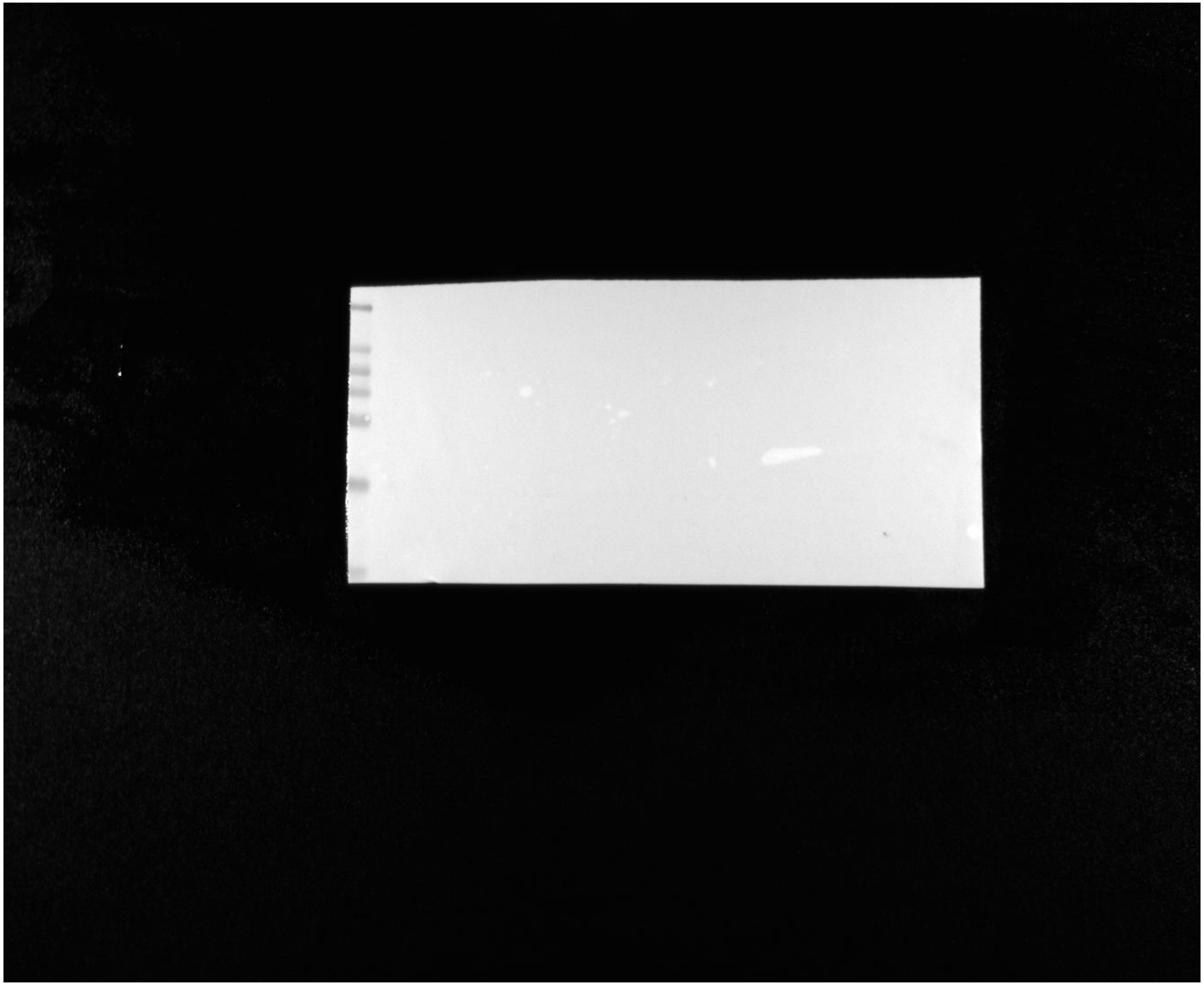

CHOP PVDF image-3

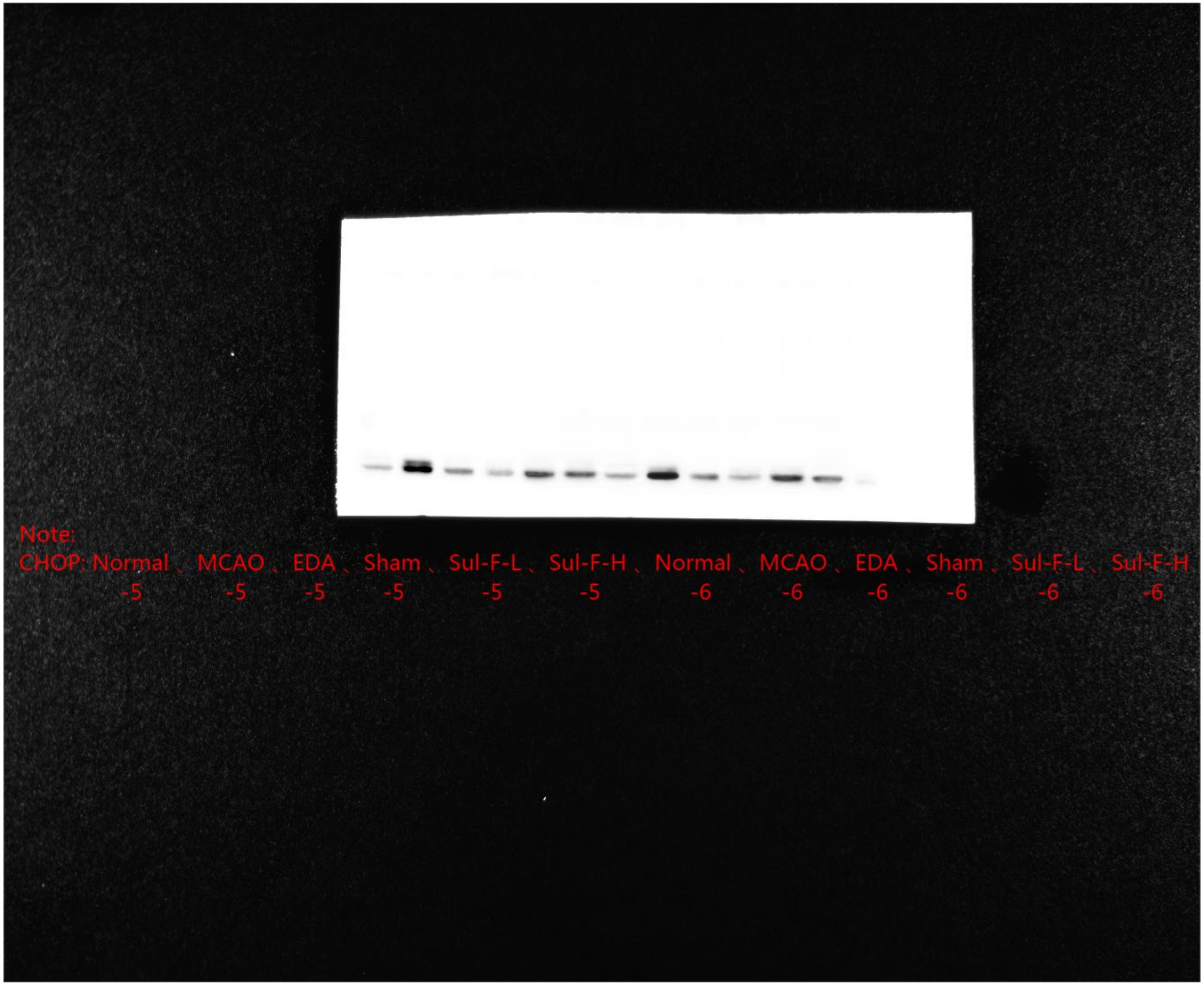

Bax Gel electrophoresis map 1

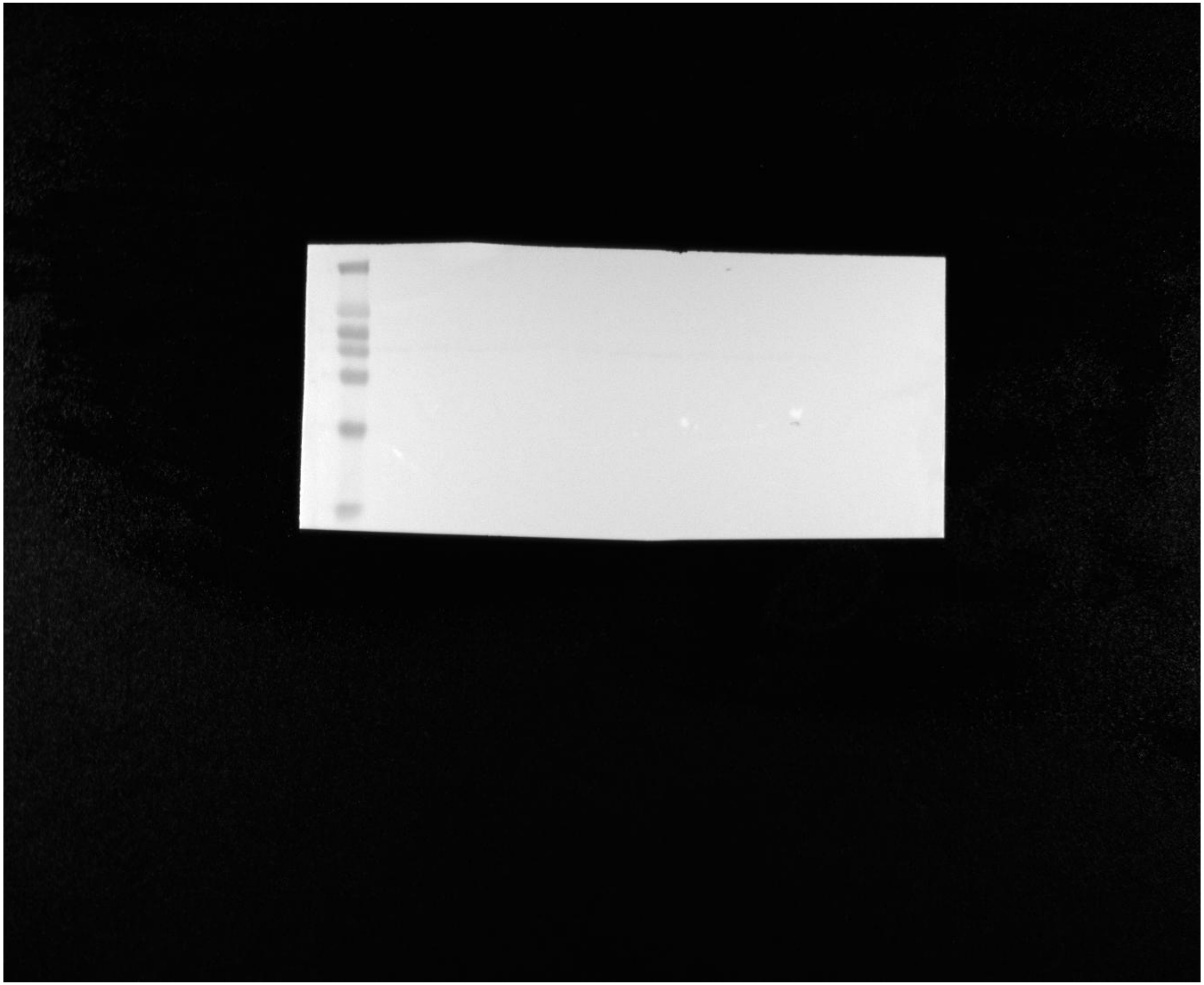

Bax PVDF image-1

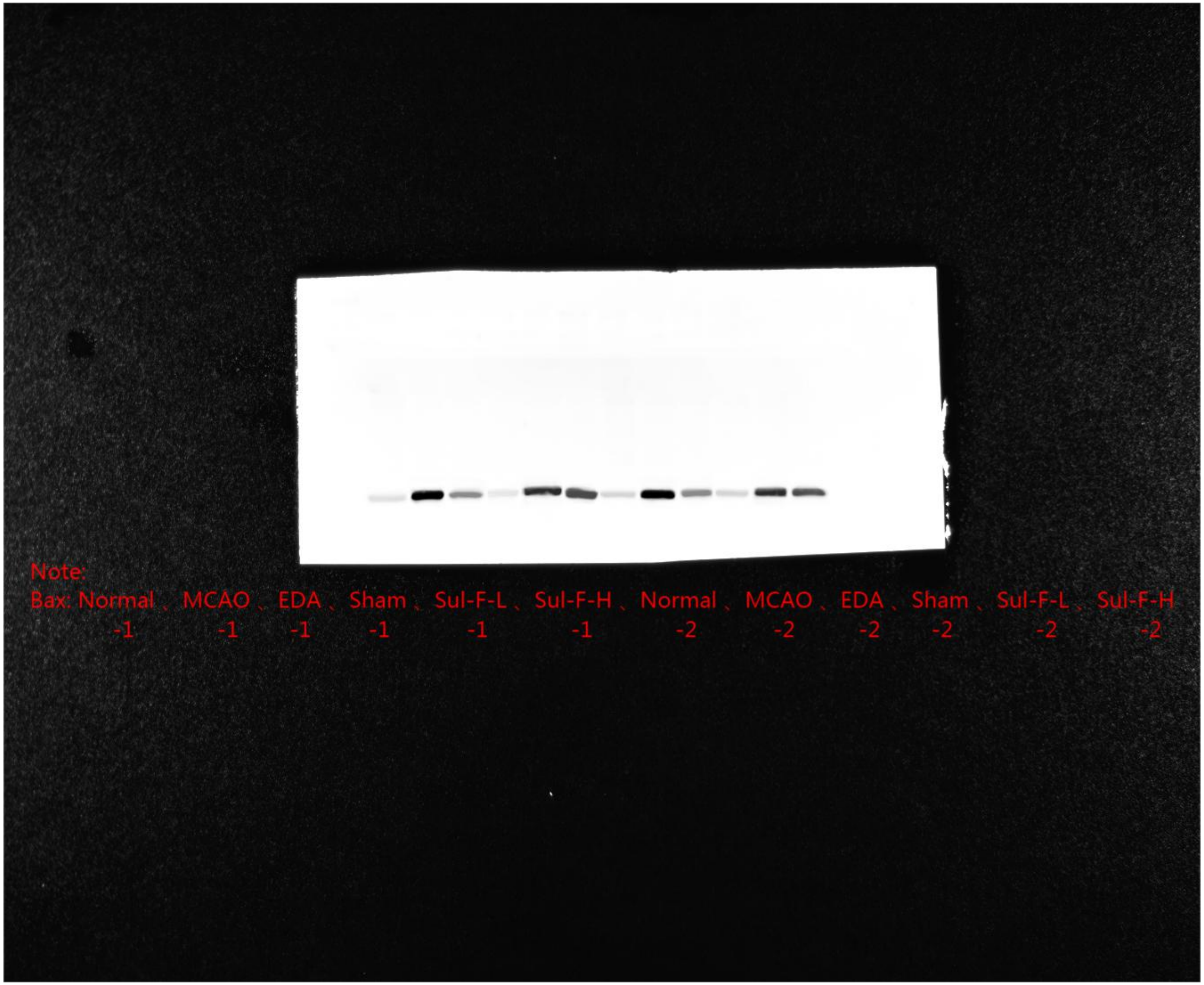

Bax Gel electrophoresis map-2

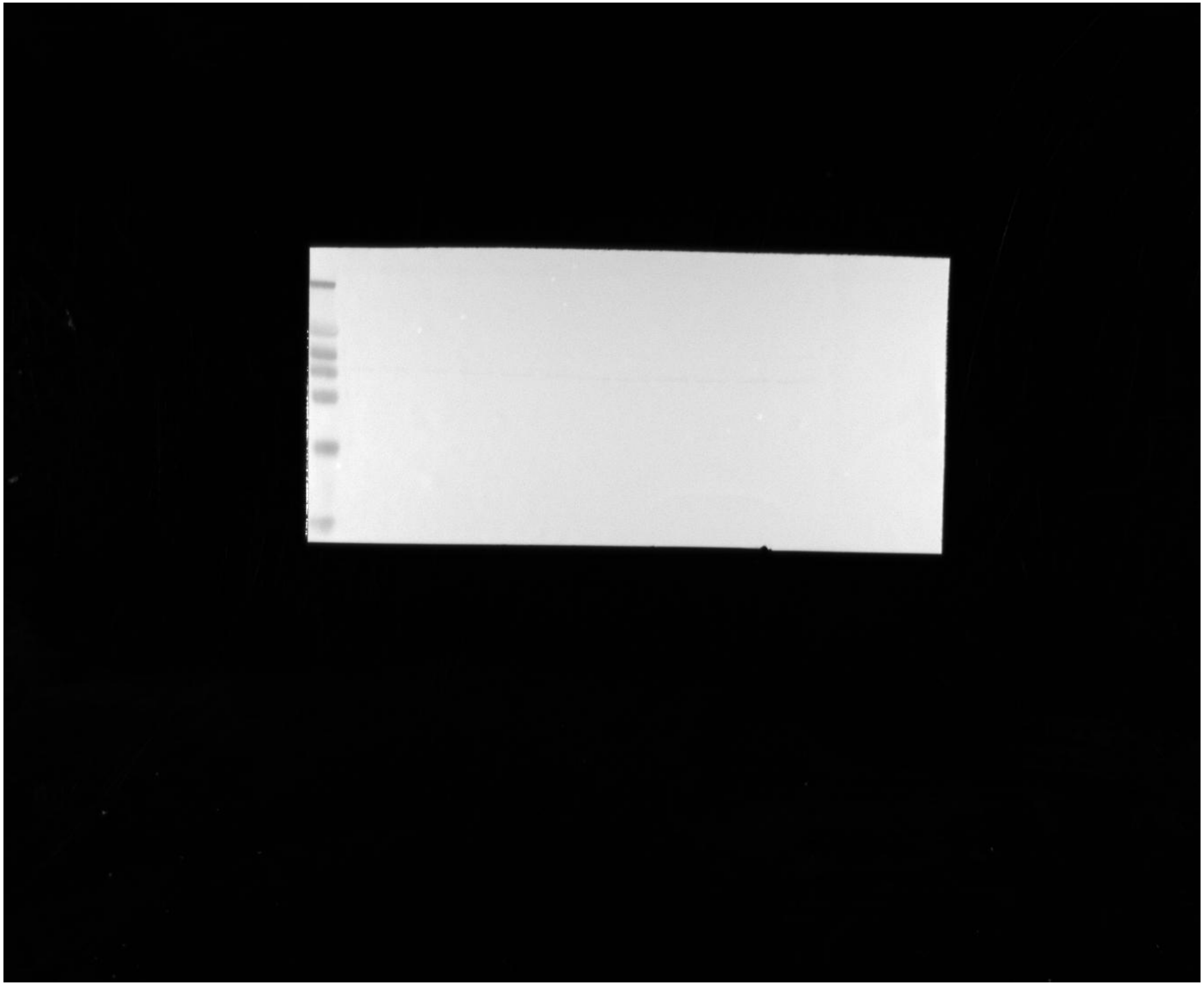

Bax PVDF image-2

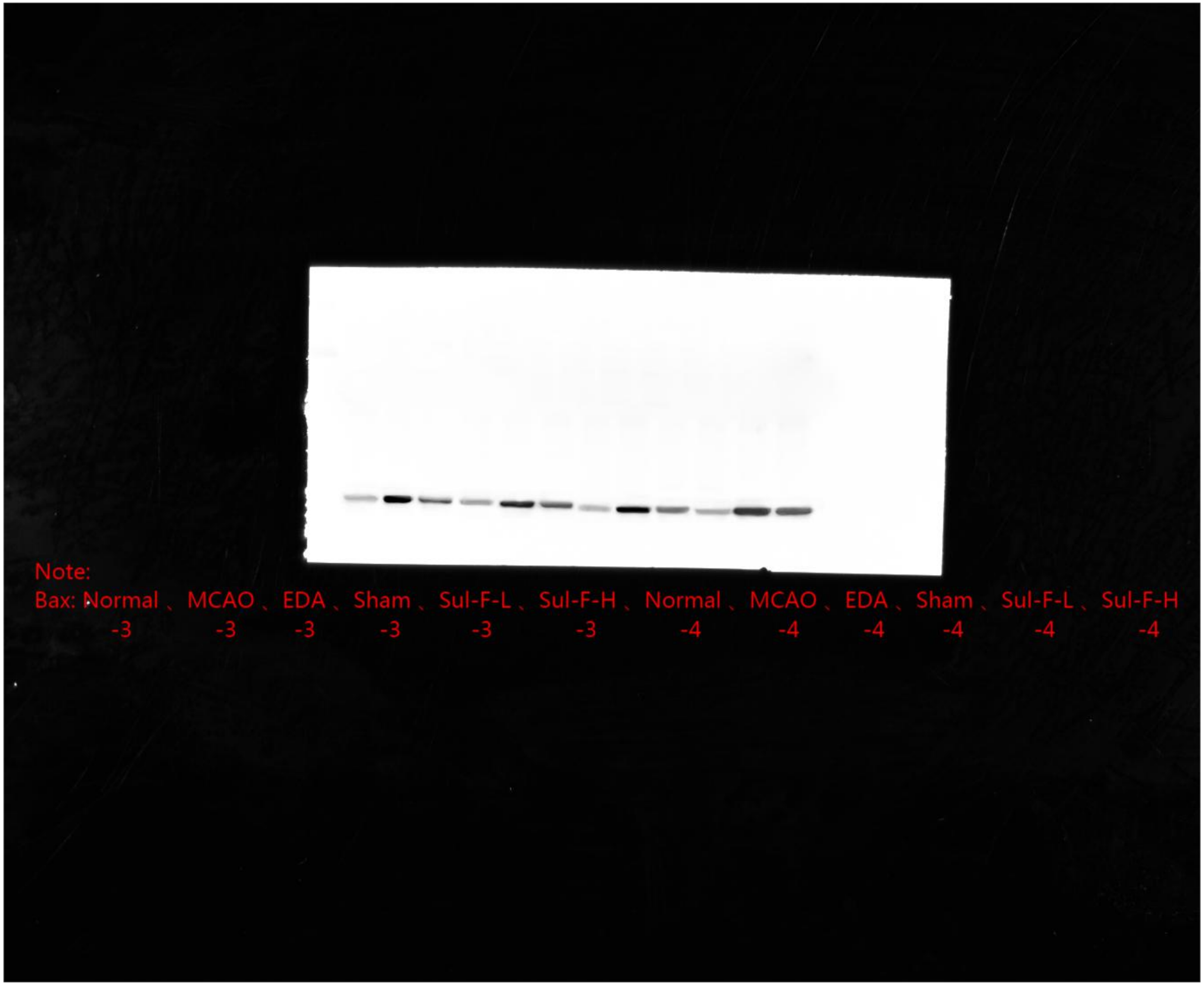

Bax Gel electrophoresis map-3

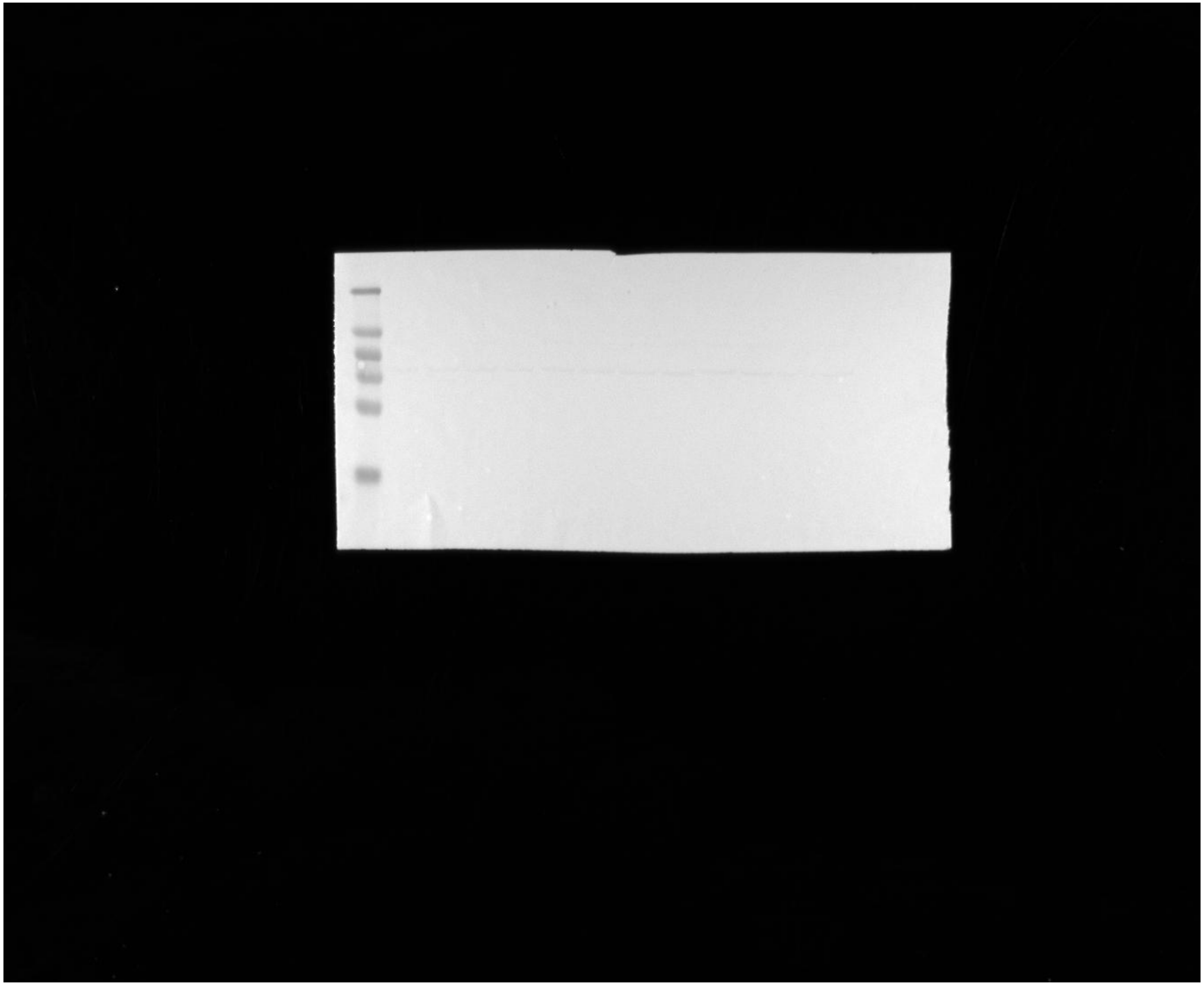

Bax PVDF image-3

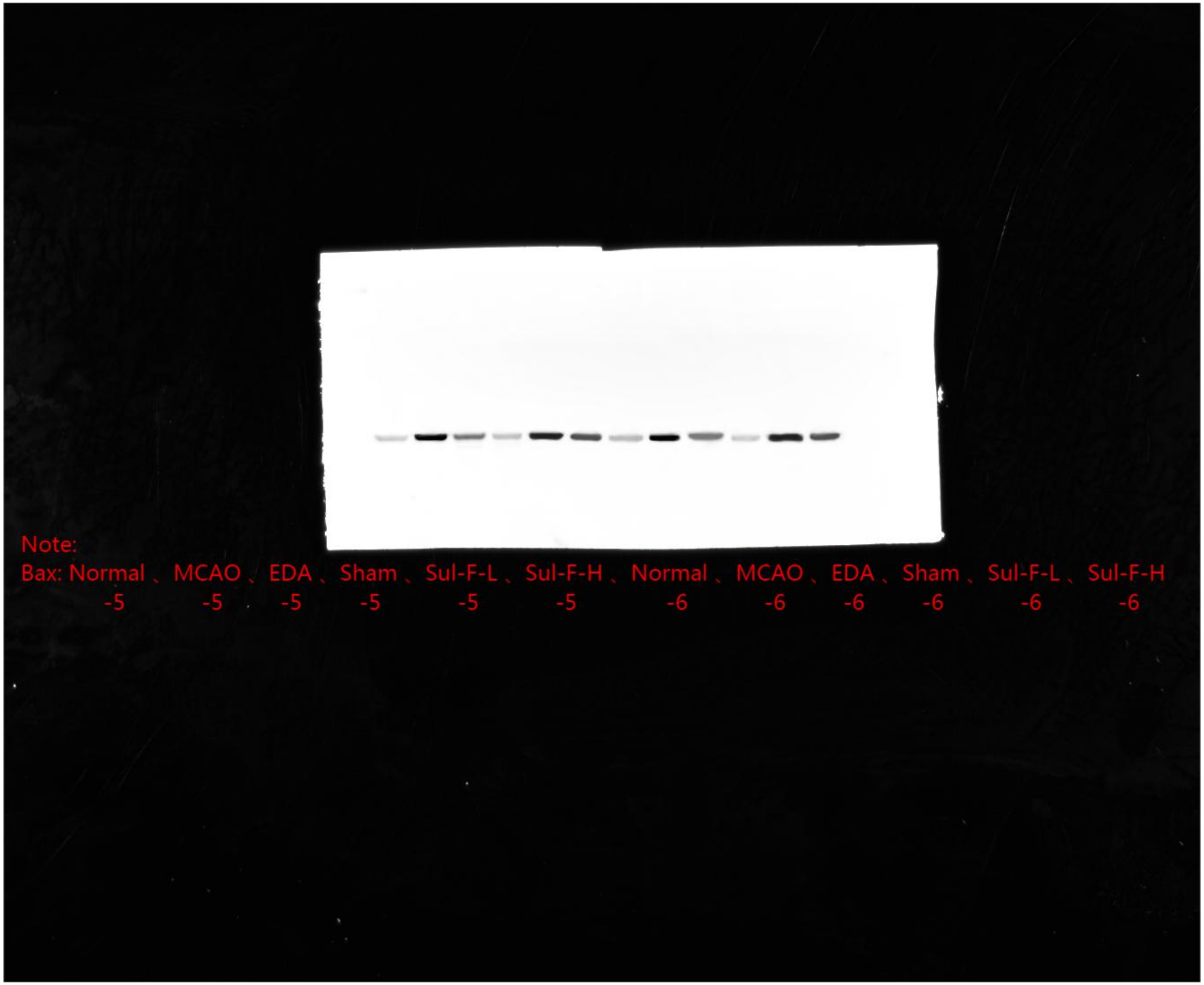

Bcl-2 Gel electrophoresis map-1

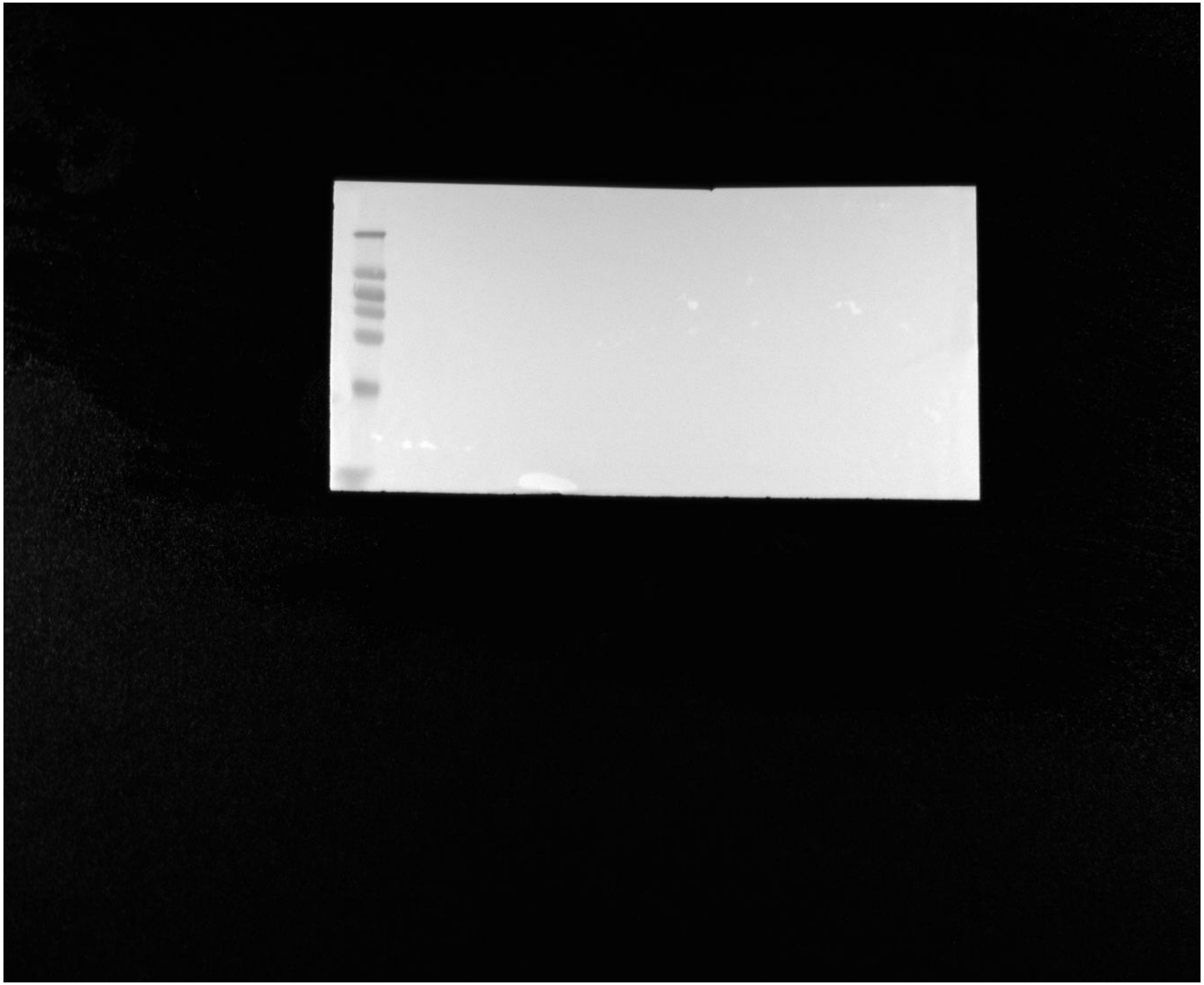

Bcl-2 PVDF image-1

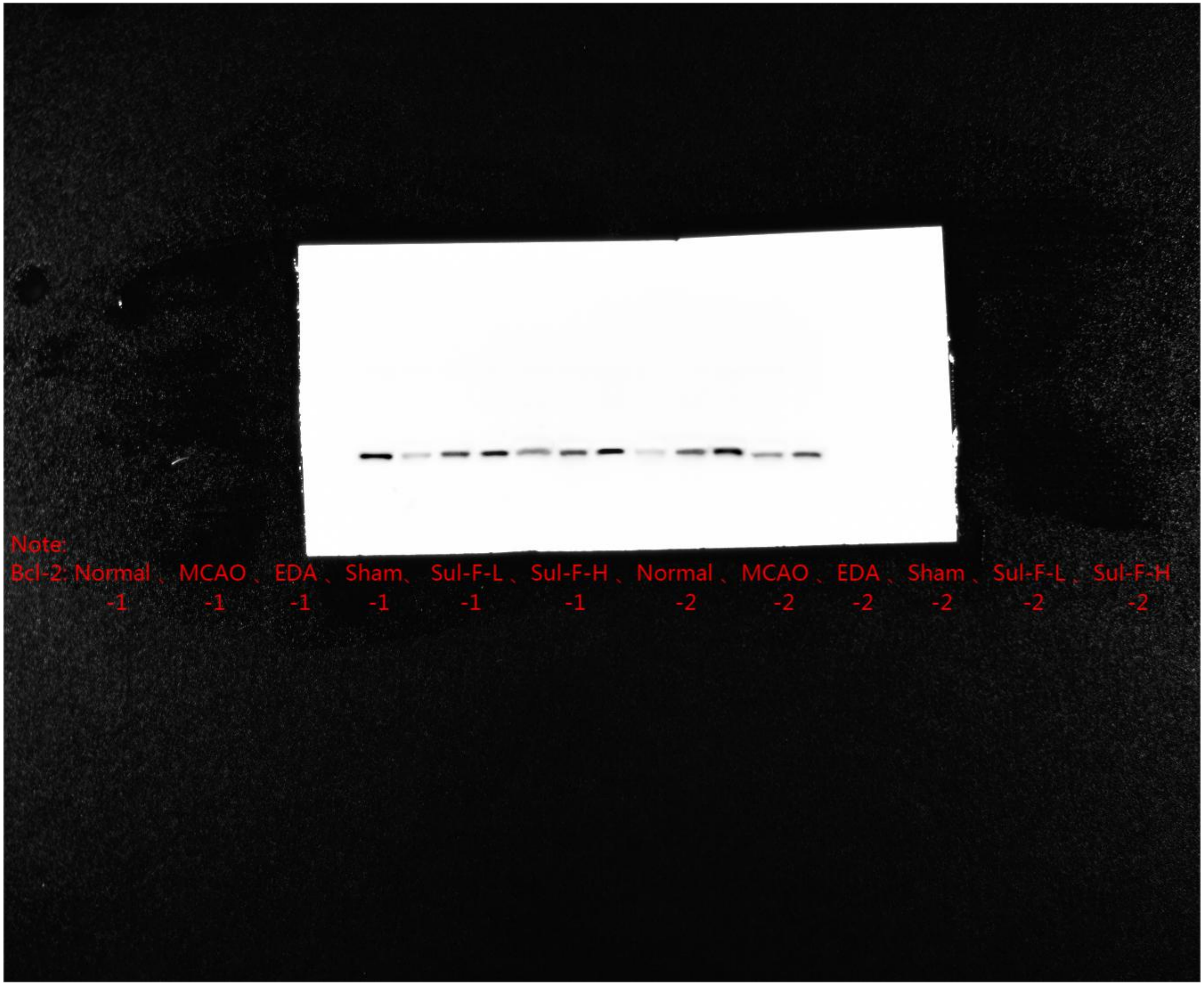

Bcl-2 Gel electrophoresis map-2

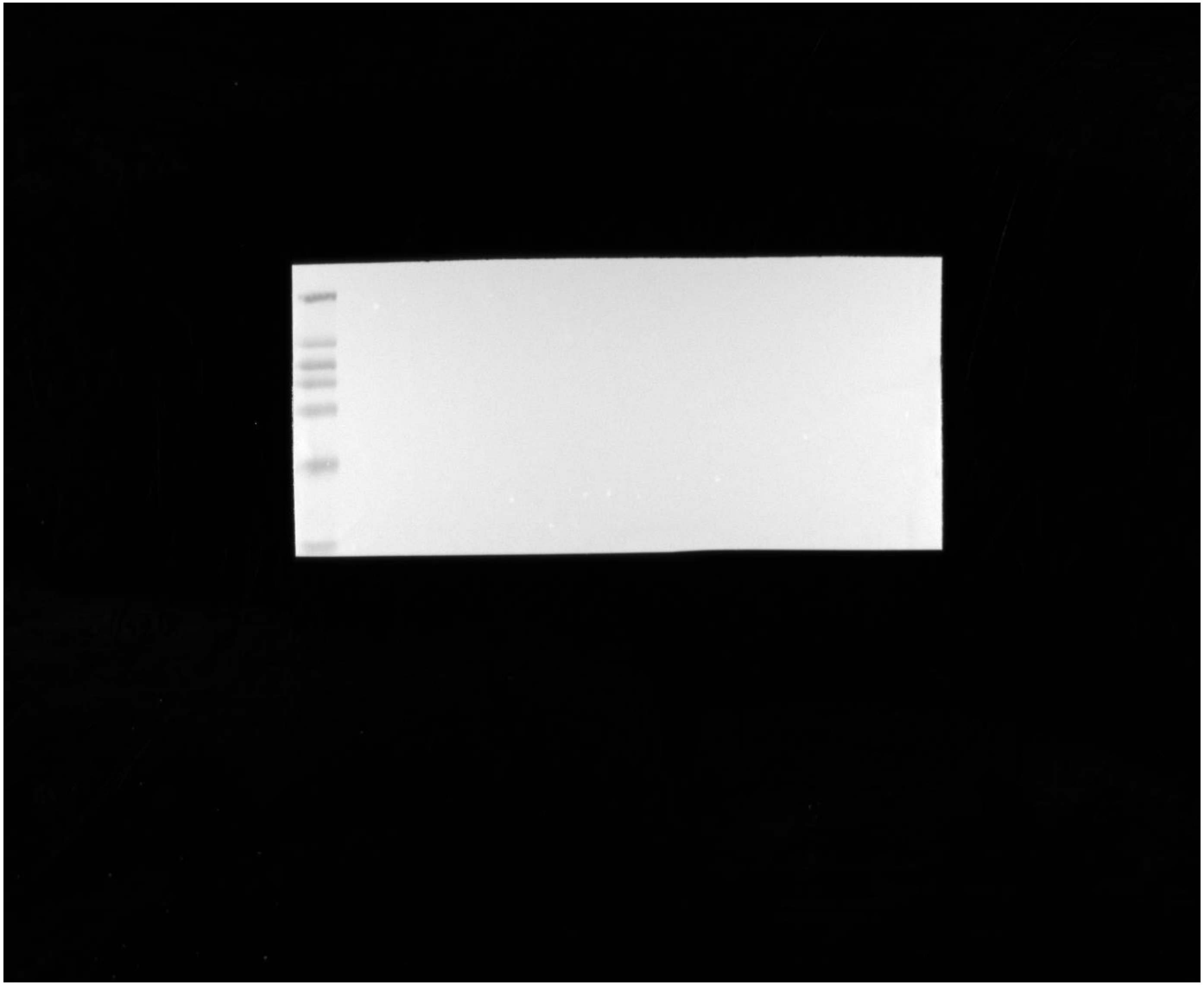

Bcl-2 PVDF image-2

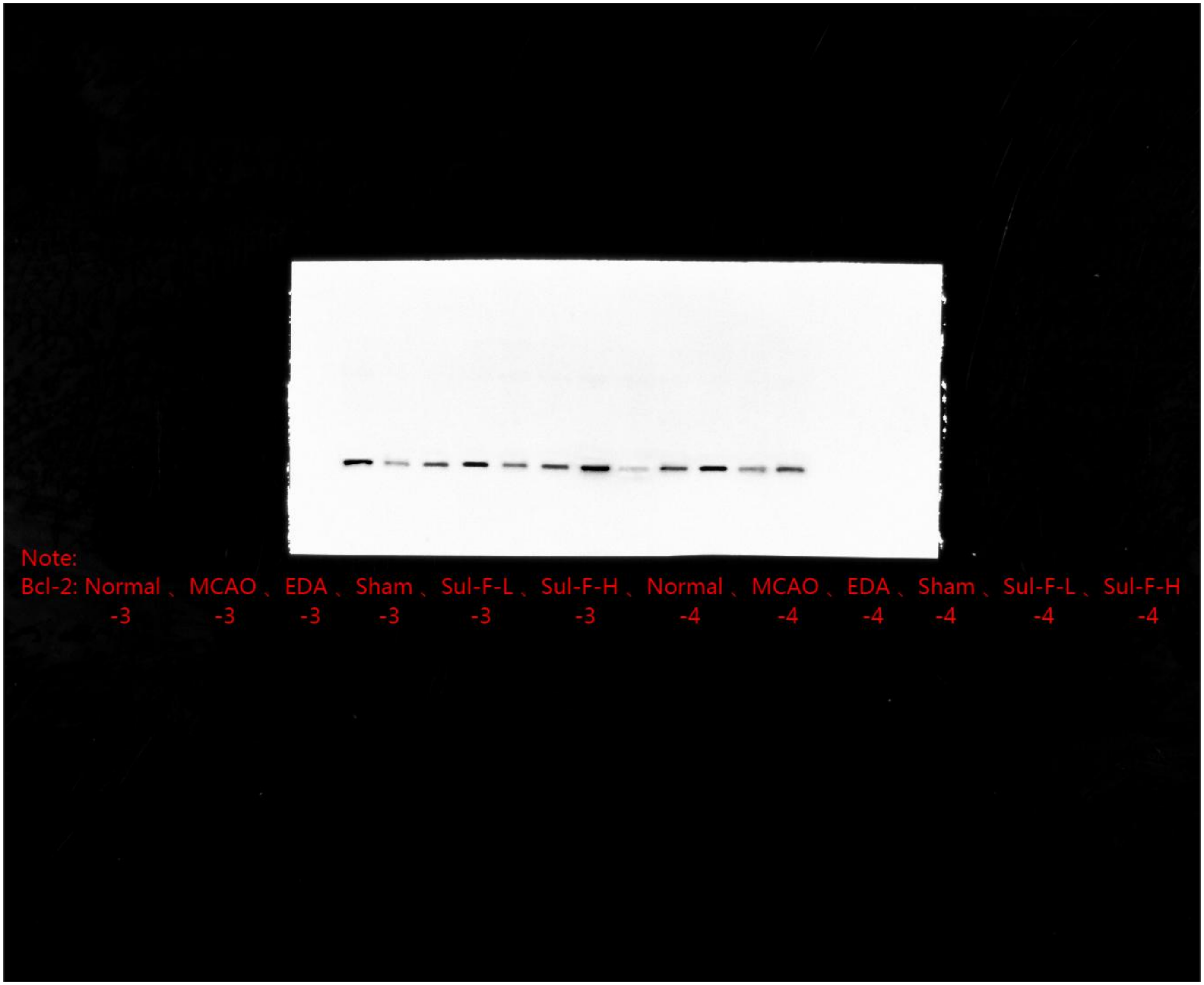

Bcl-2 Gel electrophoresis map-3

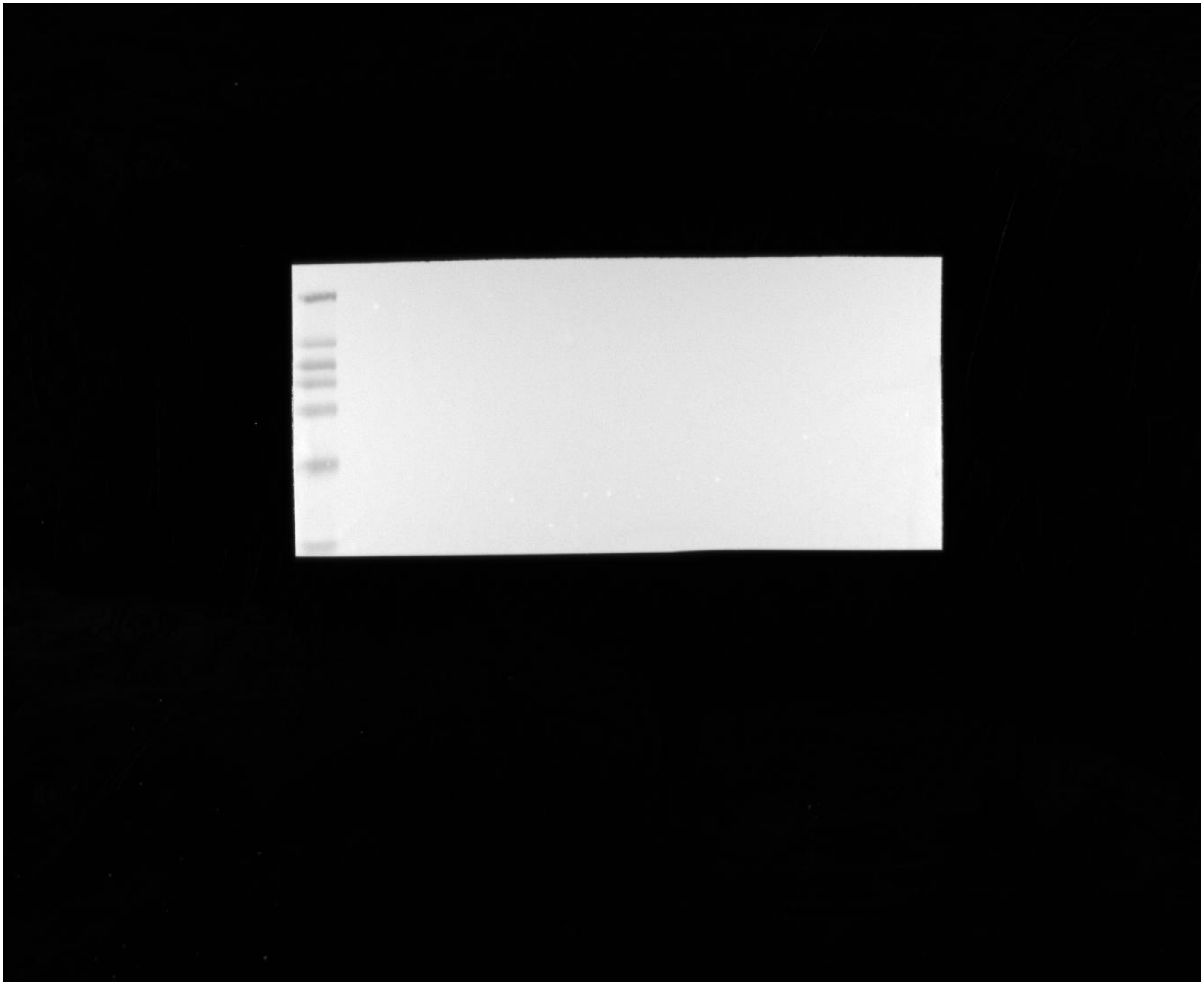

Bcl-2 PVDF image-3

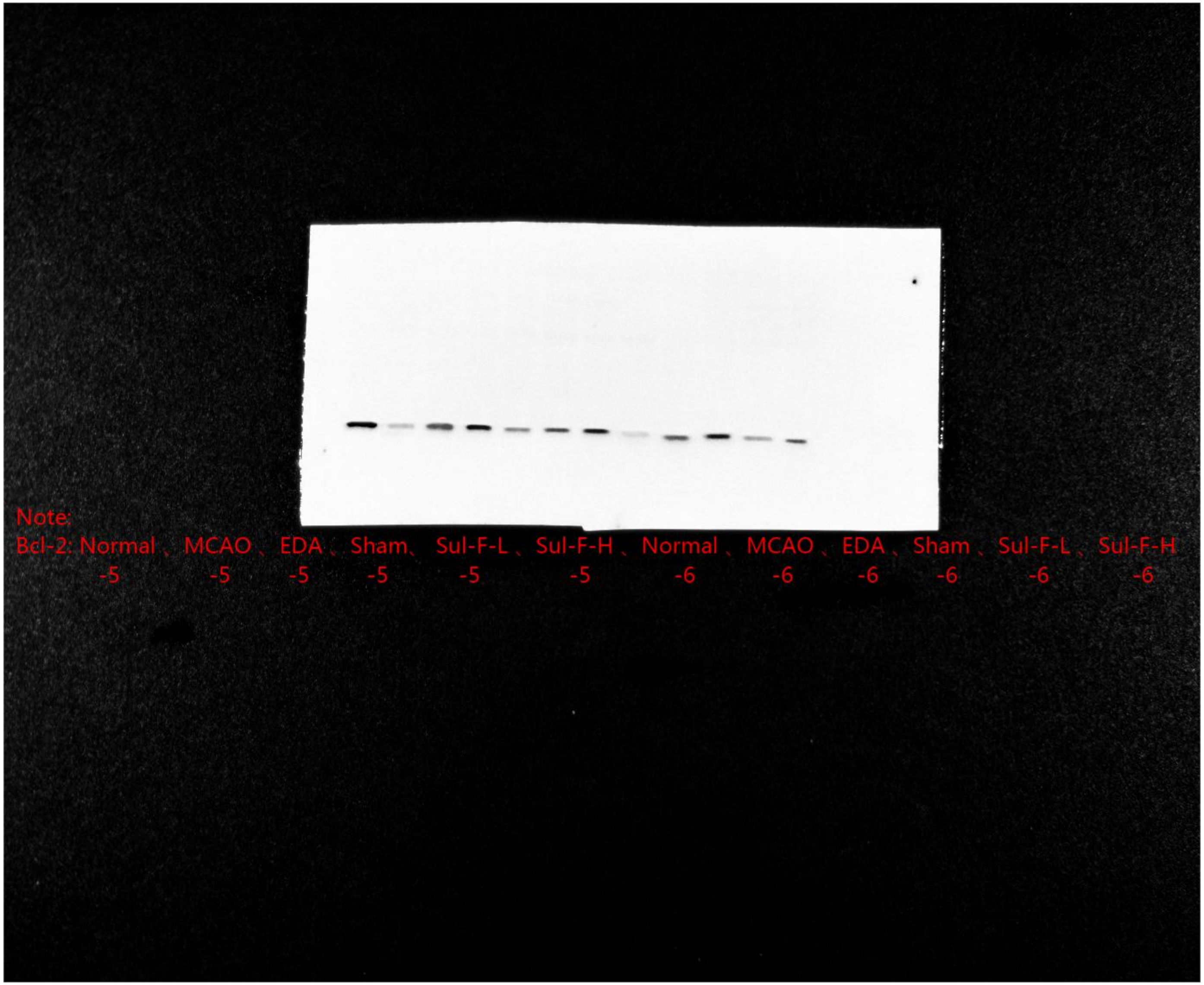

p-IRE1 Gel electrophoresis map-1

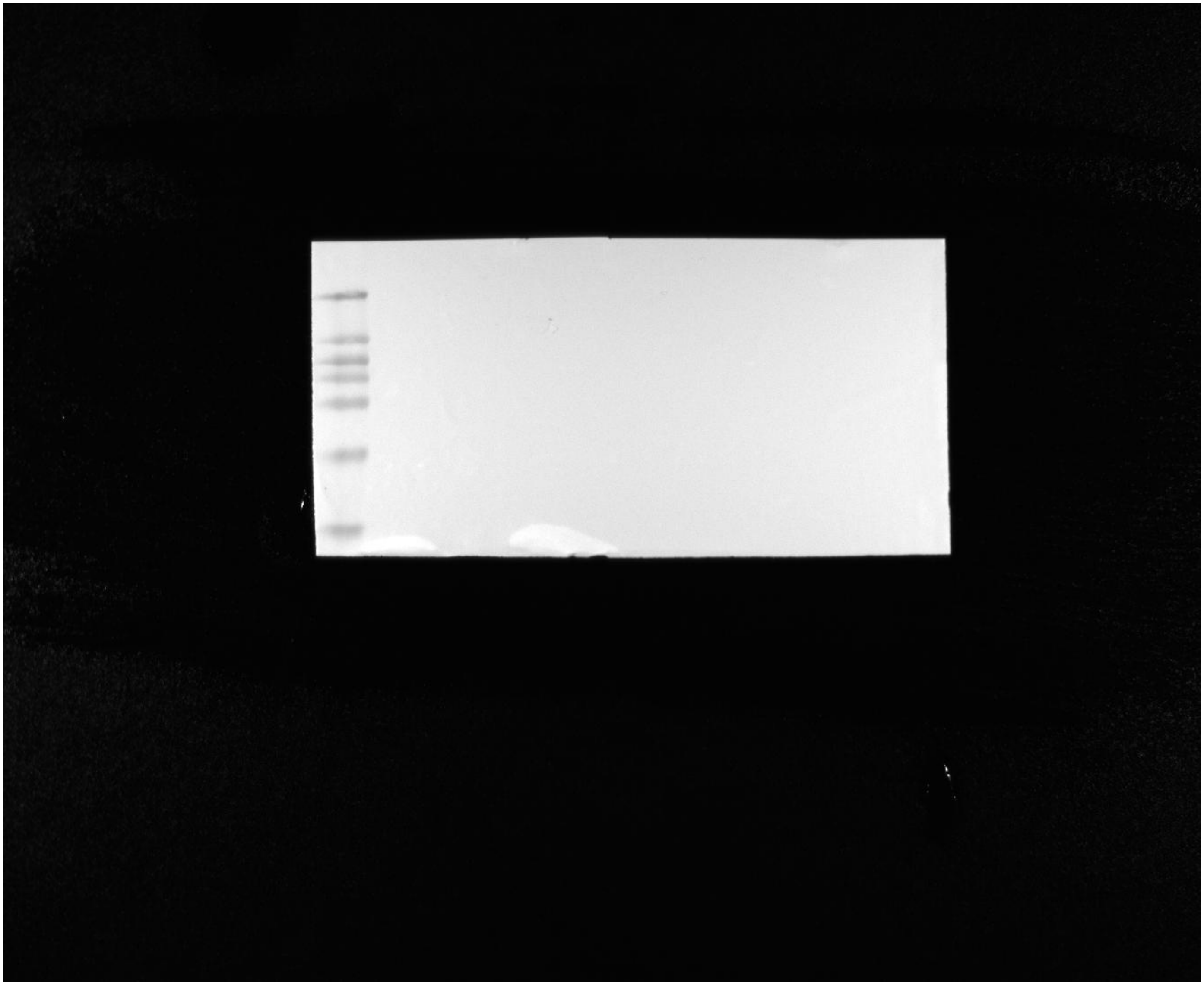

p-IRE1 PVDF image-1

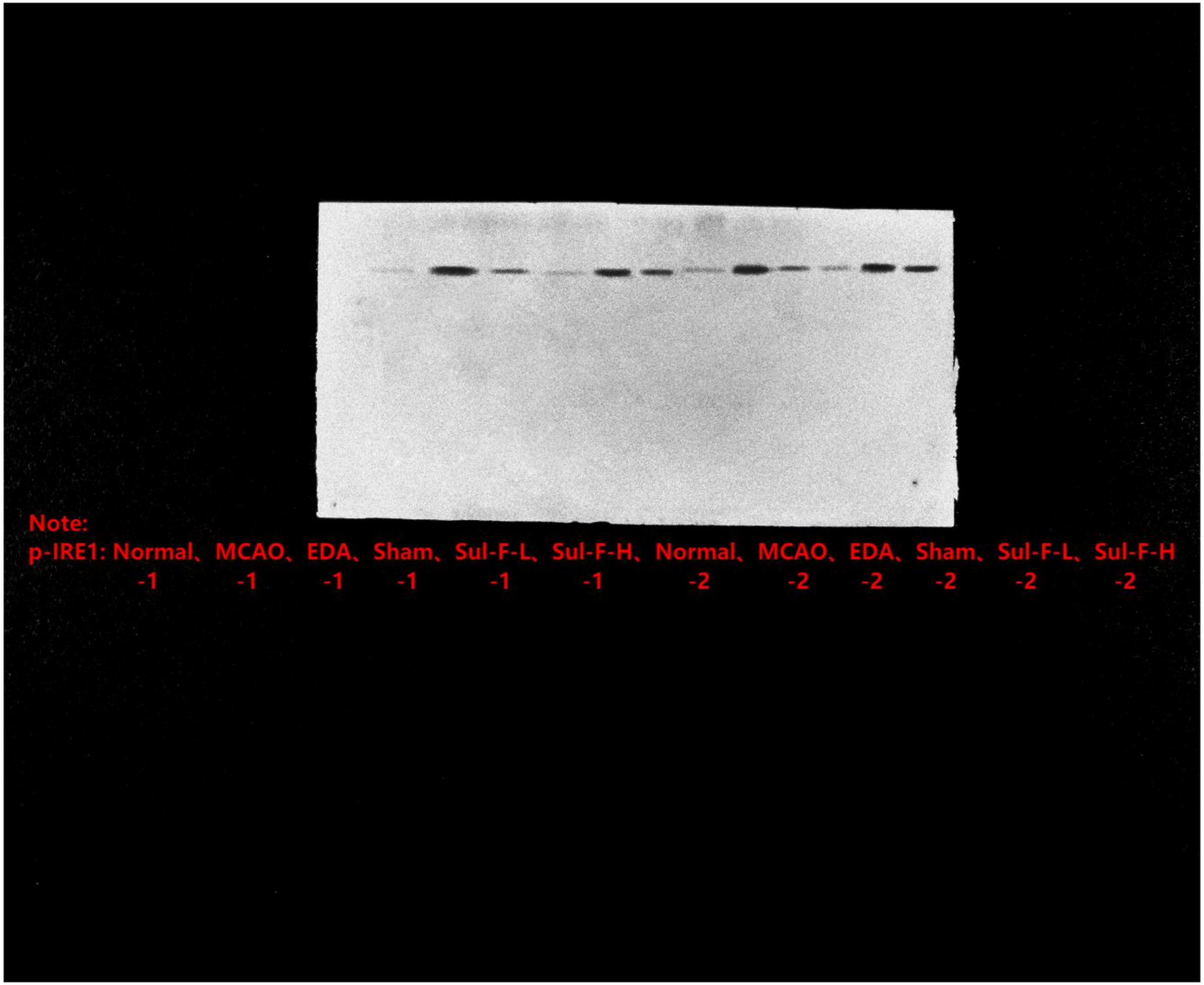

p-IRE1 Gel electrophoresis map-2

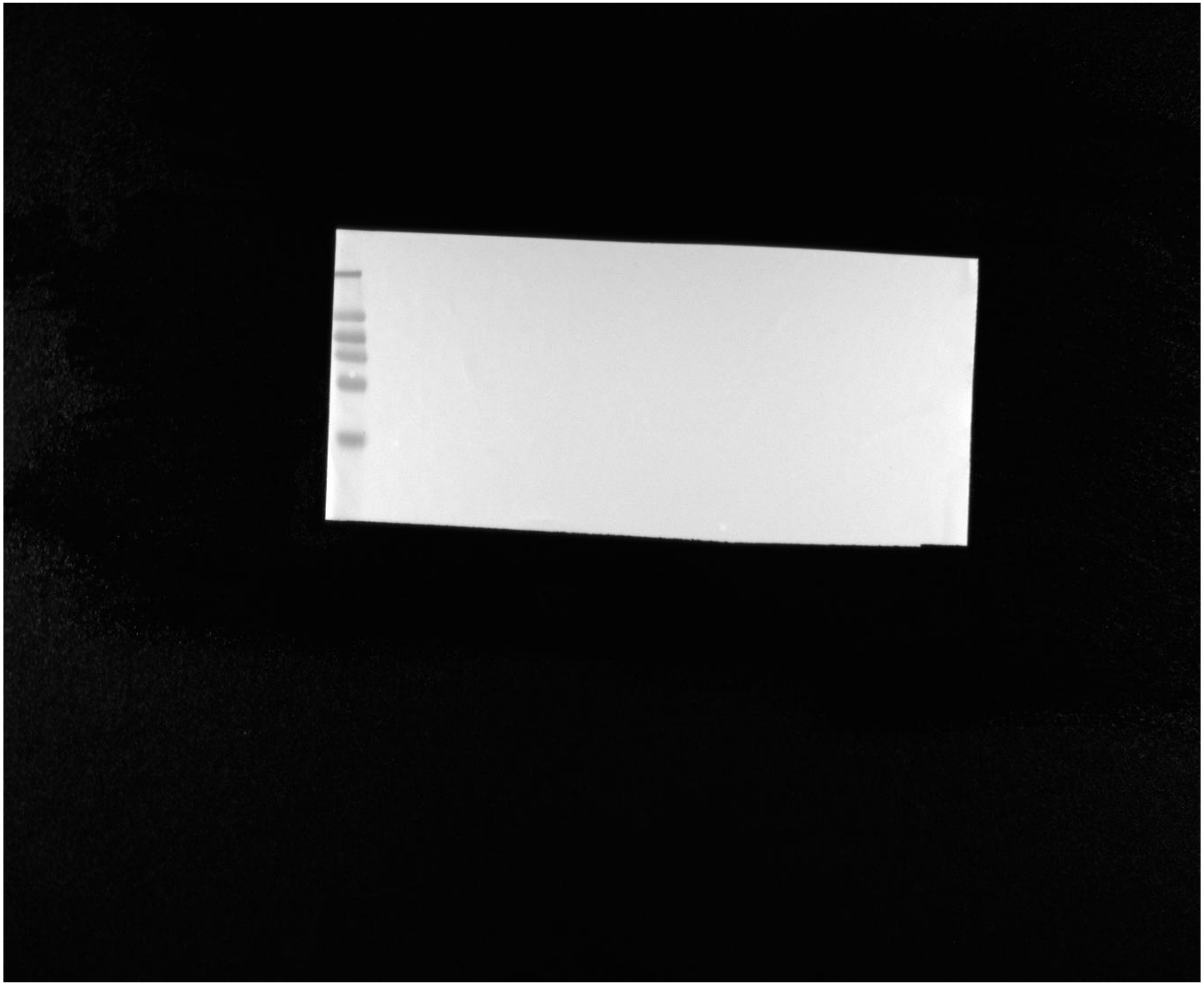

p-IRE1 PVDF image-2

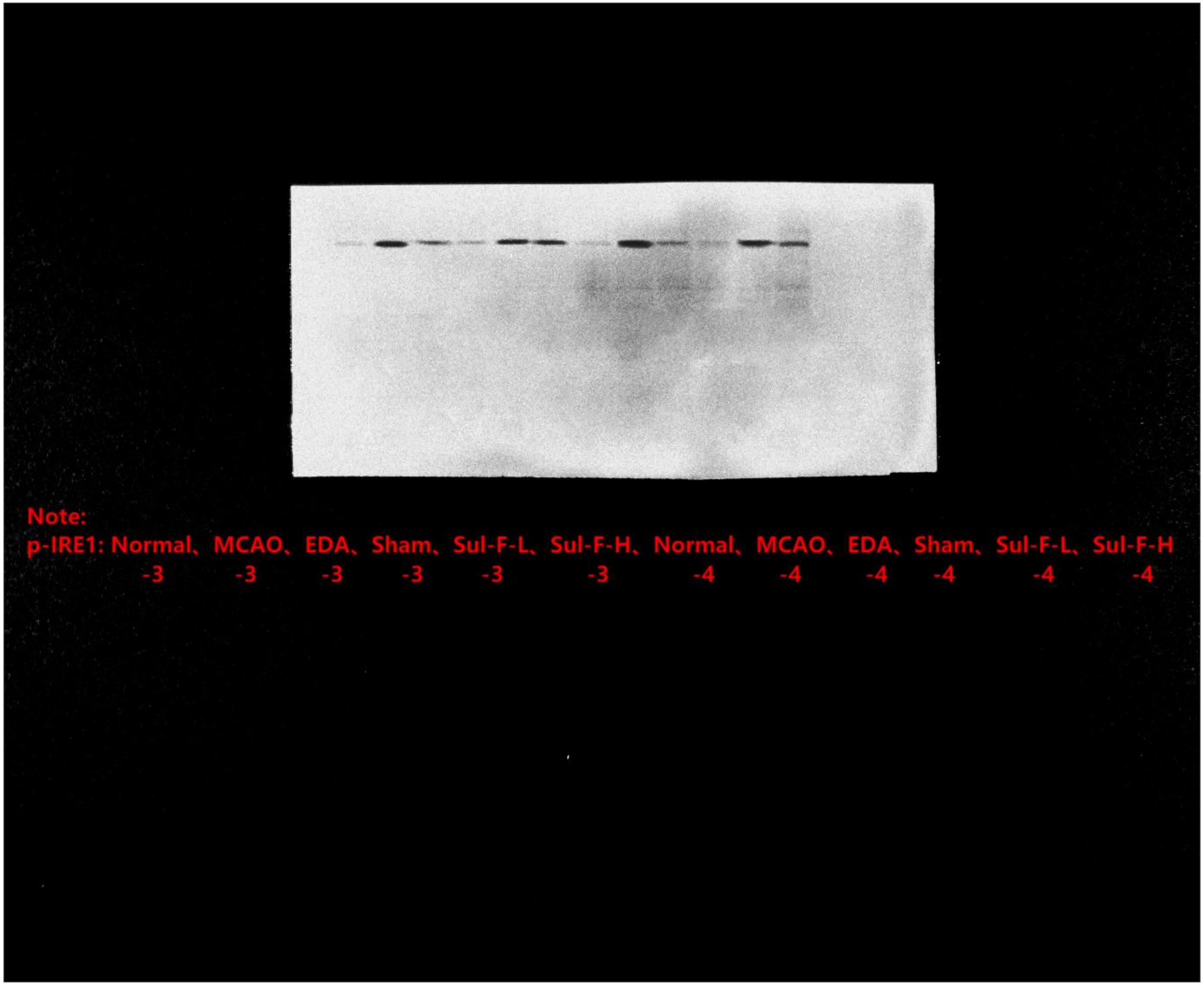

p-IRE1 Gel electrophoresis map-3

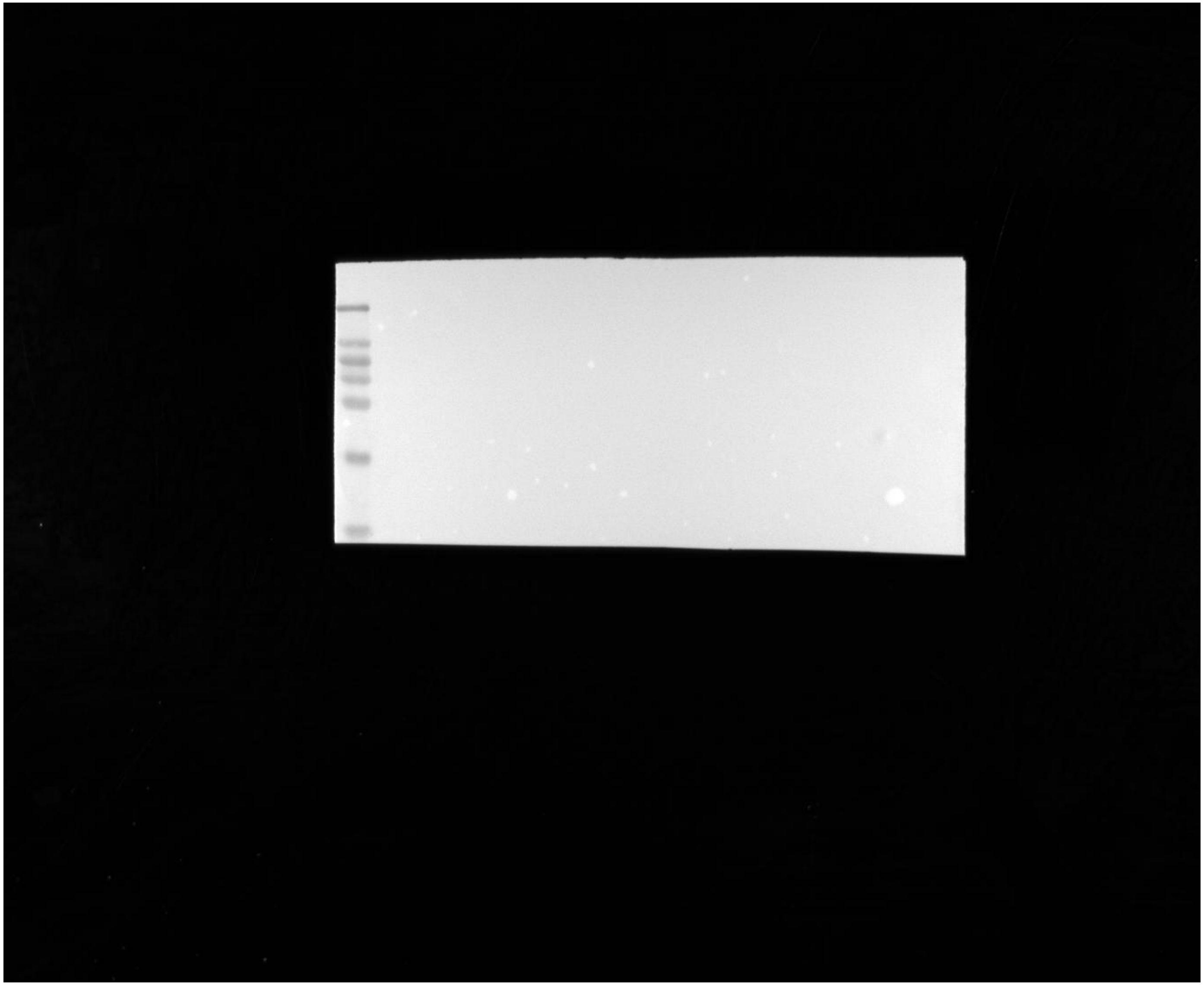

p-IRE1 PVDF image-3

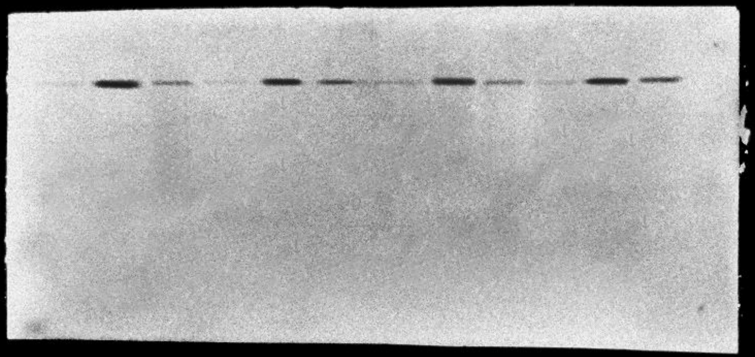

Note:
p-IRE1: Normal-5、MCAO-5、EDA-5、Sham-5、Sul-F-L-5、Sul-F-H-5、Normal-6、MCAO-6、EDA-6、Sham-6、Sul-F-L-6、Sul-F-H-6

Caspase12 Gel electrophoresis map-1

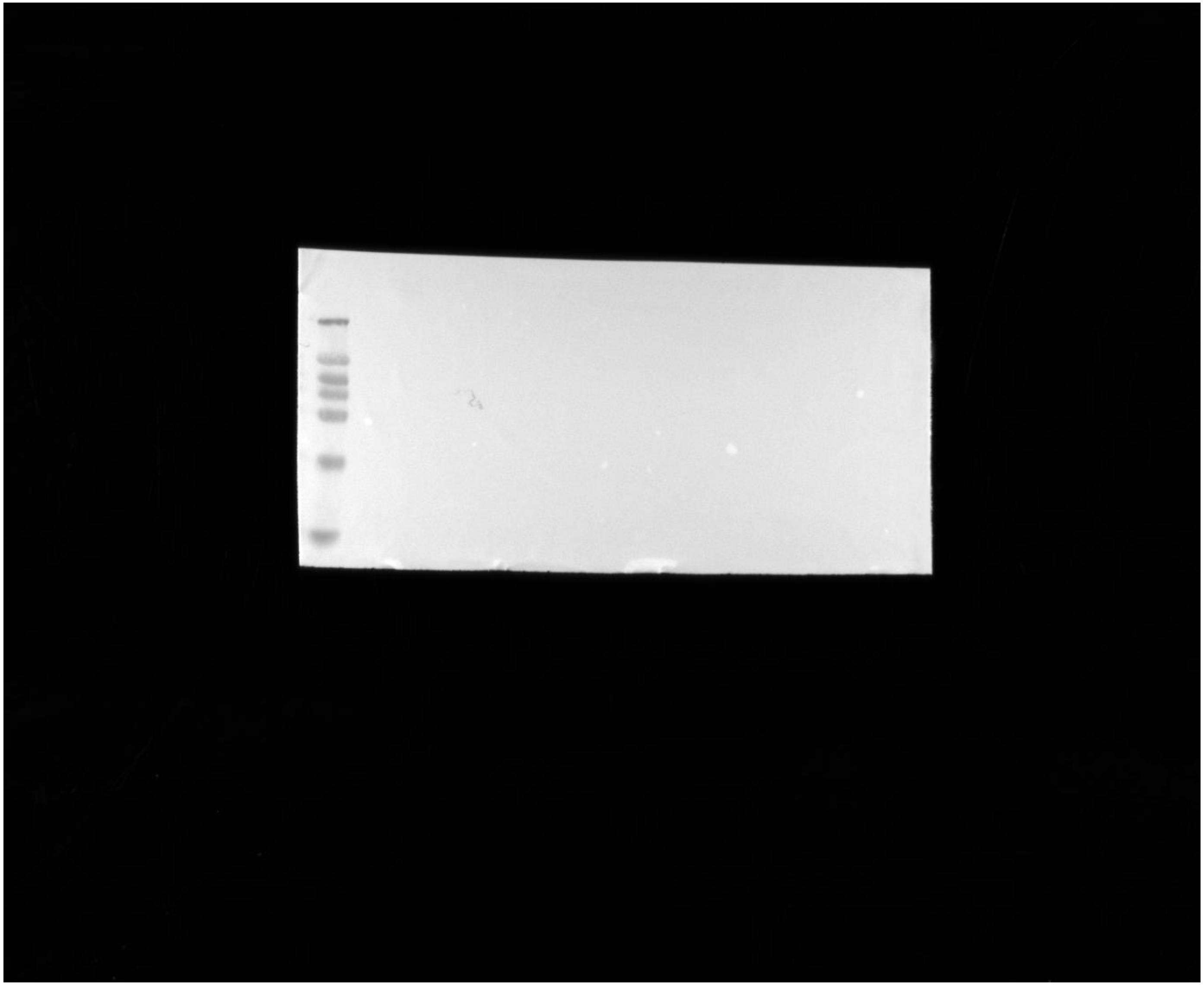

Caspase12 PVDF image-1

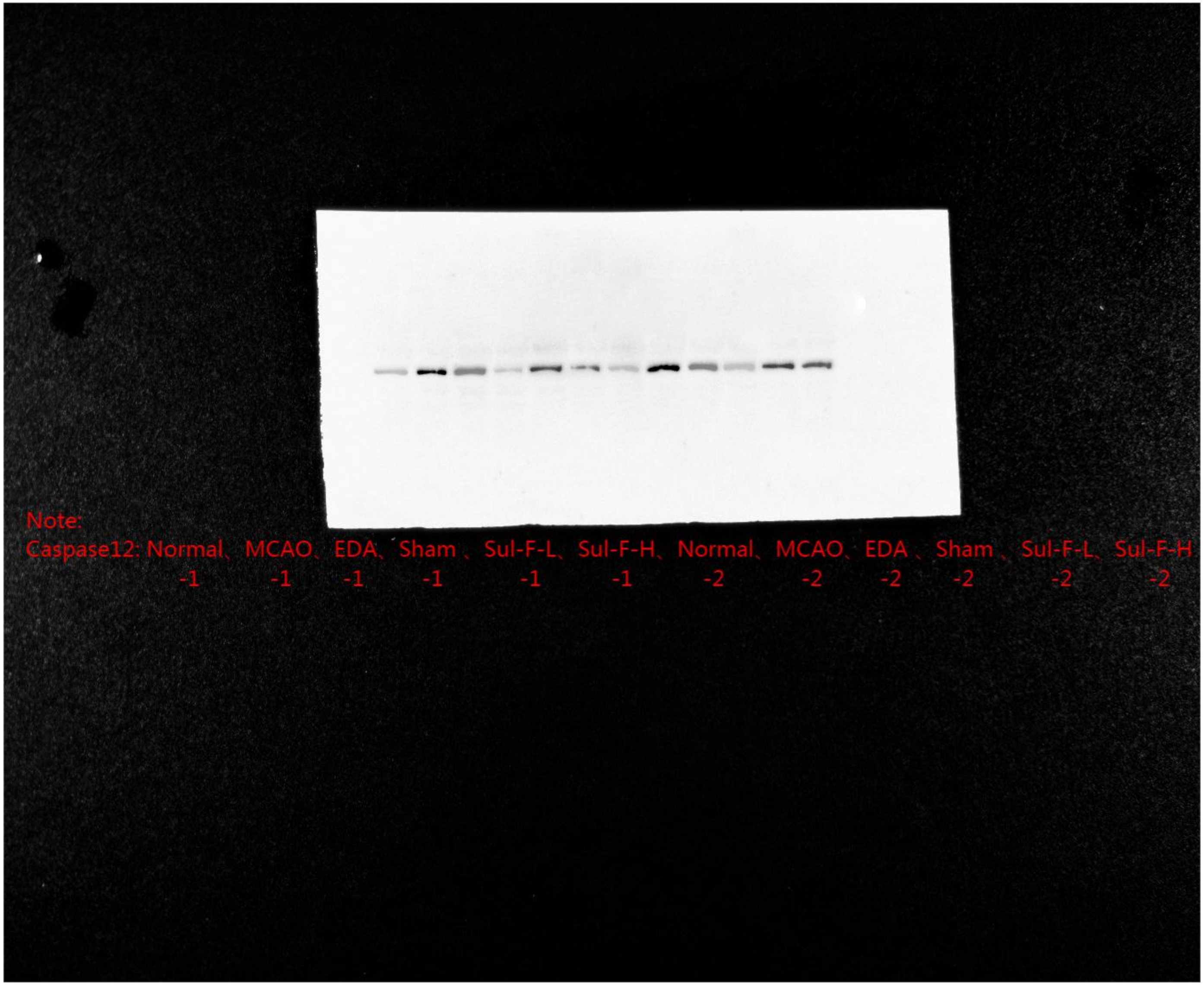

Caspase12 Gel electrophoresis map-2

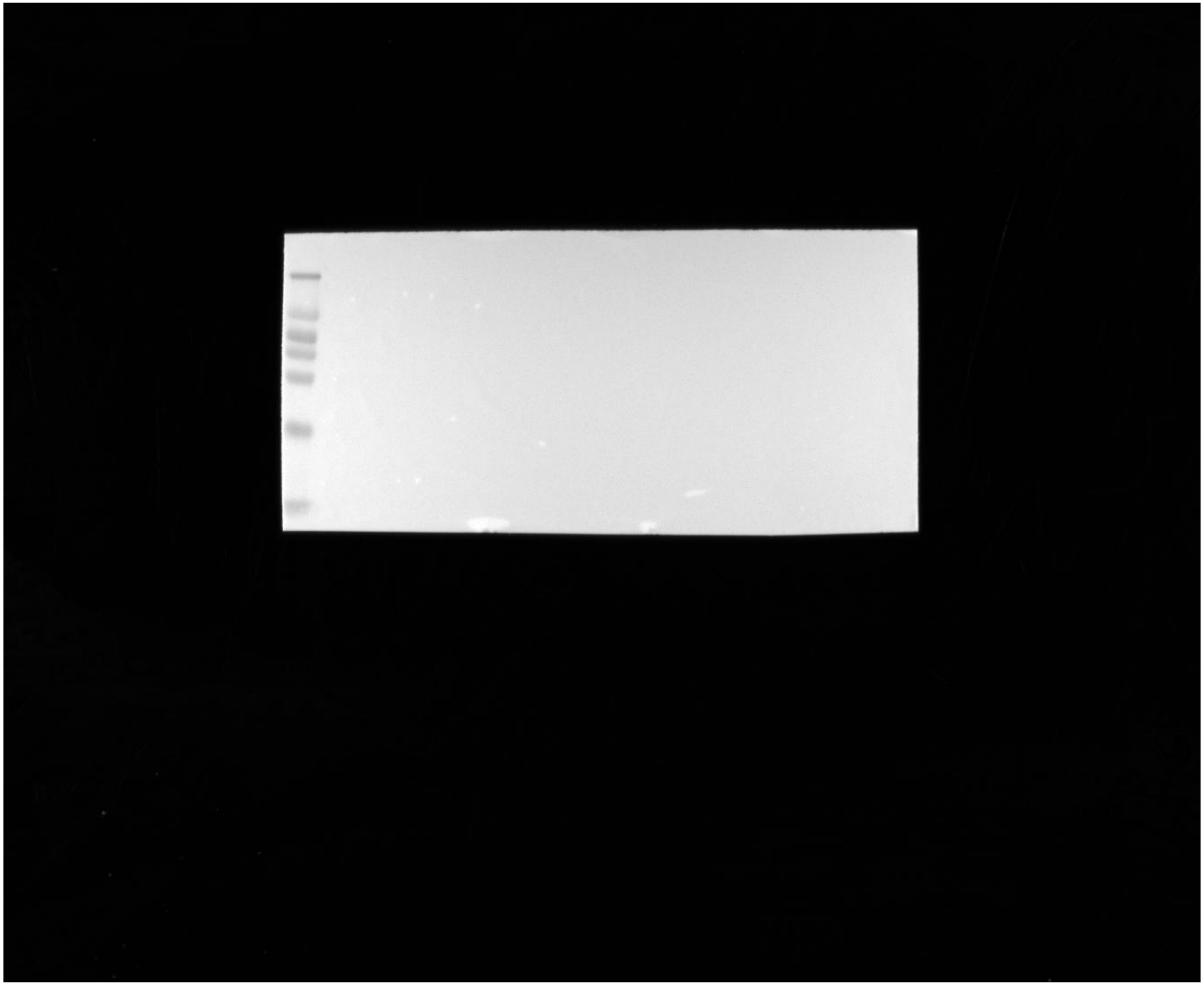

Caspase12 PVDF image-2

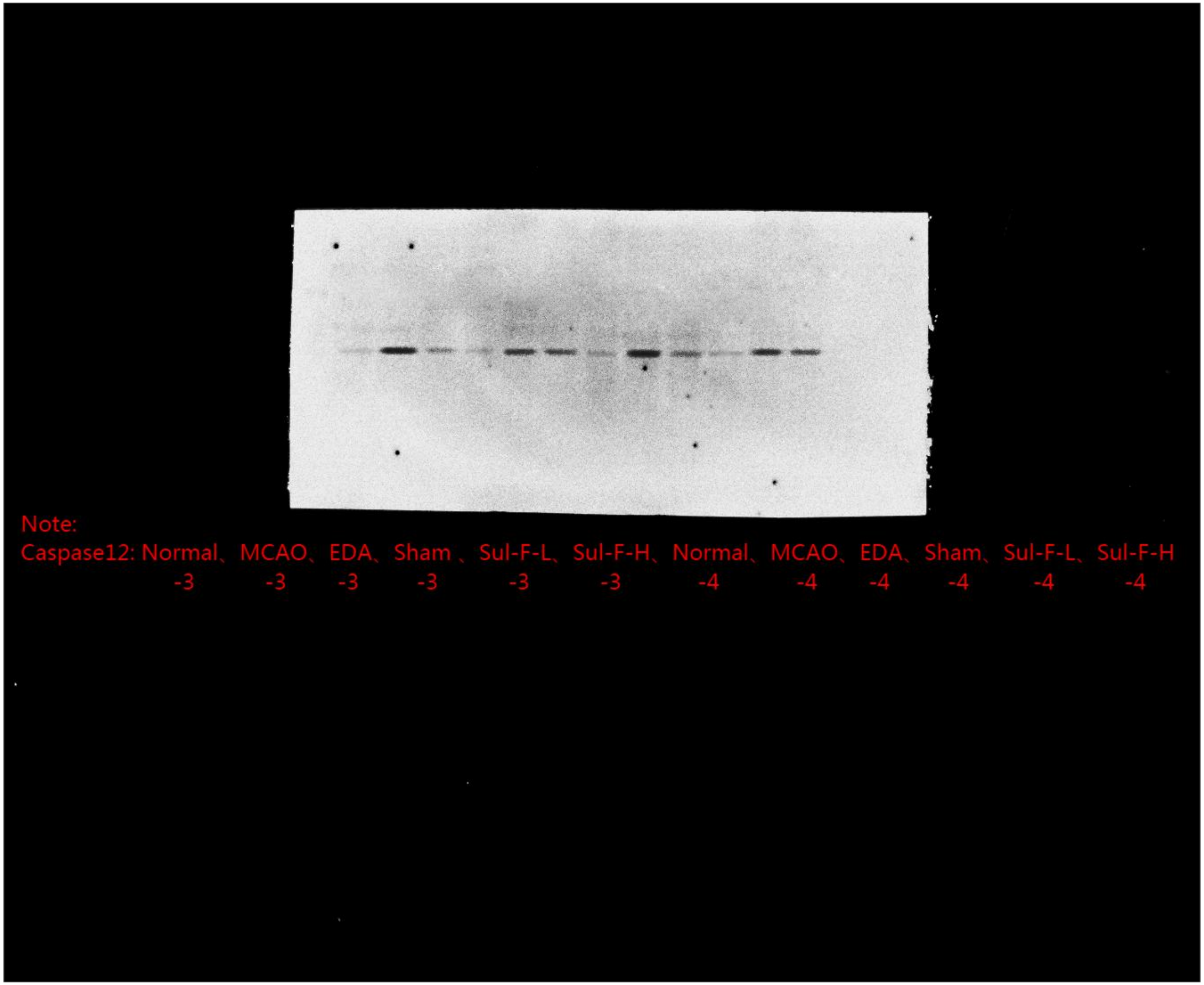

Caspase12 Gel electrophoresis map-3

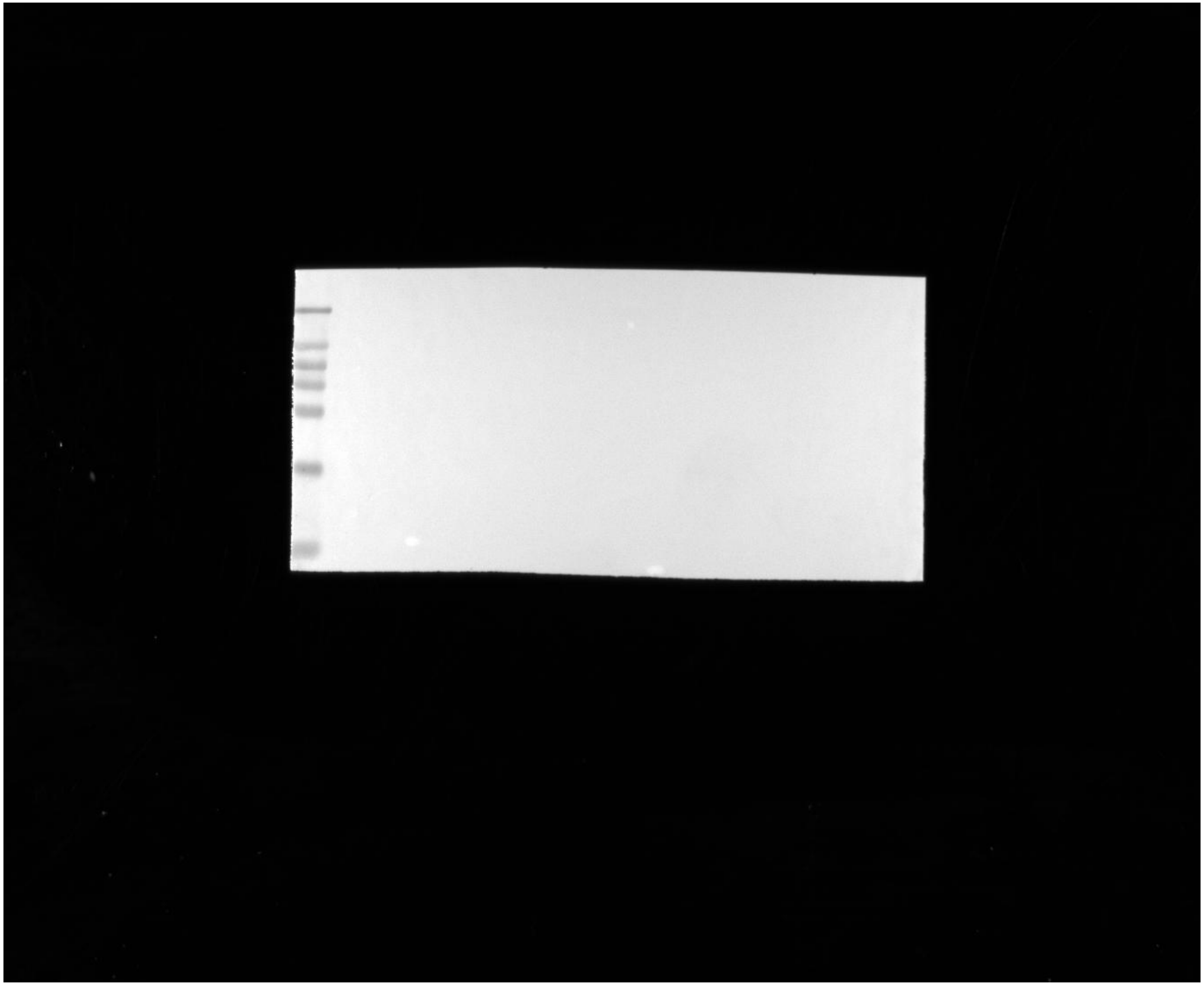

Caspase12 PVDF image-3

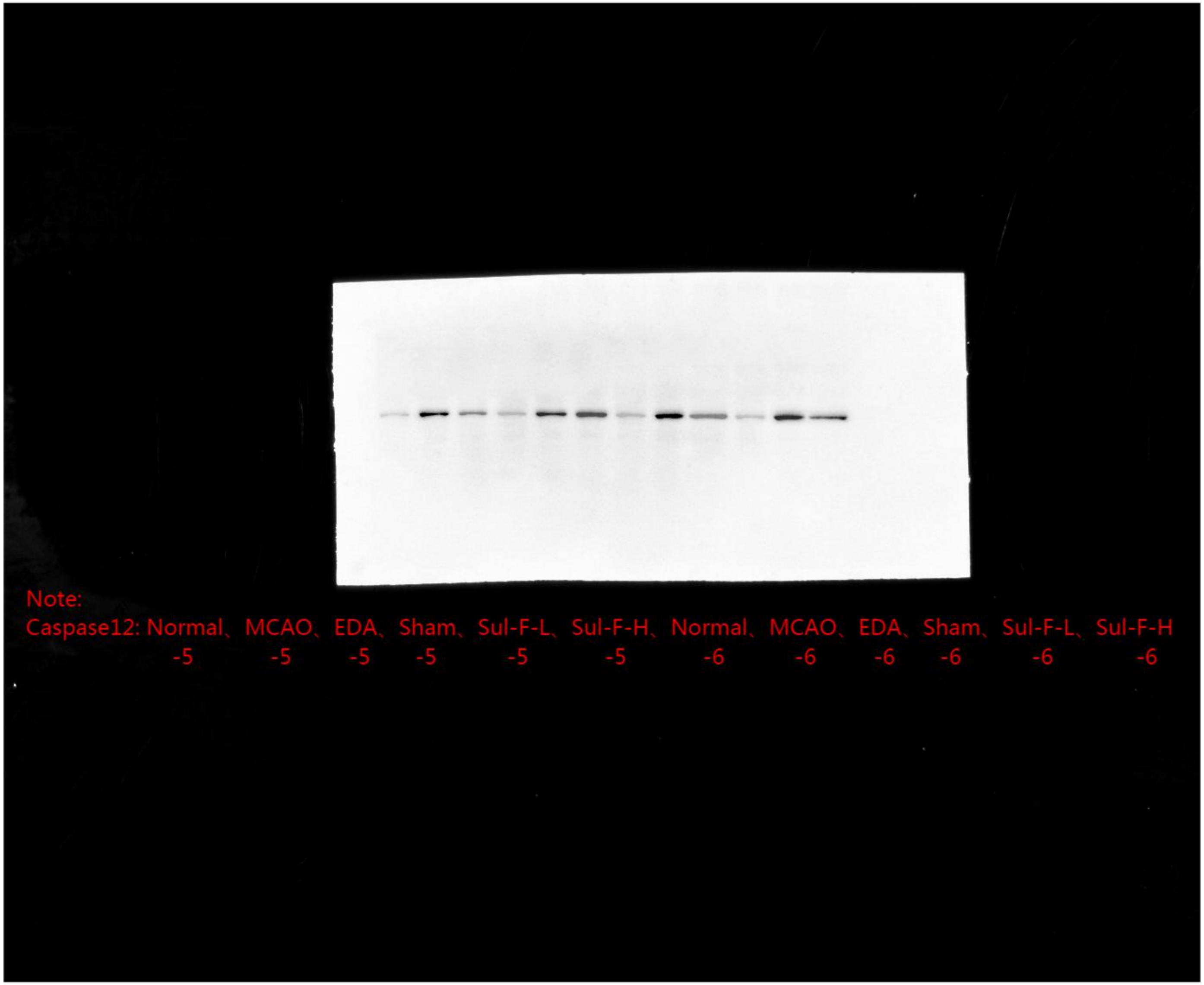

Caspase3 Gel electrophoresis map-1

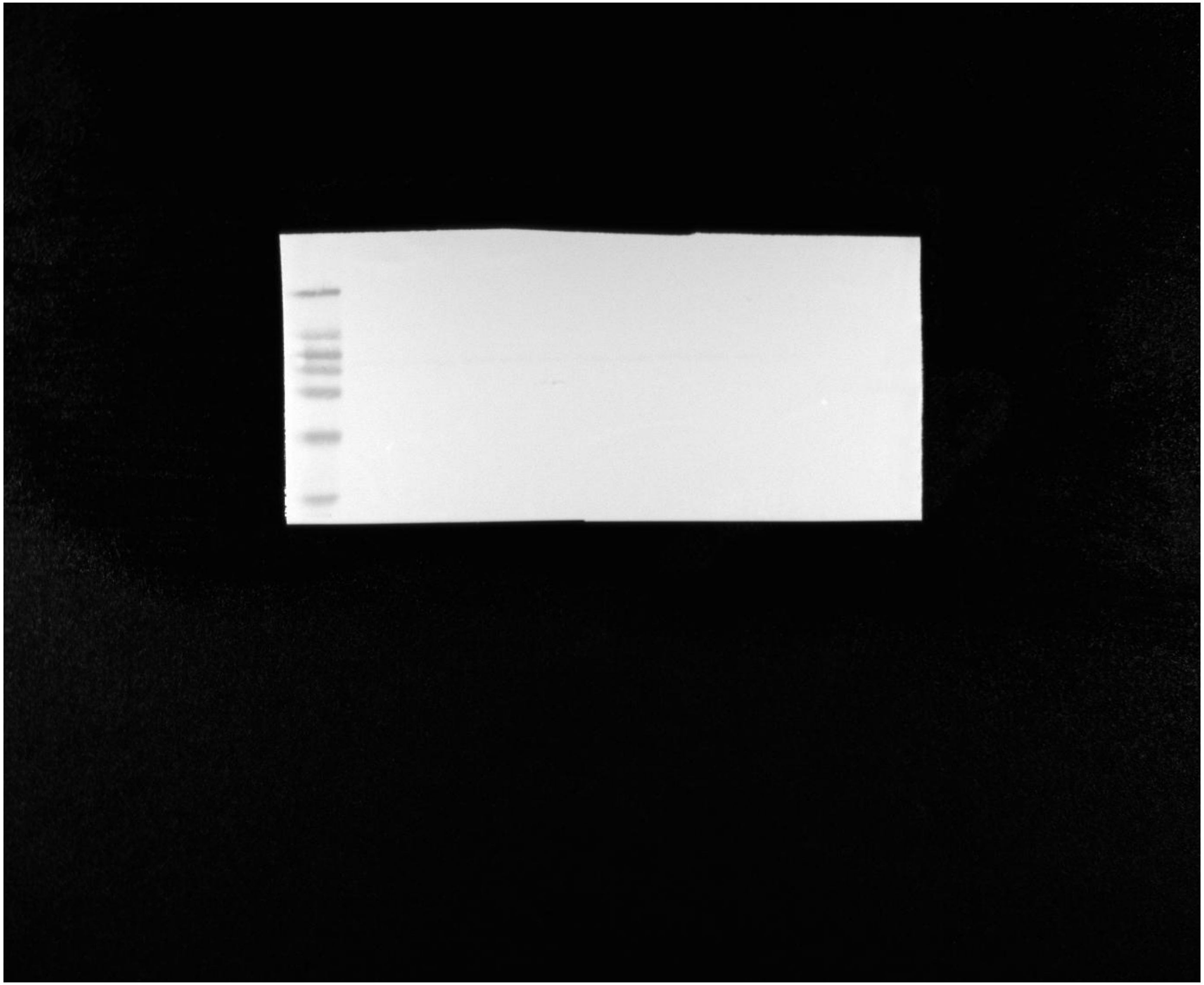

Caspase3 PVDF image-1

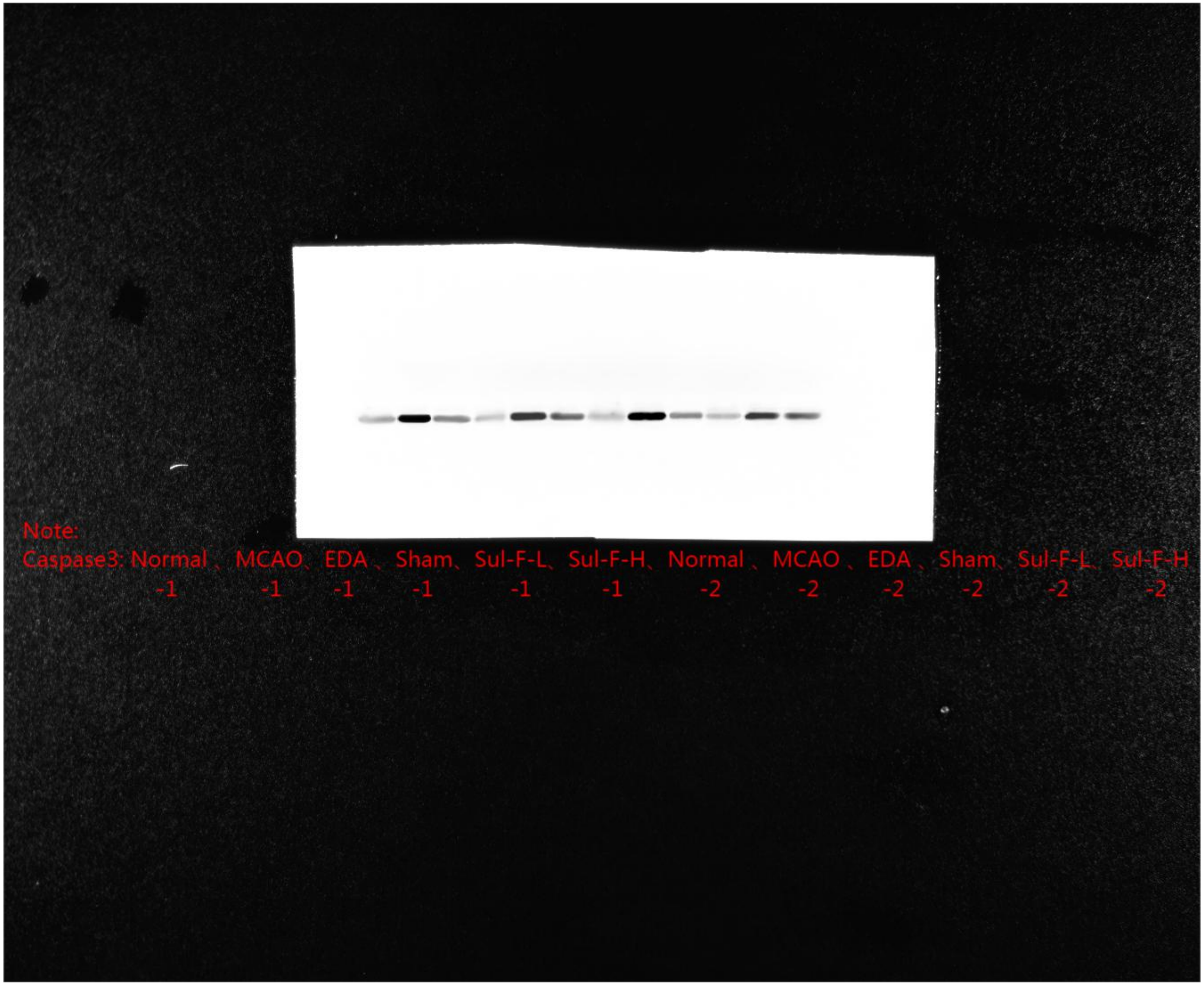

Caspase3 Gel electrophoresis map-2

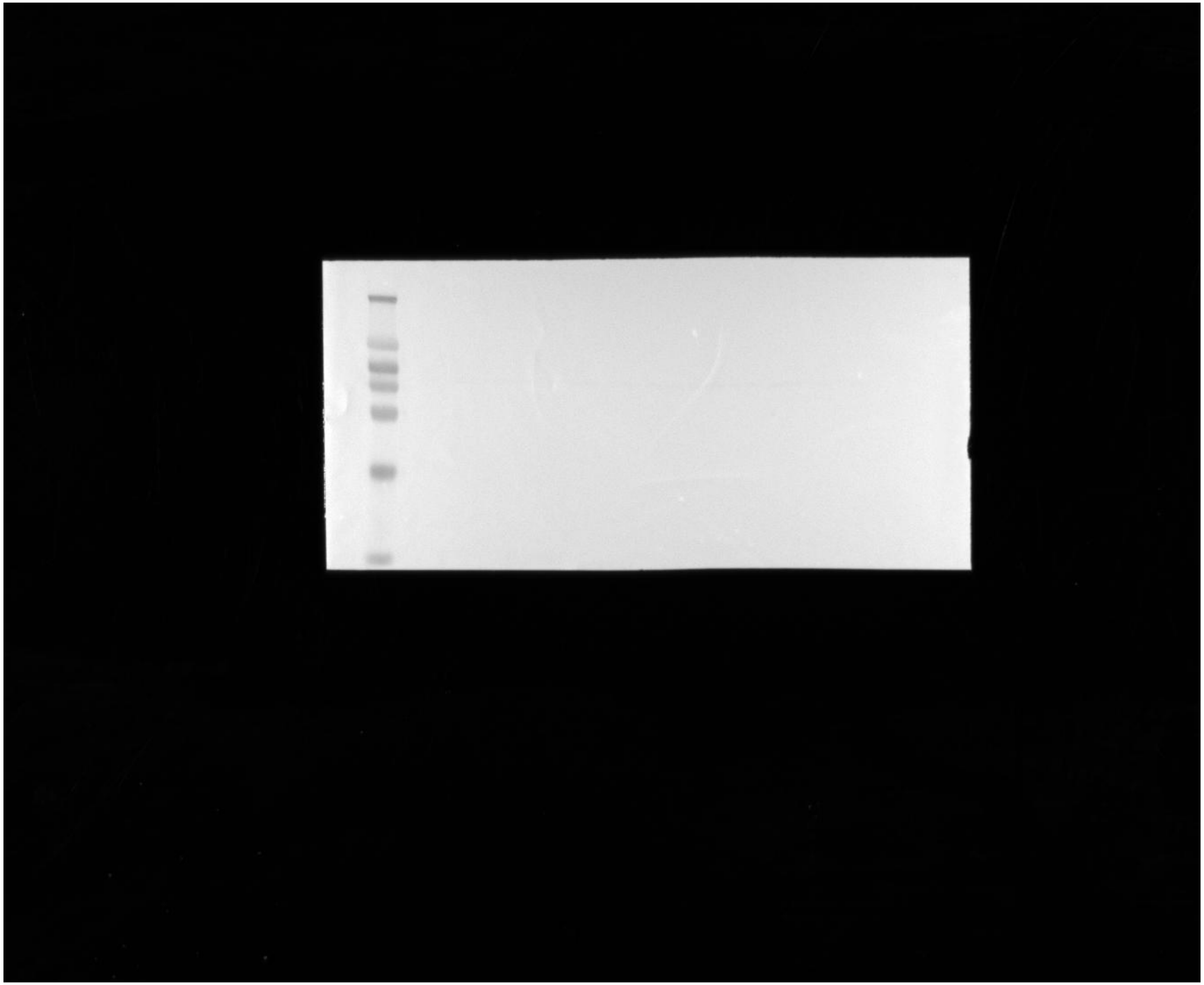

Caspase3 PVDF image-2

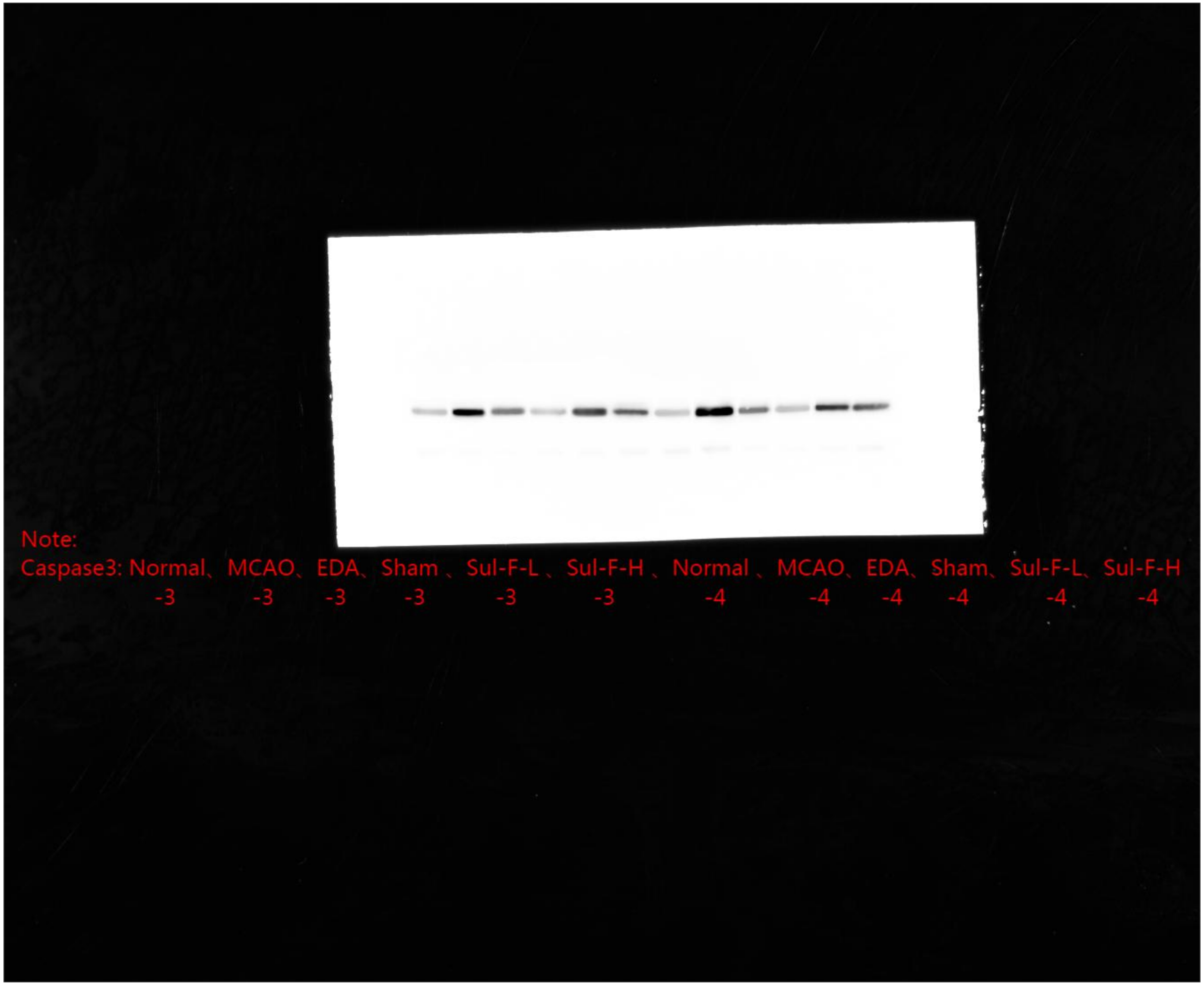

Caspase3 Gel electrophoresis map-3

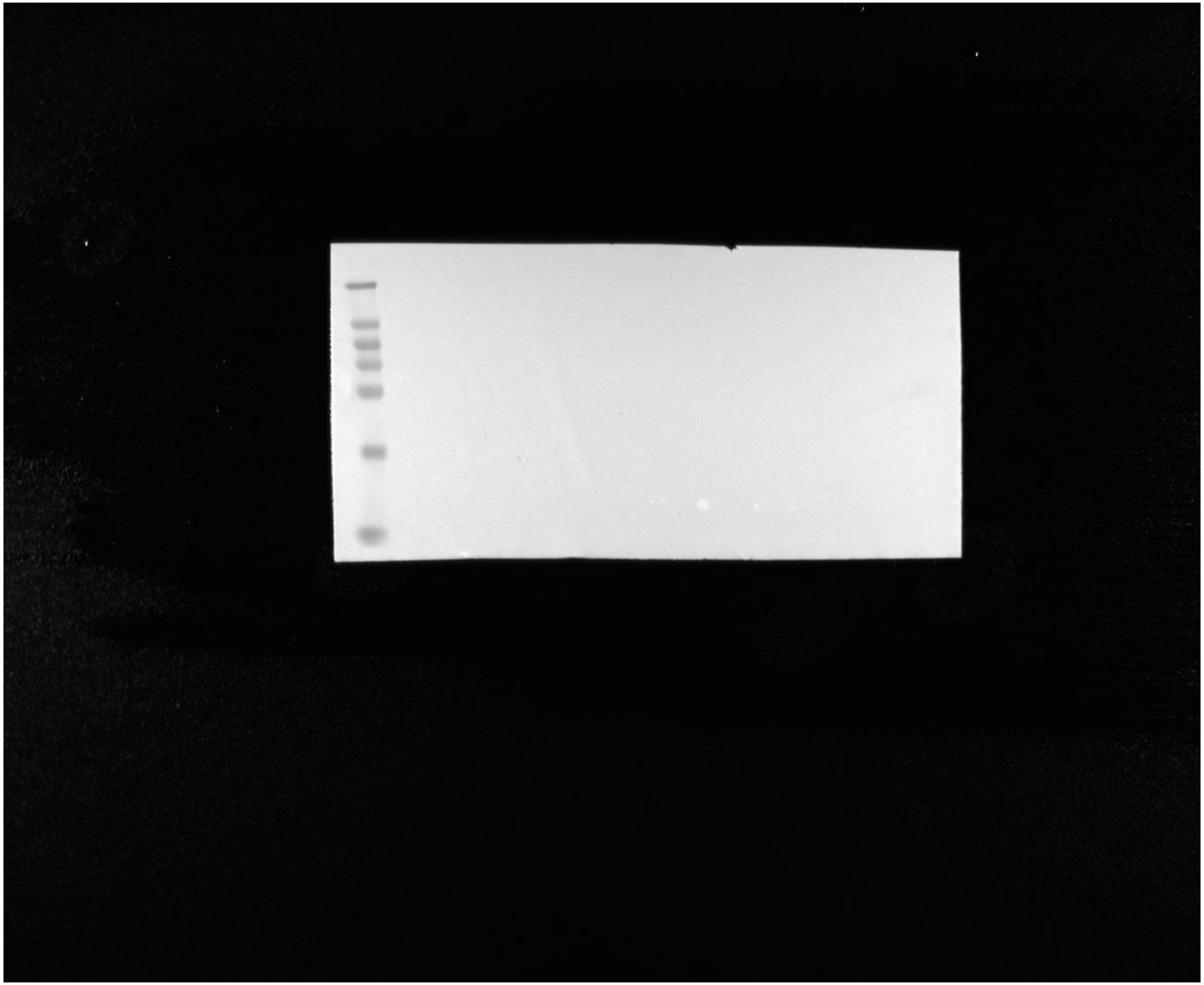

Caspase3 PVDF image-3

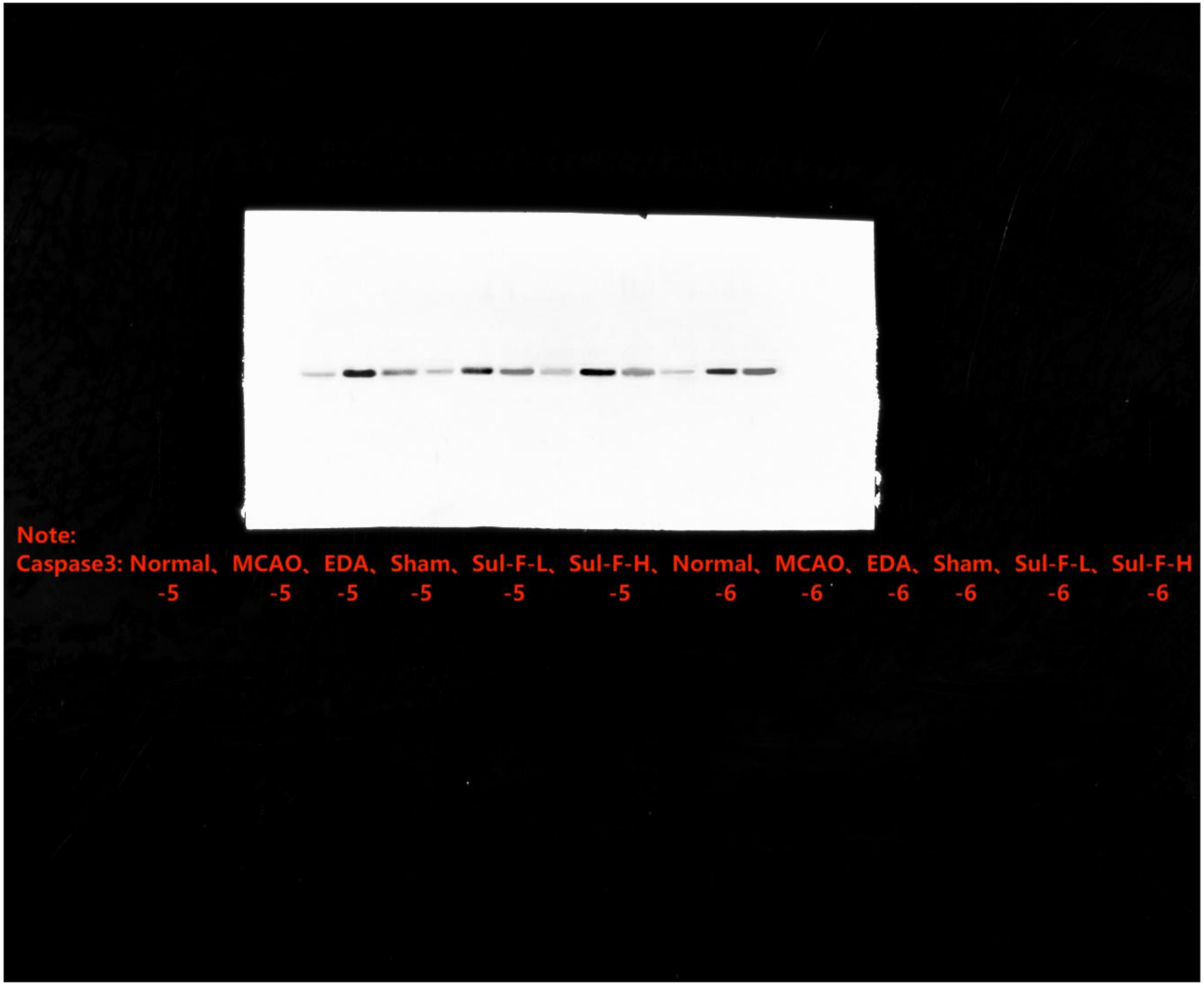

Supplement: Supplementary file 3 — Additional file 3: Gel electrophoresis map [file 12868_2022_762_MOESM3_ESM.pdf]
